# A comparative study of eggshells of Gekkota with morphological, chemical compositional and crystallographic approaches and its evolutionary implications

**DOI:** 10.1371/journal.pone.0199496

**Published:** 2018-06-22

**Authors:** Seung Choi, Seokyoung Han, Noe-Heon Kim, Yuong-Nam Lee

**Affiliations:** School of Earth and Environmental Sciences, Seoul National University, Seoul, South Korea; Perot Museum of Nature and Science, UNITED STATES

## Abstract

The Gekkota is an important clade in the evolution of calcified eggshells in that some of its families lay rigid eggshells like archosaurs. However, the fundamental differences and similarities between the mechanism of rigid eggshell formation of the Gekkota and Archosauria have not been investigated thoroughly due to the lack of knowledge of gekkotan eggshells. Here, we report for the first time a comprehensive analysis of morphological, chemical compositional, and crystallographic features of rigid and soft gekkotan eggshells. Exhaustive morphological description provided common characters for gekkotan eggshells, as well as unique features of each species. We found that elemental distribution of rigid gekkotan eggshells is different from that of avian eggshells, especially in the case of Mg and P. In addition, the crystallographic features (size, shape, and alignment of calcite grains) of gekkotan eggshells are completely different from those of archosaur eggshells. The result of this study suggests that soft gekkotan eggshells are morphologically more similar to tuatara eggshells rather than soft eggshells of derived squamates. The chemical compositional analysis suggests that the eggshell may act as a mineral reservoir for P and F as well as Ca. More importantly, all chemical compositions and crystallographic features imply that the gekkotan eggshell formation may begin at the outer surface and growing down to the inner surface, which is opposite to the direction of the archosaur eggshell formation. This character would be crucial for identifying fossil gekkotan eggs, which are poorly known in paleontology. All these lines of evidence support that soft gekkotan and tuatara eggshells share the primitive characters of all lepidosaurid eggshells. Finally, gekkotan and archosaur rigid eggshells represent a typical example of convergent evolution in the lineage of the Sauropsida.

## Introduction

**Fig 1:**
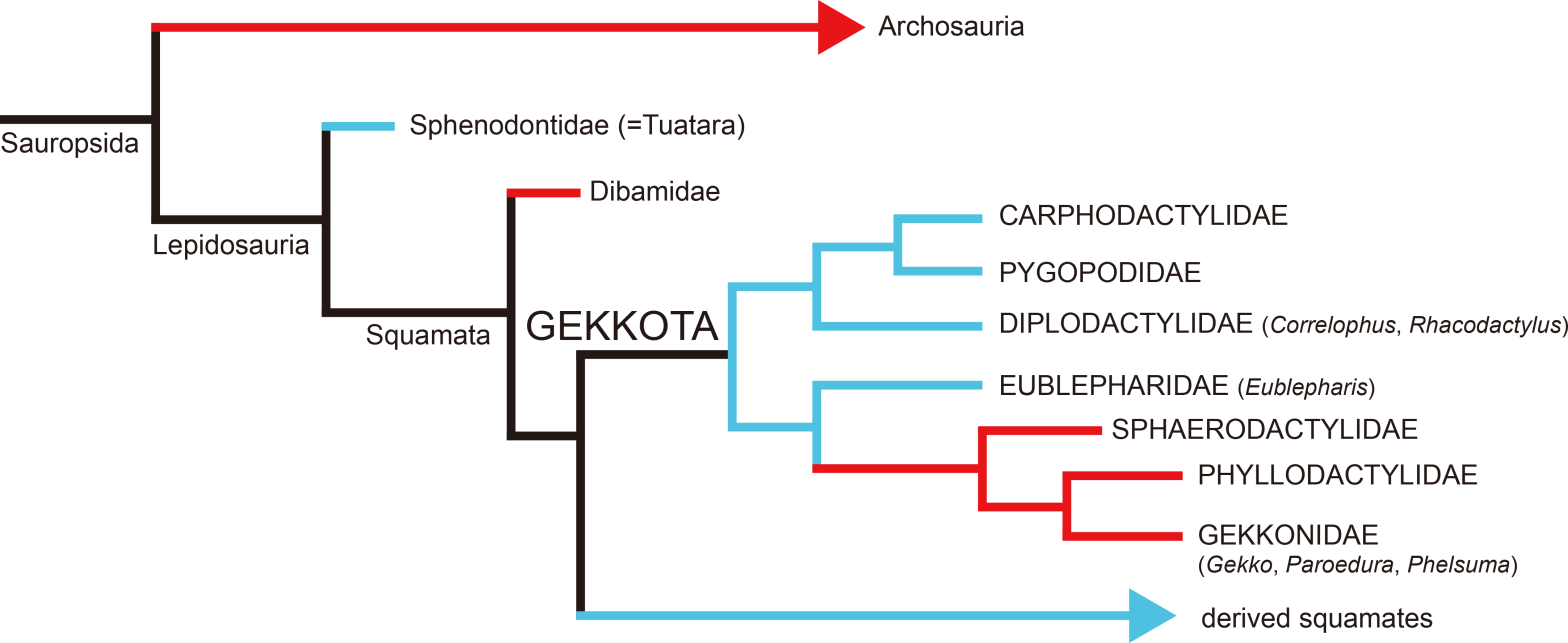
Phylogenetic relationship of the Gekkota and related major clades in the Sauropsida modified from [3,9,10]. The blue branches represent soft-shelled egg-layers while red branches rigid-shelled ones. The gekkotan genera used in this study are in parentheses.

The Squamata is the most diverse living clade of reptiles (nearly 9,200 species) [1]. Among them, the Gekkota is unique in egg formation because three families (Gekkonidae, Phyllodactylidae, and Sphaerodactylidae) lay rigid eggshells while the others (Carphodactylidae, Diplodactylidae, Eublepharidae, and Pygopodidae) lay soft eggshells like all other squamates except for the Dibamidae (legless lizard) (Fig 1; [1–4]). It has been argued that rigid gekkotan eggshells evolved from soft eggshells in the lineage of amniotes as with the case of Testudines, Crocodyliformes, and Dinosauria including birds [4–6]. However, except for the Gekkota and the Dibamidae, all sauropsids that lay rigid eggshells are archosaurs (a group that includes the most recent common ancestor of living crocodiles and birds and all its descendants) or closely related to archosaurs [6,7]. In this regard, except for the Dibamidae, while all non-gekkotan rigid egg-layers form a natural (= monophyletic) group (i.e., Archosauria), the Gekkota lies phylogenetically outside of this group [4,6]. Hence, the understanding of morphology, chemical composition, and crystallography of the extant gekkotan eggshells would be crucial when it comes to the investigation of the hypothesis on the common mechanisms of eggshell formation within the Gekkota and the Archosauria [8].

**Fig 2:**
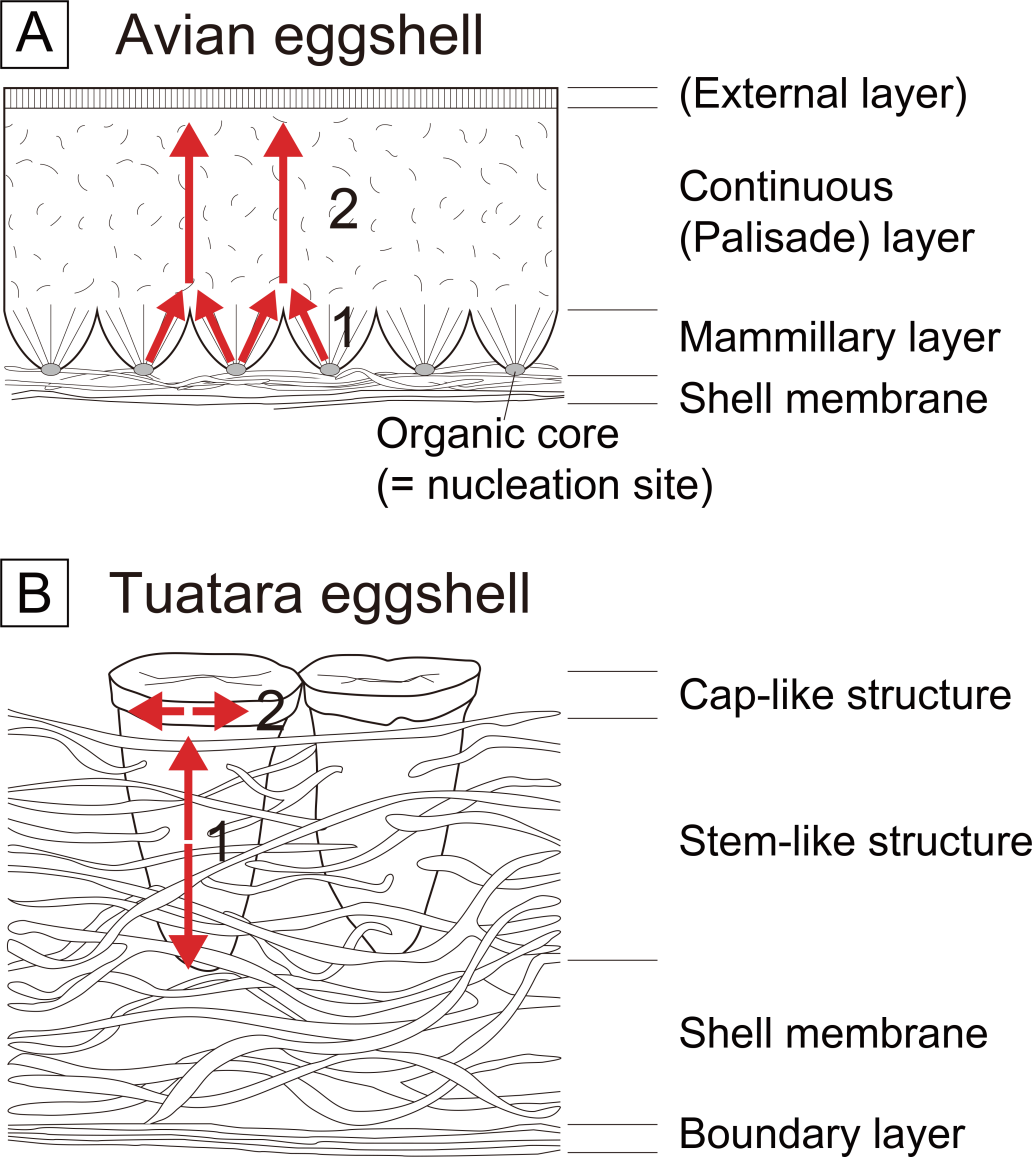
Two different eggshell growths in the Sauropsida. (A) In archosaur eggshells (represented by avian eggshell), eggshell formation begins at the organic core with radiating calcite grains (first step). At the end of the mammillary layer, the c-axis of calcite grains are aligned perpendicular to the eggshell surface and grow vertically (second step) [12,13]. (B) It was hypothesized that the mineralization of tuatara eggshell begins at the certain point of the stem-like structure, then proceeds to two opposite directions (both externally and internally; first step). When the formation of stem-like structure is completed, the formation of cap-like structure follows (second step). In the cap-like structure, the calcite grains grow horizontally [15]. Terminology followed for avian eggshell [14], and for tuatara eggshell [2,15].

All rigid eggshells of the Archosauria and the Testudines have the same mechanism of formation [8,11–13]. Eggshell formation initiates at the nucleation site of the external margin of the shell membrane, then crystals fan out externally to form a wedge-like (Crocodylia) or bump-like (Dinosauria including Aves) structure known as the mammillary layer in dinosaur eggshells (Fig 2A). After the initial radiating crystals are joined together at certain height, the crystallographically well-aligned crystals grow perpendicular to the eggshell surface and constitute a majority of the eggshell thickness. This layer is called a continuous (or palisade) layer [8,12–14]. On the other hand, the eggshell formation of Lepidosauria is poorly understood. The tuatara (basalmost Lepidosauria; Fig 1) eggshell formation mechanism was hypothesized to be completely different from that of archosaurs due to the fact that the shell unit of tuatara eggshell has a calcite “stem” deeply extended into the shell membrane (Fig 2B; [8,15]). This odd configuration of the shell membrane and calcite stem was unexplainable unless assuming the simultaneous deposition of shell membranes and calcite crystals partly growing to the inner direction [15]. It was once assumed that the rigid gekkotan eggs may have the same mechanism of eggshell formation as archosaur eggs [8]. In fact, however, the mechanism of eggshell formation has never been seriously studied for members of the Gekkota. They are crucial to understand how the rigid eggshell evolved in the Lepidosauria because the Gekkota is the only clade to lay both rigid and soft eggshells in the Squamata. In addition, the hypothetical convergent evolution [4,6] of rigid eggshells among the distantly related sauropsids (i.e., Gekkota and Archosauria) can be tested through this approach.

Diverse scientific approaches have been applied to gekkotan eggshells during the past two decades: measuring the relative amount of amino acids in eggshells [16], water vapor permeability [17–19], and evolutionary implications for egg shape and size [3,20]. However, fundamental studies for microstructural features of gekkotan eggshells have not been done exhaustively. For instance, several studies on eggshell morphology were conducted in the 1980s [2,21,22], but they mainly focused on the ultrastructural Scanning Electron Microscope imaging. Hirsch [23] pointed out that microstructural images observed in thin sections are crucial for herpetological and paleontological comparative studies of eggshells.

Recently, diverse scientific instruments have been used for understanding chemical composition or crystallographic characteristics in non-avian dinosaurs and extant avian eggshells (e.g., [12,13,24–31]). The results showed that the mechanism of eggshell formation is well-represented in the crystallographic information of the eggshell. Therefore, the application of combined classical and advanced methods to gekkotan eggshells would be helpful for comprehensive understanding of their morphology, rigid calcareous eggshell evolution among the sauropsids, and identification of gekkotan egg fossil in paleontology with certainty.

In this study, we conducted morphological, compositional, and crystallographic analyses using eggshells of seven extant gecko species within three families (Fig 1; S1 Table; see Materials and methods below). Morphological features attained by a Polarized Light Microscope and a Field Emission Scanning Electron Microscope (FE-SEM) are described in detail to provide specific characters of gecko eggs. The spatial variation of four crucial elements (Mg, P, S, and Ca) in the eggshells are demonstrated by using a Field Emission Electron Probe Microanalyser (FE-EPMA). Finally, the c-axis alignment of calcite crystals in the eggshells are represented with inverse pole figure (IPF) maps and lower hemisphere pole figures by using an Electron Backscatter Diffraction (EBSD) system. All the results showed unique characteristics of gekkotan eggshells, which are definitely different from archosaur eggshells. Finally, the evolutionary implications of this study are discussed with special emphasis on symplesiomorphy of basal lepidosaurian eggshells, convergence between the archosaur and gekkotan eggshells, and possible parallel evolution among the several clades in squamates.

## Materials and methods

### Eggshells of seven species of extant Gekkota

The rigid eggshells of four species (Gekkonidae: *Gekko gecko*, *Paroedura pictus*, *Paroedura stumpffi*, and *Phelsuma grandis*) and soft eggshells of three species (Diplodactylidae: *Correlophus ciliatus* and *Rhacodactylus leachianus*; Eublepharidae: *Eublepharis macularius*) of geckos, which were available to the authors, were used in this study (Fig 1; S1 Table). The eggs of *Gekko gecko* were provided by the National Institute of Ecology in South Korea. The eggs of *Paroedura pictus*, *Paroedura stumpffi*, *Phelsuma grandis*, *Correlophus ciliatus*, *Rhacodactylus leachianus*, and *Eublepharis macularius* were provided from a pet shop in Seoul. Except for eggshells of *Phelsuma grandis*, all specimens were hatched eggs so that calcite portions of these eggshells were probably affected during the embryogenesis [8,32,33]. In addition, the micro- and ultra-structural characters of soft eggshells might have been affected by water intake during the incubation [8,19]. Each taxon is assigned a letter of the alphabet in all relevant figures: A, *Gekko gecko*; B, *Paroedura pictus*; C, *Paroedura stumpfii*; D, *Phelsuma grandis*; E, *Correlophus ciliatus*; F, *Rhacodactylus leachianus*; G, *Eublepharis macularius*. All studied samples are stored in the Paleontological Laboratory in Seoul National University.

### Thin section preparation

The thin sections were prepared following the standard thin section preparation method of eggshell fossils [34]. The eggshells were embedded in epoxy resin and were cut to expose the radial sections of the eggshells. The rough section was hand lapped on a glass plate with 600-, 1000-, and 3000-grit aluminum compound and glued to a slide glass. The opposite sides were cut using a circular blade to approximately 90 μm and were hand lapped to a final thickness of 30 μm. After lapping, thin sections were polished with 0.5 μm diamond paste. These prepared thin sections were examined and photographed using a polarized light microscope (Nikon Eclipse LV100N POL), housed at the School of Earth and Environmental Sciences (SEES) of Seoul National University (SNU).

### FE-SEM

For Field Emission Scanning Electron Microscope (FE-SEM; JEOL JSM-7100F) observation, rigid eggshells were fractured using the tweezers, coated with carbon and were mounted on an aluminum stub with carbon tape. In case of soft eggshells, materials for radial section observation were frozen in the liquid nitrogen and then cut in the same way in order not to make its fibrous proteins disheveled [21]. Fresh radial section, inner, and outer surfaces were examined in Secondary Electron (SE) mode. In addition, we lapped an eggshell cross section embedded in epoxy resin with 600-, 1000-, and 3000-grit aluminum compound, polished with 0.5 μm diamond paste, coated with carbon, and mounted on an aluminum stub with carbon tape. These materials were observed using Backscattered Electron (BSE) mode of SEM to see the distribution of calcareous materials and the proteins [35,36]. The setting of the SEM was as follows; accelerating voltage 15.0 kV, an emission current 46.40 μA, and working distance 10 mm. The FE-SEM is housed at the SEES, SNU.

### FE-EPMA and EDS

Eggshell chemical compositions of each species were analyzed using Field Emission Electron Probe Microanalyser (FE-EPMA; JEOL JXA-8530F) housed at the National Center for Inter-University Research Facilities of SNU. The materials were prepared as for BSE observation, coated with platinum, and mounted on an aluminum stub. The elemental mapping and line profile analyses were conducted. The distribution of five elements (Na, Mg, P, S, and Ca) which are known to function in the avian eggshell formation (Na, Mg, and P; [12,24,37]) or at least present in the avian or lizard eggshells (Mg, S, and Ca; [30,38,39]) were analyzed. The analyzed data of line profile analyses were calibrated using mineral standards: Na (Jadite, NaAlSi_2_O_6_), Mg (Periclase, MgO), P (Potassium titanium phosphate, KTiOPO_4_), S (Pyrite, FeS_2_), and Ca (Scheelite, CaWO_4_). Elemental profiles were created by averaging several individual line profiles for which the shells were approximately the same thickness. This selection was necessary in order to avoid the profile distortion caused by the difference in thickness. The setting of element mapping and line profile analysis was as follows; accelerating voltage 15.0 kV, emission current 20 nA, dwell time 30.00 ms, pixel 256 X 256.

In order to test the hypothesis that high concentration of P is present in a separate layer inside the eggshell (see discussion below), additional Energy-Dispersive X-ray Spectroscopy (EDS) analyses were conducted in rigid gekkotan eggshells. EDS (X-Max, Oxford Instruments) is attached to the FE-SEM (JEOL JSM-7100F), which is housed at the SEES, SNU. The setting of EDS analysis was as follows; accelerating voltage 10.0 kV, emission current 46.40 μA, and working distance 10 mm.

### EBSD

For Electron Backscatter Diffraction (EBSD) analysis, we mainly followed the method of [13]. The specimens were prepared as for BSE image preparation step and polished with colloidal silica (0.06 μm) for 20 minutes for each specimen. After that, each specimen was coated with carbon. The EBSD analysis was performed by using the Symmetry Detector (Oxford Instruments) attached to the FE-SEM (JEOL JSM-7100F) in the SEES, SNU. The Kikuchi lines were acquired and indexed automatically using Oxford AZtec software with a step size of 0.2–1.0 μm depending on the dimensions of the eggshell. The EBSD data were presented in both inverse pole figure (IPF) maps and lower hemisphere pole figures. After the acquisition of IPF maps, noise reduction steps were applied: wild spike elimination and most conservative way of zero solution correction (i.e., if one zero solution is surrounded by at least six consistent signals, the zero solution is treated like them). {0001} pole figures show the orientation of c-axis. {11–20} and {10–10} show the a- and b-axes orientation, respectively [13]. All pole figures are represented in equal area projection. The half width of 20 degrees and a cluster size of 5 degrees were used in contouring. The setting of the EBSD analysis was as follows; accelerating voltage 15.0 kV, working distance 25.0 mm, 70 degrees tilting of specimens. The measurements from micrographs were made using ImageJ for more than 30 times and the average was presented.

### Terminology

**Fig 3:**
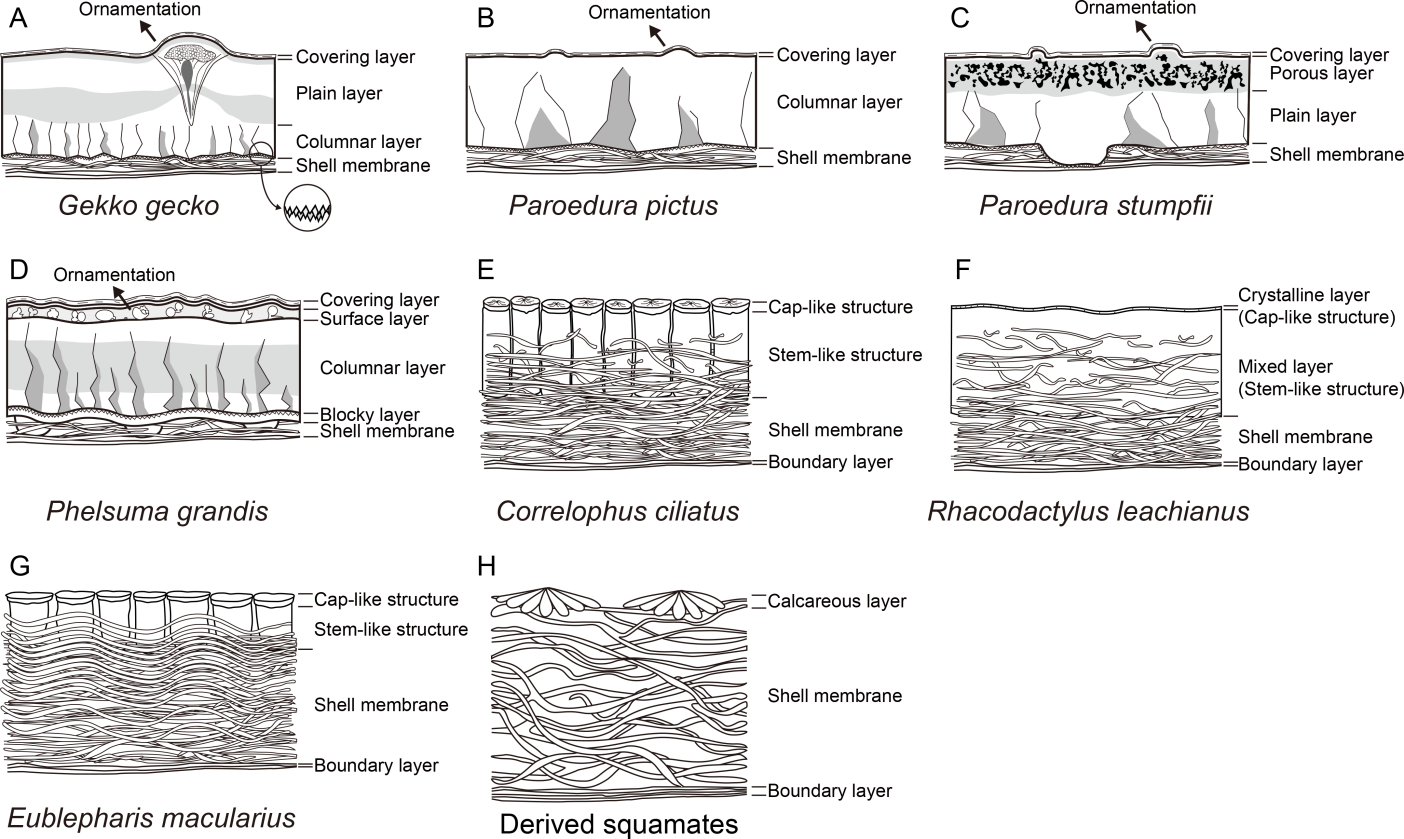
Schematic views of the seven different gekkotan and representative derived squamate eggshells. Most of the terminology followed [2,15] and otherwise new terms are proposed in this study. The light gray areas in *Gekko gecko* (A), *Paroedura stumpfii* (C), and *Phelsuma grandis* (D) eggshells show the location and shape of “dark bands”. The dark gray areas in rigid gecko eggshells (A–D) signify the shape of extinction pattern under polarized light. The jargon “calcareous layer” is used in soft gekkotan eggshell to refer to stem-like and cap-like structures, collectively. The figures are not drawn to scale. In *Gekko gecko* eggshell (A), a pore-like and bulbous structures are associated with the ornamentation. The eggshell of *Paroedura stumpfii* has a peculiar porous layer (C). The mixed layer of *Rhacodactylus leachianus* eggshell (F) can be further differentiated into two sub-layers. The protein fibers in *Eublepharis macularius* eggshell (G) are undulated unlike other soft eggshells (E, F). Note that soft eggshells of derived squamates do not have stem-like structure that extends to the shell membrane (H).

Terminologies used in the text are summarized in eight schematic diagrams of different eggshells (Fig 3). The rigid gekkotan eggshells are generally composed of a covering layer, a calcareous layer (which may be ‘columnar’ or ‘plain’, or have the ‘porous layer’), and a shell membrane. The covering layer is composed of proteins [5] and occupies the outermost surface of the rigid gekkotan eggshells. The columnar layer is composed of jagged columns of calcite crystallites, which was usually considered for diagnostic character of rigid gekkotan eggshell [23]. However, the calcareous part of the eggshell is not always composed of columnar structure. Thus, a new term ‘plain layer’ is assigned to the calcareous layer without columnar structure. The *Paroedura stumpfii* eggshell has unique structure characterized by irregular pores, thus given the term ‘porous layer’. There is a blocky layer below the columnar layer in *Phelsuma grandis* eggshell but it may be also present in other unhatched rigid eggshells because *Phelsuma grandis* eggshell is the only unhatched eggshell in this study (see EDS analysis below). The shell membrane is composed of protein fibers and it occupies the inner surface of the rigid gekkotan eggshells and inner portion of the soft gekkotan eggshells.

The soft gekkotan eggshells consist of the calcareous layer containing cap-like structure and stem-like structure, the shell membrane, and the boundary layer [2,15]. The terms cap-like and stem-like structures followed [15] and they are collectively called “calcareous layer” in the main text. The boundary layer makes the innermost surface of the soft gekkotan eggshells. In *Rhacodactylus leachianus* eggshell (Fig 3F), the columnar shell unit does not exist so we used the term “mixed layer” for the main calcareous layer where proteins and calcites coexist and “crystalline layer” for an external portion where massive calcites are dominant. However, it is probable that they are homologous to the calcareous layer containing stem-like structure and cap-like structure, respectively.

## Results

We provide eggshell description of seven species of Gekkota using five different methods: microstructural images using polarized light microscope; ultrastructural SE and BSE images using FE-SEM; chemical composition description using FE-EPMA; crystallographic images using EBSD. The most important characters are provided in the main text. Refer to S1 Text for the detailed description of each eggshell by five different methods. All sections below are arranged in a consistent fashion: the common features of rigid eggshells; the unique features of each rigid eggshell; the common features of soft eggshells; the unique features of each soft eggshell.

### Microstructural features under polarized light microscope

**Fig 4:**
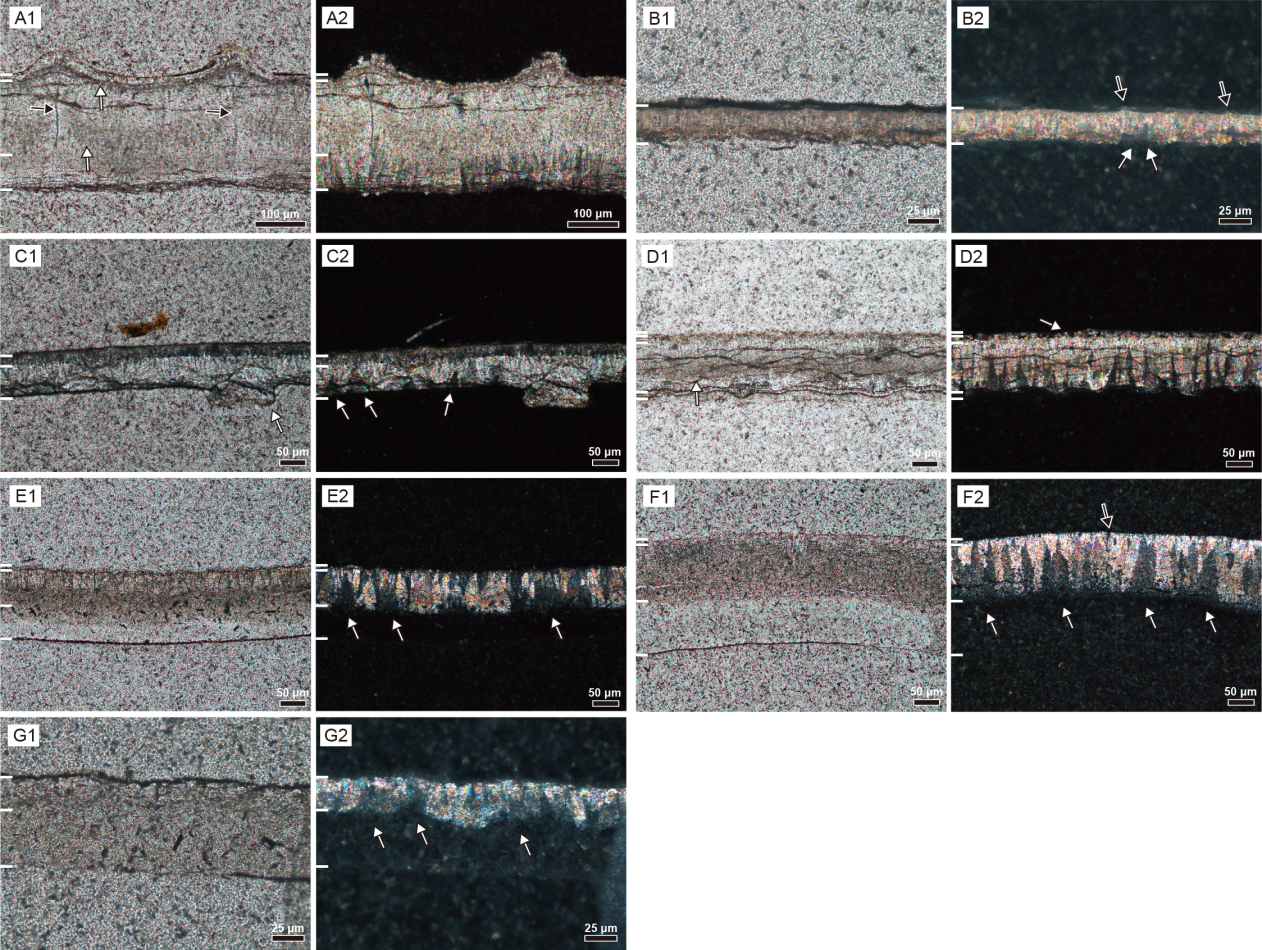
Thin section images of gekkotan eggshells. A1–G1 were taken under plane-polarized light and A2–G2 cross-polarized light. White bars on the left side of the figures represent the boundary between the layers mentioned in the text and S1 Text. Outside of eggshell is up, consistent with Fig 3. (A) *Gekko gecko*. White arrows point to the inner thick faint and outer thin dark bands, respectively. Black arrows mark the pore-like structures. (B) *Paroedura pictus*. White arrows point to the polygonal extinction patterns. Black arrows mark the location of ridge-like ornamentations. (C) *Paroedura stumpfii*. A white arrow in C1 marks a calcite concretion extending toward the interior of the eggshell. White arrows in C2 mark the triangular extinction pattern. (D) *Phelsuma grandis*. White arrows in D1 and D2 point a dark band and calcite granules, respectively. (E) *Correlophus ciliatus*. Extinction patterns are polygonal to triangular in shape (white arrows in E2). (F) *Rhacodactylus leachianus*. Extinction patterns are longitudinally thick, compared to those of *Correlophus ciliatus* eggshell (white arrows). The pore-like structure is marked by a black arrow. (G) *Eublepharis macularius*. Extinction pattern is not prominent, compared to other soft gekkotan eggshells but similar in shape (white arrows).

There are several common characters among the rigid gekkotan eggshells (Fig 4A–4D): columnar or sub-triangular to polygonal extinction patterns that become wider to the inner surface; node-like (*sensu* [2,22]) or ridge-like ornamentations; and the existence of dark band [22] in the middle of the eggshells of *Gekko gecko* and *Phelsuma grandis*. The extinction pattern reflects crystallographic orientation of calcite grains in eggshell (Fig 4A–4D; [40]; see discussion of EBSD below).

However, the microstructural features are more remarkable for their diversity than for their similarity in rigid gekkotan eggshells. Contrary to the other rigid eggshells, the columnar extinction pattern of *Gekko gecko* eggshell does not extend to the external surface. Also, it has the most conspicuous ornamentation and “pore-like” structure (*sensu* [8]; Fig 4A; S1 Fig) within it. *Paroedura pictus* eggshell is relatively homogenous with few distinct characters (Fig 4B). In *Paroedura stumpfii* eggshell, protruding calcite concretions (Fig 4C; S1 Fig) are observed in inner surface and the external portion of the eggshell is characterized by the porous layer. *Phelsuma grandis* eggshell has P-rich blocky layer (Fig 4D; see FE-EPMA analysis below), which are not conspicuous under cross-polarized light. In addition, its ornamentation is unique in that calcite granules lie on the top of the main eggshell (i.e., a surface layer in Fig 3D) and are overlain by the covering layer as *Phelsuma madagascariensis* eggshell [2].

In contrast, the microstructures of soft gekkotan eggshells are similar to one another (Fig 4E–4G). They are all composed of a shell membrane in the inner portion of the eggshell and calcareous stem-like (a loose mixture of protein and calcite) and cap-like structures. They also have similar extinction patterns as those of rigid gekkotan eggshells in that triangular to polygonal ones with same configuration were observed.

Notable differences among soft gekkotan eggshells are that *Rhacodactylus leachianus* eggshell (Fig 4F) has the deepest calcareous layer with longitudinally extended extinction pattern while *Eublepharis macularius* eggshell (Fig 4G; S1 Fig) does not have a clear extinction pattern and the cap-like structure is hardly observable. The latter phenomenon may be, however, the result of extreme thinness of the *Eublepharis macularius* eggshell.

### Ultrastructural features in SEM images

#### Secondary electron imaging

**Fig 5:**
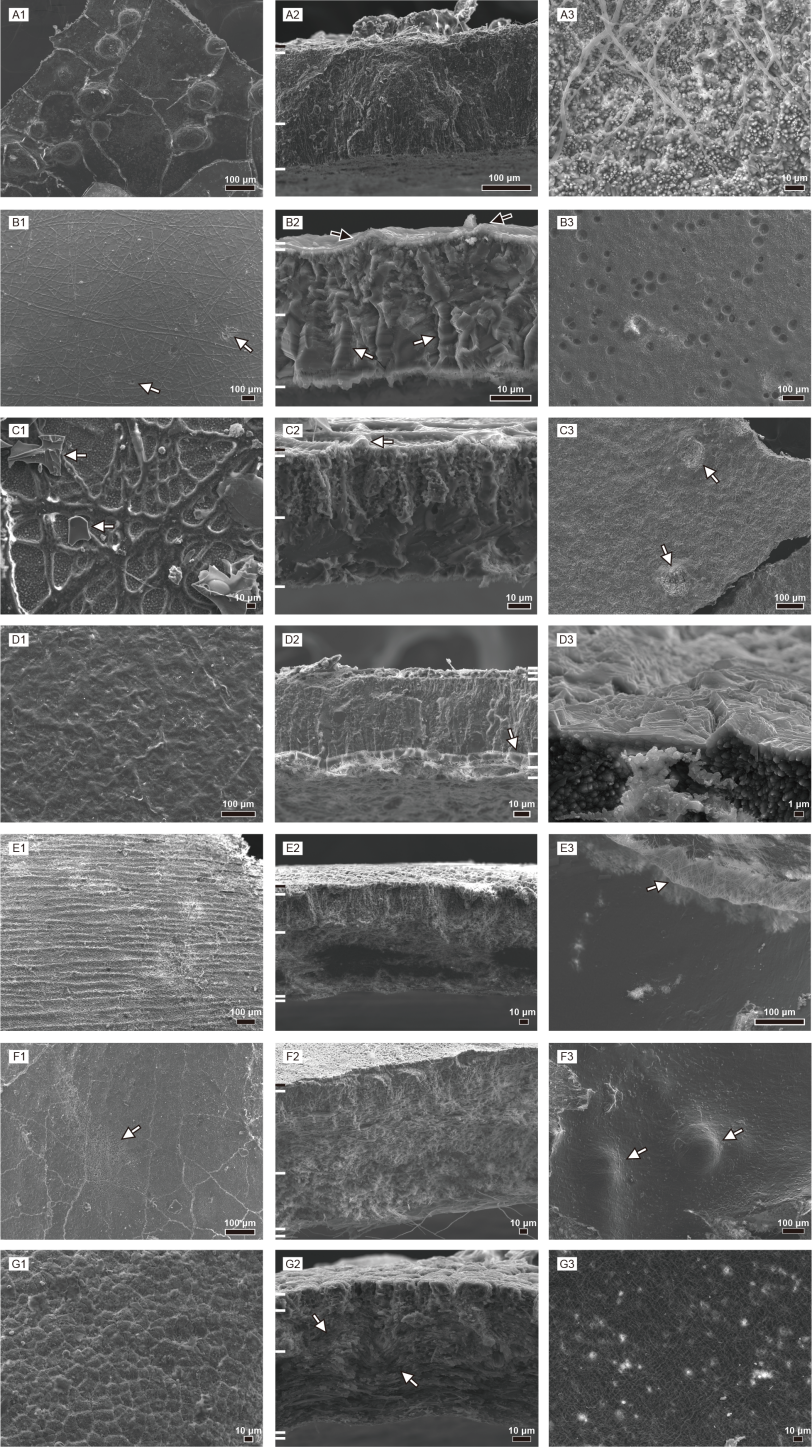
Secondary electron images of gekkotan eggshells. Each column is outer, radial, and inner views, respectively. White and black bars on the side of a central column represent the boundary between the layers mentioned in the text and S1 Text. Outside of eggshell is up. See S2–S8 Figs for details. (A) *Gekko gecko*. Ribbon-like structures in A3 are remnants of the shell membrane. (B) *Paroedura pictus*. White arrows in B1 point pore-like structures. Note columnar structure (white arrows) and ridge-like ornamentations (black arrows) in B2. The inner surface is characterized by a lot of pits. (C) *Paroedura stumpfii*. White arrows in C1 mark the fragments of sawdust so that they should be neglected. Note the absence of columnar structure in the plain layer and the presence of ornamentation (a white arrow) in C2. Protruding calcite concretions are distributed in C3 (white arrows). (D) *Phelsuma grandis*. Note that the boundary between the blocky and columnar layers in D2 is very similar to the inner surface of other rigid gekkotan eggshells where needle-like structure exists (a white arrow). (E) *Correlophus ciliatus*. The calcareous layer is weakly columnar in shape and morphologically different from the very thin calcareous layer of derived squamate eggshells. A white arrow in E3 marks the margin of the boundary layer. (F) *Rhacodactylus leachianus*. The gradual boundary between the capsule-like and flattened granules is marked in F1 (a white arrow) in the outer surface. Note the convex mounds in the boundary layer (white arrows) in F3. (G) *Eublepharis macularius*. Note that protein fibers near the calcareous layer have wave-like undulation in G2 (white arrows). The outline of eggshell is similar to those of scincid lizard eggshells [39].

Several known characters of rigid gekkotan eggshells are confirmed using secondary electron imaging: needle-like structures in the inner surface of the eggshell [8,21,22]; jagged columnar structure [22,23]; spherical shell elements (*sensu* [2]) in the outer surface; and a protein layer (= covering layer) on the outer surface [5] (Fig 5A–5D). However, it is evident that the spherical shell elements (= circular structure in BSE images below) are not only present on the outer surface, but also widely distributed in the radial section of both rigid and soft gekkotan eggshells (e.g., S3B, S4D and S8B Figs). The jagged columnar structure, a diagnostic character of rigid gekkotan eggshell [23], is composed of stacked calcite plates with intermittent horizontal fissures (S2, S3 and S5 Figs). However, in *Paroedura stumpfii* eggshell, columnar structure is not clear compared to others. Vesicles are distributed in the eggshell and are occasionally associated with the protein fibers (S2 Fig). It should be emphasized that the tips of the needle-like structure on the inner surface of rigid gekkotan eggshells (S2M–S2O, S3O, S4H–S4I and S5I Figs) are composed of stacked plate structure and their structure is completely different from the radiating mammillary layer of extant bird and non-avian maniraptoran dinosaur eggshells [41,42].

*Gekko gecko* eggshell has unique dome-shaped ornamentation (S2A–S2C Fig). In radial view, the jagged columnar layer occurs in the two-fifths of the eggshell (Figs 3A and 5A2). *Paroedura pictus* eggshell has reticular ornamentation (Fig 5B1). The most conspicuous feature of *Paroedura pictus* eggshell is its chamber-like structure in radial view (S3G–S3I Fig). It looks like a pit in inner view (Fig 5B3) and is frequently filled with calcareous material (S3M and S3N Fig) of which function is unknown. *Paroedura stumpfii* eggshell has reticulate ornamentation but it is more flattened than that of *Paroedura pictus* eggshell (Fig 5C1). In radial view, it lacks jagged columnar structure unlike other rigid gekkotan eggshell. It has also a characteristic porous layer that has vertically irregular porosity with popcorn-like calcification (S4A–S4E Fig). A few protruding calcite concretions exist in inner surface (Fig 5C3; S4G Fig). *Phelsuma grandis* eggshell also has reticulate ornamentation but it is not as clear as that of *Paroedura* eggshells (Fig 5D1). In particular, there is a blocky layer underneath the needle-like structure in the bottom of the columnar layer (S5B Fig). It might be caused by failure of embryonic development of the studying material because all other hatched rigid eggshells do not have this layer ([22]; but see S16 and S17 Figs for EDS analysis). It also has spherical calcite granules which are interwoven with fibrous proteins in the outer surface of eggshell (= surface layer) (S5D Fig).

The soft gekkotan eggshells have similar structure to one another (Fig 5E–5G). Contrary to the conventional knowledge (e.g., [2,21,43]), the calcareous layer with both stem-like and cap-like structures does not simply lie on the external surface of the shell membrane but it extends deeply into the shell membrane as tuatara eggshell [15]. The transition between the shell membrane and calcareous layer is gradual. The protein fibers and calcite grains coexist to nearly external end of the eggshell (= stem-like structure). At the external surface of the eggshell, cap-like structure without protein fibers overlies the stem-like structure. The innermost boundary layer is differentiated from the shell membrane as a layer composed of tough protein fibers.

In *Correlophus ciliatus* eggshell, there is ripple-like ornamentation in the external surface (Fig 5E1). The top of each shell unit is mushroom-like or volcano-like in shape (cap-like structure) (S6A–S6D Fig) and the shell unit itself shows a typical column configuration (stem-like structure) (S6F–S6M Fig). *Rhacodactylus leachianus* eggshell has no ornamentation on the outer surface (Fig 5F1) nor column- or wedge-like structure in a shell unit (Fig 5F2; S7F Fig). However, the calcareous layer is thicker than that of other soft eggshells. *Eublepharis macularius* has closely packed calcareous blocks on the outer surface as scincid lizard *Lampropholis* eggshell (Fig 5G1; [39]). The calcareous portion of the eggshell is the smallest among three soft eggshells. A unique character of *Eublepharis macularius* eggshell is that its fibrous proteins in the shell membrane show a complex undulating pattern (Fig 5G2).

#### Backscattered electron imaging

**Fig 6:**
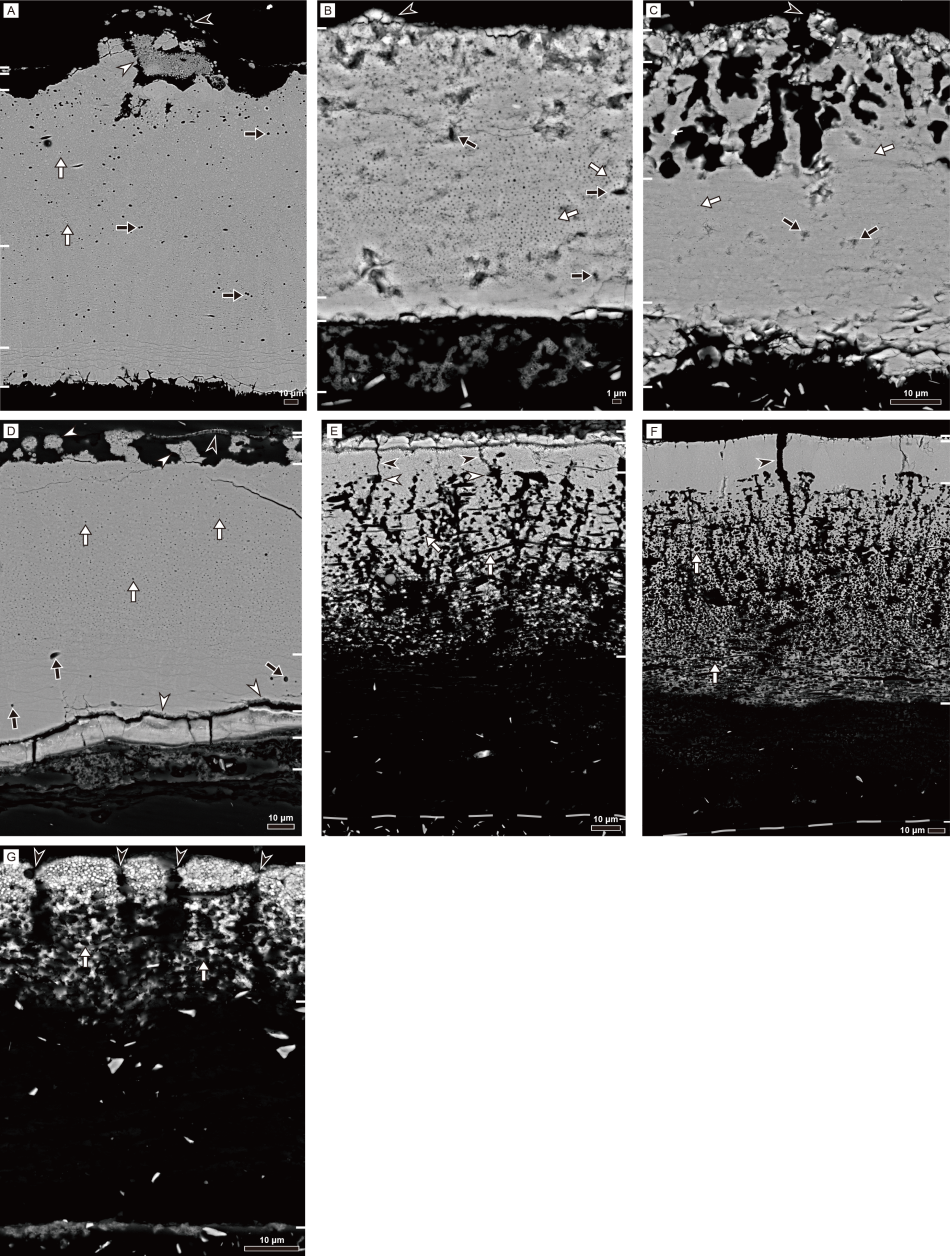
Backscattered electron (BSE) images of gekkotan eggshells. White bars on the side represent the certain boundary mentioned in the text and S1 Text. Outside of eggshell is up. See S9–S15 Figs for details. (A) *Gekko gecko*. White arrows point to the circular structures with a central hole, whereas black arrows mark the vesicles. Note the enigmatic bulbous structure in ornamentation (a white arrowhead) covered with a very thin layer consisting of polygonal structure in the outermost part of the eggshell (a black arrowhead). (B) *Paroedura pictus*. Note the circular structures with a central hole (white arrows) and abundant vesicles (black arrows). The ridge-like ornamentation is marked by a black arrowhead. (C) *Paroedura stumpfii*. Circular structures with a central hole (white arrows) are distributed in the eggshell except the inner portion of the plain layer. Vesicles are marked by black arrows. Note the highly irregular pores in the porous layer and a pore-like orifice (a black arrowhead). (D) *Phelsuma grandis*. Abundant circular structures (white arrows) and a few vesicles are present (black arrows). Note needle-like structures between the blocky and columnar layers (two lower white arrowheads). Spherical granular ornamentations are connected to the main eggshell (two upper white arrowheads) covered with a very thin covering layer (a black arrowhead). (E) *Correlophus ciliatus*. The boundary layer is marked by a dashed line. Note that calcareous layer occupies one-half of the eggshell although they coexist with protein fibers (fibers are represented by black dots and lines; white arrows). Pore-like and small chamber-like structures are pointed by black and white arrowheads, respectively. (F) *Rhacodactylus leachianus*. The boundary layer of the eggshell is marked by a dashed line. It has deeper mixed layer with a more compact outer portion than *Correlophus ciliatus* eggshell. The protein fibers are marked by white arrows. Note pore-like structure (a black arrowhead). (G) *Eublepharis macularius*. Columnar structure is clearly observable with pore-like structures between columns (black arrowheads). The stem-like structure of each columns consists of calcite and protein fibers (white arrows).

BSE images provide highly-magnified sectional images of the eggshell. In rigid gekkotan eggshells (Fig 6A–6D), the sub-parallel horizontal accretion lines are observed in the inner portion of the eggshell. Most interestingly, many tiny circular structures with a central hole are widely distributed in all rigid eggshells (S9B and S9C, S10E and S10F, S11C and S11D and S12E and S12F Figs). We think that they are the same as spherical shell elements (*sensu* [2]) that were observed on the outer surface of the eggshell. This structure has never been reported in avian and non-avian dinosaur eggshells, so that it may be a diagnostic character of gekkotan (or perhaps, squamate) eggshells. Except for *Gekko gecko* eggshell, a blocky layer (in the case of unhatched egg) or its eroded residuals were observed between the main body of eggshell and shell membrane. They may relate to the consumption of essential elements for the embryonic development (see discussion below).

In *Gekko gecko* eggshell, there is an enigmatic bulbous structure covered with the crater-like ornamentations (Fig 6A; S9D–S9I Fig). Vesicles are highly concentrated on the external end of the plain layer (S9G and S9H Fig). The sub-parallel horizontal accretion lines are bent concave up near the pore-like structure (S9I Fig) and it is consistent with the shape of a dark band in the inner portion of the plain layer (see Fig 4A). *Paroedura pictus* eggshell is characterized by the chamber-like structure (S10G and S10H Fig). The quantity of the calcareous matter inside the chamber-like structures are different among each chamber-like structure. It should be emphasized that the intact calcareous matter is usually associated with thick blocky layer whereas depleted calcareous matter lacks the blocky layer. *Paroedura stumpfii* eggshell has a unique porous layer at the external portion of the eggshell (Fig 6C). These empty spaces are not filled with fibrous proteins (S11D Fig). In *Phelsuma grandis* eggshell, a blocky layer exists between the main eggshell and shell membrane (Fig 6D; S12A–S12D Fig). The spherical calcite granules (ornamentation) in a surface layer are composed of circular structures with a central hole like the columnar layer and are usually connected to the columnar layer (S12F Fig).

The BSE image of soft gekkotan eggshells are similar to one another (Fig 6E–6G): tough protein fibers make a boundary layer in the inner end of the eggshell; shell membrane occupies at least one-third of the eggshell; a stem-like structure made up of calcite and protein fibers mixture is deeply extended into the shell membrane (*contra* [2,21,43]); spherical shell elements (*sensu* [2]) are distributed in all over the stem-like structure; and pore-like structures exist between vertical columns. However, it may possible that the pore-like structures may be cracks caused by water intake and subsequent inflation of the eggshell during incubation [8,19]. The spherical shell elements have been reported on the outer surface of the reptilian eggshells (mostly soft eggshells of lizards) [2] but this study shows that they also exist within the calcareous layer of soft gekkotan eggshells (S13B–S13D, S14B–S14E and S15B–S15E Figs). They may be homologous to the circular structures observed in the rigid gekkotan eggshells. In other words, circular structure of rigid gekkotan eggshells and spherical shell elements of soft gekkotan eggshells would have originated from the common ancestry.

*Correlophus ciliatus* and *Rhacodactylus leachianus* (both belong to family Diplodactylidae) eggshells are similar to each other. Especially, they have cap-like structure which consists of many tiny disc-shaped massive crystals (S13A–S13D and S14D–S14F Figs). On the other hand, the cap-like structure of *Eublepharis macularius* eggshell is composed of spherical shell elements (S15B–S15E Fig). There are, however, remarkable differences between diplodactylid eggshells. Compared to *Correlophus ciliatus* eggshell, *Rhacodactylus leachianus* eggshells have proportionally thicker calcareous layer, more discrete boundary between the shell membrane and calcareous layer, and prominent pore-like structures (S14A and S14B Fig). On the other hand, the shell unit of *Eublepharis macularius* is more distinctly observable than other soft eggshells due to the pore-like structures (S15B Fig). In addition, spherical shell elements of *Eublepharis macularius* which have dark spots within it become denser towards the outer surface so that they resemble the circular structures of rigid eggshells than spherical shell elements of other soft eggshells (S15D and S15E Fig).

### Chemical compositional analysis using FE-EPMA and EDS

Five elements were analyzed (Na, Mg, P, S, and Ca) using FE-EPMA but Na did not show any discernable pattern compared to background level. Hence, the result of four selected elements is presented here.

**Fig 7:**
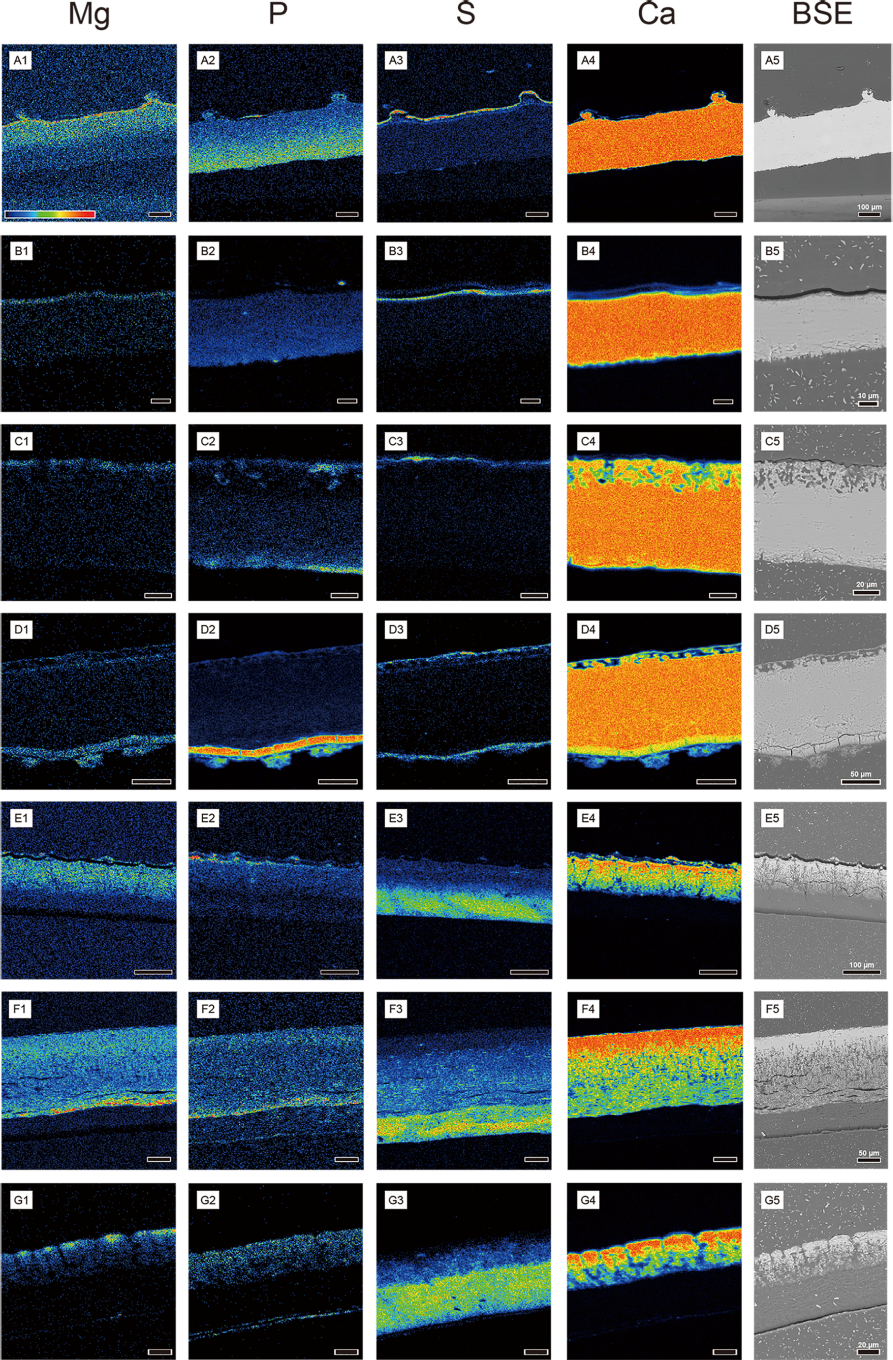
Elemental mapping images of gekkotan eggshells. The panels in the same row are on the same scale. A colored bar on A1 shows the intensity of signal with red stands for higher concentration. Outside of eggshell is up. (A) *Gekko gecko*. (B) *Paroedura pictus*. (C) *Paroedura stumpfii*. (D) *Phelsuma grandis*. (E) *Correlophus ciliatus*. (F) *Rhacodactylus leachianus*. (G) *Eublepharis macularius*.

**Fig 8:**
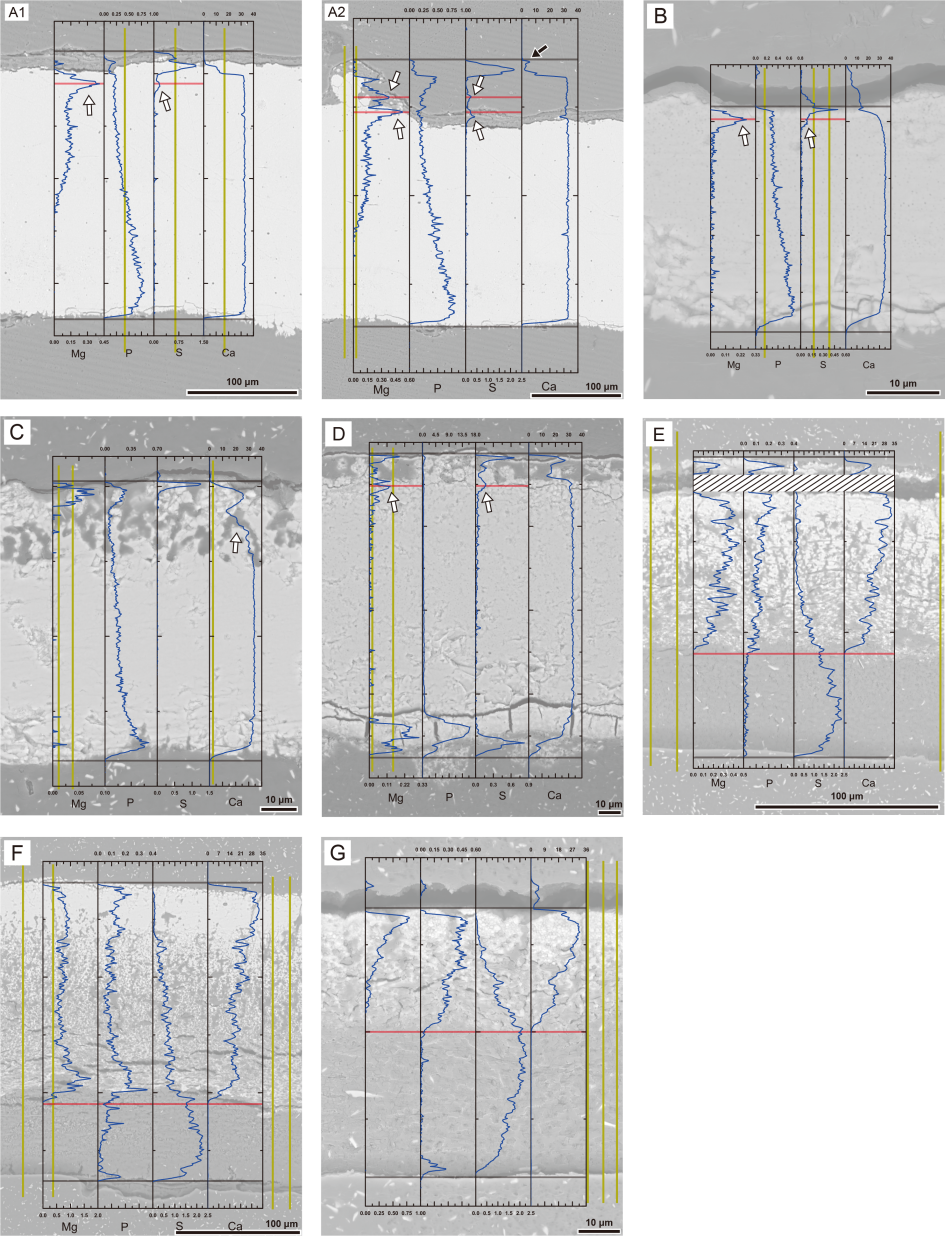
Line profile analyses of gekkotan eggshells. Vertical yellow bars represent the locations where analyses were conducted. Horizontal red bars on the graph are auxiliary lines that mark the same position in the eggshell. The boundaries of the eggshell are marked by black bars. The units are in weight percent. Outside of eggshell is up. (A1–A2) *Gekko gecko*. A1 shows the elemental profile of the main eggshell, while A2 represents the result of ornamentation area. Note the correlation between the level of Mg and S (white arrows). A black arrow points a small Ca peak caused by a very thin layer consisting of polygonal structure of the covering layer. (B) *Paroedura pictus*. Note the correlation between the level of Mg and S (white arrows). (C) *Paroedura stumpfii*. A drop of Ca level reflects the empty spaces in the porous layer rather than low concentration of Ca in the porous layer (a white arrow). (D) *Phelsuma grandis*. Note the correlation between the level of Mg and S in the main eggshell (white arrows). The level of P gradually decreases from the inner surface of the columnar layer, but it increases near the outer surface of the eggshell. (E) *Correlophus ciliatus*. The hatched rectangle marks the crack. The level of Mg, P, and Ca begins to change at the same position (i.e., the boundary between the shell membrane and calcareous layer). (F) *Rhacodactylus leachianus*. Note the level of Mg and P changes more abruptly at the boundary between the shell membrane and calcareous layer compared to *Correlophus ciliatus* eggshell. (G) *Eublepharis macularius*. Note that *Eublepharis macularius* eggshell has a relatively short calcareous layer.

The rigid gekkotan eggshells showed similar compositional profiles (Figs 7A–7D and 8A–8D). Mg was highly deposited near the outside surface of the eggshell immediately below the covering layer. The concentration of P was highest in the inner portion of the eggshell and it gradually decreased towards the outside of the eggshell. S was highly concentrated on the covering layer while a considerable quantity of S was also detected where Mg level was high. The level of Ca was highest among all the elements, showing monotonous profiles.

In *Gekko gecko* eggshell, the level of Mg appears to be higher than that of other rigid eggshells and it occurred in the outer half of the eggshell (Figs 7A1 and 8A1-2). It should be noted that there was an additional Mg peak at the bottom of the nodular ornamentation (Fig 8A2). The P concentration at the ornamentation was also characteristic that it became higher to the external end of the ornamentation. A similar phenomenon was also detected in *Paroedura stumpfii* eggshell that P concentration increased near the external end of the eggshell (Fig 8C). The profiles of *Phelsuma grandis* were unique in that exceptionally high concentration of P was detected in the blocky layer although general P behavior (increasing to the inner surface) was observed in the columnar layer (Fig 8D). However, it should be also noted that it was the only unhatched eggshell in this study. The Ca profiles of *Paroedura stumpfii* and *Phelsuma grandis* eggshells were dropped at the empty spaces of porous external end of the eggshell. Additional elemental analyses using EDS were conducted for putative residual materials of the blocky layers of hatched *Paroedura pictus* and *Paroedura stumpfii* eggshells (S16 and S17 Figs). From them, a high concentration of P (more than 4.0 wt%) was detected in both specimens. In addition, the presence of F (more than 1.5 wt%) was also detected in residual blocky layer of *Paroedura stumpfii* eggshells as well as rare occurrence of Cl. The F and Cl were also detected from the inner edge of the blocky layer of *Phelsuma grandis* eggshell (S18 Fig).

The soft gekkotan eggshells showed similar compositional profiles to one another (Figs 7E–7G and 8E–8G) but they are different from those of rigid gekkotan eggshells. In general, significant Mg and P were first detected at the boundary between the shell membrane and calcareous layer and their concentration increased towards the outer surface. Unlike the Mg profile, however, a little P was also detected in the shell membrane. The level of S was highest in the shell membrane, then diminished towards the outer surface. In the outer calcareous layer, S became nearly absent. Ca was absent in the shell membrane but appeared in the calcareous layer. The level of Ca increased to the outer surface of the eggshell, probably caused by calcite “stems”, resembling tuatara eggshells [15]. Ca profiles of soft gekkotan eggshell confirm that Ca is more deeply extended into the shell membrane than derived squamate eggshells (Fig 8E–8G). *Rhacodactylus leachianus* eggshell showed a slightly different profile compared to other soft eggshells. The levels of Mg and P were higher at the boundary between the shell membrane and calcareous layer unlike other soft eggshells (Figs 7F1–2 and 8F). Then, their levels slightly decreased to the middle portion and increased again to the outer surface of the eggshell.

### Crystallographic analysis using EBSD

**Fig 9:**
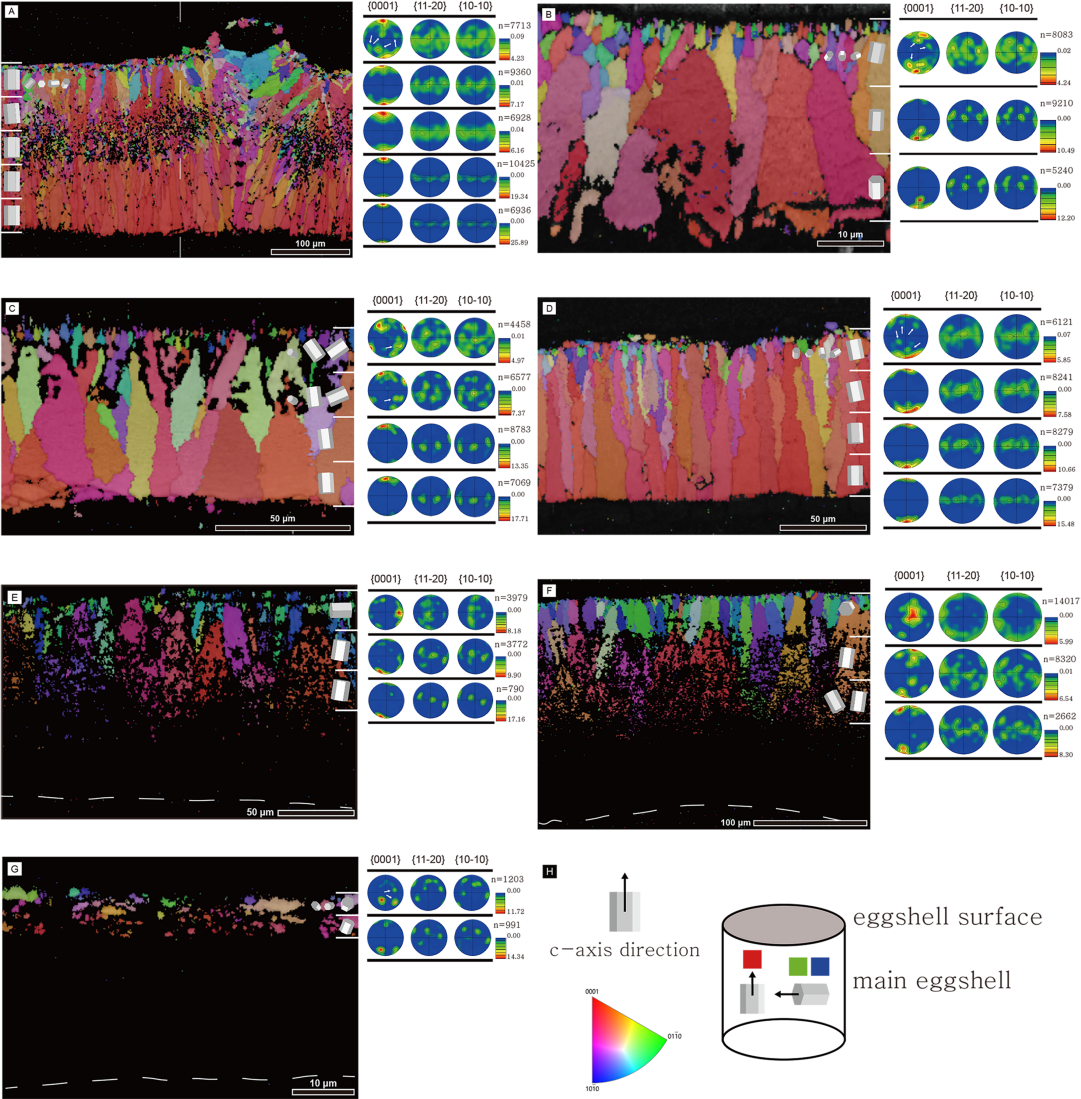
Inverse pole figure (IPF) maps and lower hemisphere pole figures of gekkotan eggshells. Each row of lower hemisphere pole figures in right columns is corresponding to the area bounded by the white bars on the IPF map. The hexagonal columns in the IPF map show the direction of c-axis orientation. The big columns represent the main direction and small columns represent the subordinate direction (i.e., correspond to a strong signal and weak signal marked by white arrows in the pole figures, respectively). Note that a- and b-axes were not considered in the construction of hexagonal columns. The numbers above the color scale represent the number of data points used in pole figure construction. The numbers on the side of the color scale are the intensity of the signal. Outside of eggshell is up. Dashed lines indicate the boundary layer (E–G). (A) *Gekko gecko*. Note that lower hemisphere pole figures were constructed only for the area to the left of a dashed line in order to avoid any disturbance caused by ornamentation and pore-like structure in the right area. (B) *Paroedura pictus*. (C) *Paroedura stumpfii*. (D) *Phelsuma grandis*. (E) *Correlophus ciliatus*. (F) *Rhacodactylus leachianus*. (G) *Eublepharis macularius*. (H) An arrow in hexagonal column points to the direction of c-axis (upper left). An IPF legend shows the relationship between the color and c-axis orientation (lower left). A cylinder on the right shows c-axis orientation schematically. Red-colored parts of the IPF map show the region where c-axis of calcite crystal is aligned perpendicular to the eggshell surface. In contrast, blue- and green-colored parts are equivalent to the calcite crystals that have horizontally aligned c-axis (i.e., parallel to the eggshell surface).

In general, all rigid gekkotan eggshells showed the same crystallographic framework: fine and randomly oriented calcite grains were distributed in the external end of the eggshell whereas large fan-shaped and vertically aligned calcite grains were present in the main part of the eggshell (Fig 9A–9D). The intensity of alignment became stronger towards the inner surface of the eggshell. *Gekko gecko* eggshell had several unique characters among rigid eggshells: the presence of “middle layer” with very fine and randomly oriented grains (S19A Fig); the odd crystallographic alignment of calcites surrounding a pore-like structure under the nodular ornamentation. The position of “middle layer” is well-matched with a faint dark band seen in the microstructural thin section image (Fig 4A).

The soft gekkotan eggshells also had similar crystallographic arrangement as rigid eggshells (Fig 9E–9G). The c-axis of the calcite grains in the external portion of the eggshell (including cap-like structure and outer part of the stem-like structure) were horizontally aligned whereas those in the internal part of the stem-like structure were perpendicular to the eggshell surface. In addition, the grains in inner stem-like structure became larger towards the inner surface. This crystallographic arrangement is very similar to that of rigid gekkotan eggshells, which is well matched with the growth mechanism proposed for the tuatara eggshell [15].

## Discussion

### Morphological similarities between soft eggshells of Gekkota and Tuatara

It is notable that the soft gekkotan eggshells have morphological similarity with the eggshell of tuatara. The soft eggshell of the tuatara consists of a shell membrane and a calcareous layer (Fig 2B). The inner part of the calcareous layer (= stem-like structure) is loosely organized with intertangled protein fibers. The stem-like structure is deeply extended into the shell membrane with its apex covered by a cap-like or cup-like structure [8,15]. This pattern is also observed in eggshells of derived squamates such as *Amphibolurus barbatus* (Agamidae) and *Varanus gouldii* (Varanidae) [8]. On the other hand, soft eggshells laid by most squamates have a thin calcareous layer on the thick shell membrane without a calcareous column (Fig 3H; [2,8]). It was known that the eggshell structure of *Eublepharis macularius* is also similar to that of derived squamates [2,21]. However, this study clearly showed that the eggshell of *Eublepharis macularius* has a loose calcareous column (= stem-like structure) below a dense part of calcareous layer (= cap-like structure) like a tuatara eggshell (Figs 4G2, 5G2, 6G, 7G4 and 8G). In addition, the same arrangement of calcite and proteins is also observed in eggshells of *Correlophus ciliatus* (Figs 4E2, 5E2, 6E, 7E4 and 8E) and *Rhacodactylus leachianus* (Figs 4F2, 5F2, 6F, 7F4 and 8F) (although *Rhacodactylus leachianus* eggshell does not have a columnar structure). Hence, soft gekkotan eggshells are morphologically more similar to those of the tuatara than derived squamates (*contra* [2,21,43]).

### The absence of a true mammillary layer of Gekkota eggshells supported by the spatial variation of Mg and P

The Mg profile of rigid gekkotan eggshells confirms that a true mammillary layer (at least avian-like) does not exist in rigid gekkotan eggshells (Fig 8A–8D). In avian eggshells, Mg concentration begins to decrease from a certain concentration at the nucleation site (= crystallites originating center) located in the inner end of the eggshell and reaches its minimum at the outer margin of the mammillary layer. After that, it shows gradual recovery to the outer end of the eggshell or maintains its concentration [12,24,25,35,38]. This pattern is absent in rigid gekkotan eggshells, indicating that the inner surface of the rigid gekkotan eggshell is chemically different from the mammillary layer of avian eggshells. Moreover, P element is enriched in the inner surface of rigid gekkotan eggshells (Fig 8A–8D), while it is minimal in the mammillary layer of chicken [24], ostrich, and emu eggshells [25]. These two lines of evidence support that rigid gekkotan eggshells do not have a true mammillary layer. Likewise, Mg and P element analyses could be used to prove the existence of the true mammillary layer in crocodile and turtle eggshells which were known to have nucleation sites in the inner end of the calcareous eggshell [2,11,44,45].

### The correlation between Mg and organic matter in rigid gekkotan eggshells

It has been stated that Mg is associated with organic matter in avian eggshells [12,24,35]. Among the elements analyzed in this study, S can be also used as a proxy of organic matter content. It is a component of two amino acids (i.e., cysteine and methionine), which have a powerful redox chemistry [46], and sulfated polysaccharides that are mainly associated with mineralized layers in avian eggshell [25]. Thus, it is a biologically crucial component [46] and commonly found in keratin associated proteins, especially in cysteine [47,48]. Moreover, cysteine and methionine are present in soft and rigid gekkotan eggshells [16].

The association between sulfur and protein is also apparent from this study (Figs 7E–7G and 8E–8G). In rigid gekkotan eggshells, the highest level of sulfur is in the covering layer where protein exists. In purely calcareous layers of *Gekko gecko*, *Paroedura pictus*, and *Phelsuma grandis* eggshells, the highest peak of sulfur is located in the outer surface just below the covering layer. The sulfur profile is well correlated with Mg profile in all rigid gekkotan eggshells (Fig 8A, 8B and 8D) except for *Paroedura stumpfii* eggshell which has a peculiar porous layer. In addition, vesicles of *Gekko gecko* eggshell are mainly distributed in the outer end of the plain layer (Fig 6A; S9 Fig). Considering the fact that vesicles reflect the position of protein fibers (S2J Fig), the distribution of vesicles in *Gekko gecko* eggshell supports that Mg is more concentrated in the protein-rich region of the eggshell (Fig 8A).

### The relationship between the dark band and the organic matter in rigid gekkotan eggshells

The dark bands exist in thin section images of some rigid gekkotan eggshells (*Gekko gecko*, *Paroedura stumpfii*, and *Phelsuma grandis*)(Fig 4A, 4C and 4D). The dark band was interpreted to be an organic-rich region in *Gekko gecko* eggshell [22]. The dark band in *Phelsuma grandis* eggshell is also well correlated with the region where many vesicles and circular structures with a central hole are present (the dark area in BSE images stands for where light elements exist, such as organic matters [36]) (Fig 6D; S12 Fig). In addition, the concentration of sulfur in *Phelsuma grandis* eggshell is slightly higher in the dark band than other regions (Fig 8D). However, no organic-related signal to the dark band region is found in *Gekko gecko* eggshell. Instead, the shape and location of a dark band in the middle portion of the eggshell (Fig 4A) is well correlated with the “middle layer” in the IPF map of the eggshell (Fig 9A; S19 Fig). It means that the “darkness” in the middle of *Gekko gecko* eggshell may be related to crystallographic features of the eggshell such as smaller grain size and/or chaotic c-axis orientation of the calcite grains rather than the presence of organic matter.

### A possibility of the functional role of rigid gekkotan eggshells as a mineral reservoir for P and F

The rigid eggshell is known as a mineral reservoir for a developing embryo [8]. For instance, diverse squamates supply embryos with calcium from eggshells [32,33]. Furthermore, our results imply that rigid eggshells also function as a reservoir for phosphorus and fluorine along with calcium. P is an essential element in bone development and growth [49] and F enhances the growth of embryonic bone [50,51]. In an unhatched eggshell of *Phelsuma grandis*, a very high concentration of P (around 15 wt%) is present in the blocky layer (Figs 7D2 and 8D). In addition, the high concentration of P (more than 5.0 wt%) is also observed in the putative residual materials of the blocky layer of hatched *Paroedura pictus* and *Paroedura stumpfii* eggshells (S16 and S17 Figs). A large quantity of F is also detected in hatched eggshells of *Paroedura stumpfii* and unhatched eggshells of *Phelsuma grandis* (S17 and S18 Figs). Considering the abnormally high P and F concentration in the blocky layer and its residuals compared to the main eggshell in unhatched and hatched eggshells, the P and F enriched layer may represent a mineral reservoir for a developing embryo. This hypothesis could be rigorously testified in future by using unhatched and hatched eggshells from the same clutch.

### The unique crystallographic feature of rigid gekkotan eggshells and its potential application in paleontology

Among all rigid eggshells of sauropsids, a unique trait of rigid gekkotan eggshells is the c-axis orientation of calcite grains. All examined archosaur eggshells have the same crystallographic structure [11]. The eggshell formation begins at the organic cores in the innermost end of the eggshell (Fig 2A). The calcareous crystals radiate externally from the organic cores and meet each other at a certain height to form the mammillary layer, then continue to grow outward to form the main body of the eggshell [8,52]. This growth pattern can be visualized in lower hemisphere pole figures made by EBSD analysis: the c-axis orientations of the mammillary layer are somewhat irregular but they become strongly aligned in the continuous layer perpendicular to the outer surface. This pattern was observed in avian [12,26,27,29,30] and non-avian dinosaur eggshells [13,26,28,31,53]. On the other hand, the c-axis orientations of rigid gekkotan eggshells are definitely different from those of archosaur eggshells. The c-axis orientations of the inner part of the eggshell are perpendicular to the eggshell surface, indicating that no true mammillary layer exists in rigid gekkotan eggshells. This upright c-axis orientation retains up to the outer surface, but the intensity of its alignment becomes weaker while it becomes stronger in dinosaur eggshells [13,31]. This crystallographic disparity between gekkotan and archosaur eggshells casts doubt on the hypothesis that all rigid sauropsid eggshells have the same mechanism of eggshell formation [8]. Moreover, if these features are autapomorphies of gekkotan eggshells, they can be used for verification of several “gecko-like” egg fossils from the Cretaceous deposits in Europe [54–59] and North America [60], the Eocene deposits in North America [23], the Oligocene deposit in Europe [2], and the Miocene deposit in Kenya [61].

### The crystallographic features of soft gekkotan eggshells and a possible homologous character of all gekkotan and tuatara eggshells

In soft gekkotan eggshells, the c-axis orientation of calcites differs considerably between the porous calcareous layer (the inner part of the stem-like structure) and dense calcareous layer (the external part of the stem-like structure and cap-like structure) (Fig 9E–9F). It lies mainly perpendicular to the egg surface in the inner porous calcareous layer while it is mainly parallel in the outer dense one. Interestingly, a similar eggshell crystallographic arrangement was reported in tuatara eggshells in the way that vertical to sub-vertical calcite growth in the inner part of the calcareous layer becomes changed by the horizontal calcite growth in the outer part as a “cap-like” region (Fig 2B; [15]). The crystallographic data of *Correlophus ciliatus* and *Rhacodactylus leachianus* eggshells imply that soft gekkotan eggshells do not only resemble tuatara eggshells morphologically but also share a similar growth mechanism. In addition, it is notable that the horizontal calcite growth in soft gekkotan eggshells is also observed near the outer surface of rigid gekkotan eggshells (Fig 9A–9D). Moreover, calcite grains in the main part of the rigid gekkotan eggshells are much larger than those of the outer surface and their c-axis orientations are perpendicular to the egg surface, which are exactly the same pattern as the calcareous layer of soft gekkotan eggshells. Thus, judging from the grain size distribution and crystallographic features of calcite grains, all gekkotan eggshells (rigid and soft) show a very similar pattern of developmental framework with tuatara eggshells. If rigid gekkotan eggshells were derived from soft eggshells as suggested [4,6], upright calcite grains of rigid gekkotan eggshells may be a homology of the calcite grains forming “stem-like structure” (*sensu* [15]) of soft gekkotan and tuatara eggshells.

### Growth direction in gekkotan eggshells may be opposite to archosaur eggshells

In archosaur eggshells, the growth begins at the organic core situated in the innermost end of the eggshell, then continues to the outer surface (Fig 2A). This pattern was demonstrated by EBSD analysis in that calcite grains in the mammillary layer (where organic cores exist) are radiating outward in the early stage of the eggshell growth while continuous layer of the late stage of eggshell growth has well-oriented c-axes and fan-shaped (arc towards the outer surface) calcite grains [12,13,26,28,29,31,53]. On the other hand, the outer part of the gekkotan eggshells is filled with small calcite grains of irregular c-axis orientations (Fig 9A–9G) while the inner layer has much bigger and well-oriented reverse fan-like (arc towards the inner surface) calcite grains without a mammillary layer. This implies that eggshell formation begins at the outer surface and grows down to the inner surface of the eggshell, which is opposite to the growth direction of archosaur eggshells if we admit that the eggshell formation mechanism of archosaurs is also applicable to the Gekkota. In fact, this hypothesis was already proposed for the eggshell formation of tuatara (Fig 2B; [15]). If we accept this hypothesis, several unique characteristics of rigid gekkotan eggshells can be understood in a logical manner. They are less well-defined shell units [5,8] and a high concentration of P on the inner surface (Fig 8) without organic cores (nucleation sites) [5,23]. The reason for less well-defined shell units and the absence of nucleation site on the inner surface are due to the absence of the mammillary layer. On the other hand, the irregular c-axis orientations are present in the outer part rather than the inner one in rigid gekkotan eggshells, implying that the nucleation site is located in the outer part (i.e., eggshell formation begins from the outside). In addition, it is known that the concentration of P increases from the mammillary layer of avian eggshell and reaches a critical concentration to terminate the eggshell formation [24,25]. In contrast, P concentration increases from the outer surface to the inner surface in rigid gekkotan eggshells (Fig 8A–8D), suggesting the eggshell formation was ended in the inner surface because P is known as a terminator in calcite (eggshell) crystallization [62]. All these evidence strongly support that the direction of gekkotan eggshell formation is opposite to that of archosaur eggshells. New research using anatomical and physiological approaches (e.g., see [63]) are necessary to understand this counterintuitive growth of gekkotan eggshells in future.

### Symplesiomorphy of basal lepidosaurid eggshells

**Fig 10:**
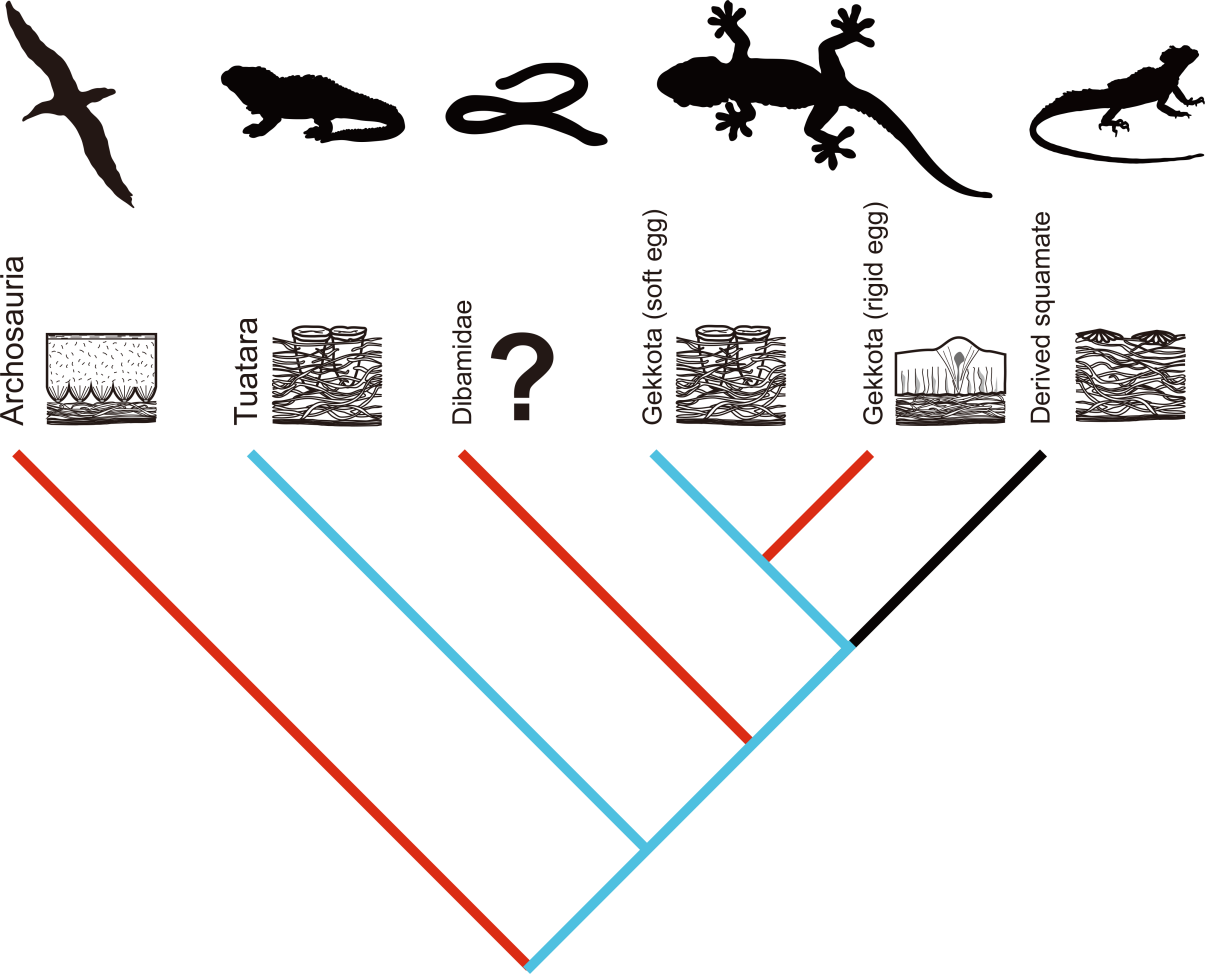
A hypothetical cladogram of rigid eggshells evolution in sauropsids. The red branches represent the rigid calcified eggshells, the blue represent the “tuatara-styled” eggshells, and the black represents the derived squamate eggshell. The archosaur eggshells are characterized by the nucleation sites at their inner margin. The tuatara and soft gekkotan eggshells are morphologically similar to each other and characterized by the “stem-like” and “cap-like” structure in the inner and outer regions, respectively. The derived squamate eggshells have a shallow calcareous layer on the surface without calcite “stem-like” structure into the shell membrane. The eggshell microstructure of the Dibamidae is unknown.

The soft eggshell of *Correlophus ciliatus* has been recently suggested as a transitional state between the universal soft-shelled eggs of squamates and rigid eggshells of Gekkota [19]. According to our study, however, the eggshell of *Correlophus ciliatus* and other soft gekkotan eggshells are more similar to tuatara eggshells rather than derived squamate eggshells (Fig 10). The tuatara-styled eggshell may be more widespread in basal squamates [8]. The Tuatara is the sister clade to the Squamata and together comprise Lepidosauria (Fig 1; [1]). The Gekkota is one of the most basal clades within the Squamata [10]. Hence, tuatara-styled eggshells would be a primitive (= symplesiomorphic) character compared to the rigid gekkotan eggshells and soft eggshells of derived squamates. In addition, crystallographic data presented above suggest that soft and rigid gekkotan eggshells have basically the same mechanism of eggshell formation, implying that they possess the same developmental pathway. If this hypothesis is true, soft gekkotan eggshells (i.e., tuatara-styled eggshells) would represent the basal character state of lepidosaurian eggshells.

Soft eggshells of derived squamates lack the loosely organized columnar structure unlike the tuatara-styled eggshell but have very thin calcareous layer (Fig 10; [2]), representing the more derived character state of lepidosaurian eggshells.

### Convergent and parallel evolution of calcified eggshells in sauropsids

Gekkotan eggshells can be used to test convergence and parallelism in the evolution of calcified eggshells in Sauropsida (Figs 1 and 10). In evolutionary biology, convergence can be defined as “independent evolution based on different developmental pathway” from distantly related clades, while parallelism is defined as “discontinuous presence of a character because of reuse of similar developmental mechanisms” between the closely related clades [64–66]. On one hand, it is evident that the rigid eggshells of the Gekkota and the Archosauria would be an example of convergent evolution, for these groups are distantly related and it is shown that their developmental mechanisms are completely different. On the other hand, rigid eggshells of the Gekkota may have a parallel evolutionary relationship with those of the Dibamidae. Excluding the Gekkota from squamates, family Dibamidae is the only clade that lays rigid eggshells [1,4]. Interestingly, the Dibamidae lies between the tuatara and Gekkota in a phylogenetic analysis [10] such that they are the most primitive clade in the Squamata (Figs 1 and 10). In addition, we can infer that the ancestor of dibamids had a soft and tuatara-styled eggshell based on the tuatara and soft gekkotan eggshells and the phylogenetic position of the Dibamidae [67].

Nevertheless, it is evident that rigid eggshells of dibamids are not a homologous character of those of the Gekkota (i.e., not derived from a common ancestry) because the eggshell rigidity of the Gekkota is phylogenetically clustered within the Gekkota (Fig 1), which means that it was generated after the cladogenesis of the Gekkota. Therefore, it is probable that rigid eggshells of the Gekkota and the Dibamidae may have similar developmental pathways due to their phylogenetic closeness and structural constraints originated from the ancestors, but this similarity is, obviously, not the result of common ancestry. To support this hypothesis, we need morphological, chemical, and crystallographic data for dibamid eggshells but, unfortunately, they were not available in this study.

Lastly, the Phu Phok eggs that were reported to small theropod dinosaur eggs from the Early Cretaceous deposit in Thailand [68] are, in fact, the only non-gekkotan squamate (anguimorph) egg fossils that have embryos *in ovo* and rigid eggshells [43]. The pattern of optical extinction pattern of these fossil anguimorph eggshells is similar to those of extant rigid gekkotan eggshells in terms of shape and configuration of calcite grains [43], implying the similarity of crystallographic characters. Assuming the similar crystallographic characters derive from a similar developmental pattern, and considering that rigid eggshells of the Anguimorpha and the Gekkota must not have been a homologous character, we further conclude that the parallel evolution for rigid eggshells already occurred in the Early Cretaceous within the Squamata based on the age of Phu Phok eggs and the skeletal and eggshell fossil records of the Gekkota [43,69].

## Supporting information

S1 Text Detailed description of each eggshell by the respective methods. (DOCX)

S1 Table Gekkotan eggshell micrographs from polarized light microscope or SEM observation in the literatures. (DOCX)

S1 Fig Additional thin section images of gekkotan eggshells. Outside of eggshell is up. (A–D) *Gekko gecko*. A white arrow in A points the pore-like structure. Note that it becomes thinner toward the inner surface. A white arrow in C shows a big concavity in the inner surface. (E–F) *Paroedura stumpfii*. A white arrow in E points a calcite concretion. White arrows in F mark the triangular or columnar extinction pattern. (G–H) *Eublepharis macularius*. Note the pore-like structures are present between the shell units (white arrows).
(TIF)

S2 Fig Additional secondary electron (SE) images of *Gekko gecko* eggshells. (A) An enlarged view of a nodular ornamentation covered with polygonal calcareous structures. (B) An enlarged view of polygonal calcareous structures. Note the depression in the central region. (C) Different types of calcareous structure in the outer surface. White arrows indicate the calcareous structure composed of minute columns. (D) Polygonal calcareous structures on the surface of the covering layer which might be in different developmental stages. (E) Starfish-like structures in the covering layer (white arrows). (F) Needle-like innermost tips of jagged columnar structure. (G) Horizontal fissures observed in the inner part of the columnar layer (white arrows). (H) A magnified view of horizontal fissures. White arrows mark the stacked calcite plates. (I) Protein fibers distributed in the eggshell. (J) An enlarged view of protein fibers. Note that the diameter of protein fiber is consistent with those of vesicles. (K–L) Protein fibers exposed in lateral view. (M–N) Acute tips of needle-like structures in inner view. Note that they can be grouped by their directions. (O) Stacked plate-like structure of the needle-like structures in high magnification.
(TIF)

S3 Fig Additional SE images of *Paroedura pictus* eggshells. (A–B) Pore-like structures. Note spherical shell elements at the external surface of the eggshell (white arrows) (C) When the covering layer is peeled off, the calcareous ridge-like ornamentation is exposed (white arrows), confirming that they are not protein fibers. (D) Needle-like tips in the inner surface of the eggshell. (E) Stacked calcite plates (white arrows) and horizontal fissure (a black arrow) in the inner columnar layer. (F) Radial view of the ridge-like ornamentation, showing that ornamentation is composed of calcites. (G–H) A chamber-like structure. Several columnar structures converge to the top of the chamber (white arrows). (I) A honeycomb-like structure in a fractured chamber-like structure (a white arrow). (J) Shell membrane interwoven with spongy calcite granules. (K–L) An enlarged view of pits in the inner surface. Note the converging columnar structure in the wall of pits and porous granules in the middle of a pit (a white arrow). (M–N) About half of the pits are filled with calcareous materials (a white arrow) and are covered with membrane-like structure which is possibly residual materials of the blocky layer (a black arrow). (O) Acute tips of needle-like structures. Note that they can be grouped by their orientations.
(TIF)

S4 Fig Additional SE images of *Paroedura stumpfii* eggshells. (A) A radial view of the eggshell. The columnar structure is absent in many different sections. (B) Stacked calcite plates (white arrows) are observed in the plain layer. Nevertheless, their orientations are irregular compared to the other rigid gekkotan eggshells. (C) An enlarged view of the porous layer. Note that the shell constituting calcite granules are apparent. (D) Ornamentation in radial view. The spherical shell elements are conspicuous at the porous layer (white arrows). (E) The covering layer is slightly separated from the main eggshell (a white arrow). (F) The shell membrane interwoven with spongy calcite granules. (G) A protruding calcite concretion is filled with needle-like structures. (H) Needle-like structures in the inner surface of the plain layer. Note the orientations of these structures. (I) An enlarged view of needle-like structures shows stacked calcite plates as *Gekko gecko* eggshell.
(TIF)

S5 Fig Additional SE images of *Phelsuma grandis* eggshells. (A) High magnification view of the outer surface of the covering layer. Minute calcite crystals are fused together. (B) An enlarged view of the inner part of the eggshell. The blocky layer is laterally continuous (a white arrow). Black arrows mark the boundary between the blocky and columnar layers. Note needle-like structures. (C) Prominent stacked calcite plates in the inner part of the columnar layer and intermittent horizontal fissures (white arrows). (D) Columnar structures are traceable to the outer surface of the eggshell (white arrows). Note that spherical calcite granules are interwoven with protein fibers (= surface layer; a black arrow). (E) When the covering layer is peeled off, the surface layer is exposed showing spherical calcite granules. (F) Innermost shell membrane interwoven with spongy calcite granules. (G) A magnified view of spongy calcite granule, which is composed of spherical subgrains. (H) This inner view shows three consecutive layers: (a) the shell membrane (outlined by black dashed line), (b) the blocky layer (outlined by blue dashed line), and (c) the inner surface of the columnar layer. The needle-like tips have different cluster orientations. (I) Needle-like structures in the inner surface of the columnar layer. Note that they are comparatively blunt than other rigid gekkotan eggshells, presumably due to the failed embryogenesis.
(TIF)

S6 Fig Additional SE images of *Correlophus ciliatus* eggshells. (A) A magnified view of suboval granules in the outer surface. (B–D) Occasionally, volcano-like convex structures make up the outer surface of the eggshell. (E) The shell membrane from outer view. (F–H) A columnar calcareous layer composed of both stem- and cap-like structures. The crystalline calcites are intertangled with protein fibers. (I–M) Columnar or wedge-like structures are present in the calcareous layer (white arrows). Note that each column is composed of cap-like and stem-like structures as tuatara eggshell [15]. (N) The boundary layer seen from the inner view. (O) The boundary layer seen from the outer view. Note that fibers in the boundary layer are more robust and tough compared to those of the shell membrane (compare (O) with (E)).
(TIF)

S7 Fig Additional SE images of *Rhacodactylus leachianus* eggshells. (A) A gradual boundary between the capsule-like and flattened granules in the outer surface. (B) An enlarged view of the capsule-like granules. (C) Enlarged view of the flattened granules. It is notable that the central concavity of the granules is similar to “cup-like central depression” of tuatara eggshell [15]. (D) The shell membrane and boundary layer. (E) Calcareous outer part of the eggshell (Mixed and crystalline layers). Note the coexistence of calcites and protein fibers. (F) The outer part of the mixed layer where few protein fiber exists. Note the absence of columnar or wedge-like structure. (G) An enlarged view of the outermost crystalline layer (= granules in the outer surface). (H) The boundary layer from inner view. Protein fibers are more conspicuous than those of *Correlophus ciliatus* eggshell. Elongated globular structures may be microbes (white arrows). (I) Thick and tough protein fibers constituting the boundary layer is observed from the inner view.
(TIF)

S8 Fig Additional SE images of *Eublepharis macularius* eggshells. (A) Closely packed calcareous blocks in the outer surface. White arrows mark the central depressions. (B) An enlarged view of a calcareous block composed of spherical shell elements (white arrows). (C) The shell membrane and boundary layers. A white arrow marks the boundary layer. (D–F) The outer part of the shell membrane and the calcareous layer composed of stem- and cap-like structures. Note that few protein fibers exist in the cap-like structure. (G) The gaps between the calcite blocks make the pore-like structure (white arrows). (H) A calcite block that has wedge-like “stem” in the lower part. Note the association between the “stem” and the wave-like pattern of protein fibers. (I) Relatively thick and tough protein fibers (compared to those of shell membrane) exist in the boundary layer.
(TIF)

S9 Fig Additional backscattered electron (BSE) images of *Gekko gecko* eggshells. (A) An enlarged view of sub-parallel horizontal accretion lines. Note needle-like structures (white arrows). (B) An enlarged view of the outer margin of the eggshell. The circular structures are well-developed near the outer surface. (C) The typical circular structure with a central hole (white arrows). (D–E) Detailed view of ornamentations. A bulbous structure within the ornamentation is marked by a white arrow. The polygonal calcareous structures above the ornamentation are marked by a black arrow. Note that the gap between the ornamentation and linear polygonal grains is filled with proteins (= the covering layer) which is invisible in BSE images. (F) An enlarged view of bulbous structures. (G–H) Vesicles are often highly concentrated near the ornamentations (bounded by dashed lines) and the outer surface. The arrows point the same structure as in D–E. (I) Note that accretion lines are bent near the pore-like structure (dashed lines).
(TIF)

S10 Fig Additional BSE images of *Paroedura pictus* eggshells. (A) An enlarged view of spongy calcite granules in the shell membrane. (B–C) Laterally continuous blocky layer is present but merely observed in this sample (white arrows). (D) The inner end of the columnar layer shows the needle-like structures (white arrows). (E–F) Abundant circular structures with a central hole. This structure is more clearly seen in the outer region of the columnar layer. (G–H) Radial view of the chamber-like structures. Some of the chamber-like structures show shrunken calcareous matters without the blocky layer (H) whereas unaffected calcareous material is covered with the well-developed blocky layer (an arrow in G). (I) A ridge-like ornamentation. A covering layer is slightly separated from the columnar layer (white arrows).
(TIF)

S11 Fig Additional BSE images of *Paroedura stumpfii* eggshells. (A) The inner part of the plain layer. Note the absence of circular structures in this part (bounded by dashed lines). (B) An enlarged view of the porous layer which is highly irregular in shape. (C–D) The circular structures are well-developed in the plain layer, while those of the porous layer are weakly-developed. (E–F) The residual blocky layer is rarely observed beneath the main eggshell (white arrows).
(TIF)

S12 Fig Additional BSE images of *Phelsuma grandis* eggshells. (A–C) An enlarged view of the inner region of eggshell. Note needle-like structures (white arrows in A). The circular structure is absent in the inner one-fourth of the columnar layer (bounded by a dashed line in A). (D) The columnar and blocky layers are interlocked by needle-like structure of the columnar layer. (E) Detailed view of circular structures. This structure is highly concentrated in the middle of the columnar layer. (F) Bud-like ornamentation composed of circular structures.
(TIF)

S13 Fig Additional BSE and SE images of *Correlophus ciliatus* eggshells. (A–C) Magnified views of the outer calcareous layer. Note pore-like structures (a white arrow) and concomitant chamber-like structures (a black arrow). (D) An enlarged view of the boundary between the stem-like and cap-like structures (a white arrow). Note that stem-like structure is composed of granular calcites whereas the cap-like structure is massive. (E–F) Radial view of the whole eggshell in SE (E) and BSE (F) images. The boundary layer of the eggshell is marked by a dashed line in (F). It confirms the existence of calcites to the middle of the *Correlophus ciliatus* eggshell. (G–I) Radial view of the calcareous layer of eggshell in SE (G) and BSE (H–I) images. Note that high porosity in the calcareous layer is caused by the presence of protein fibers.
(TIF)

S14 Fig Additional BSE and SE images of *Rhacodactylus leachianus* eggshells. (A) Pore-like structures are regularly distributed in the outer calcareous layer (white arrows). (B–C) Pore-like (white arrows) and associated chamber-like structures (a black arrow). (D) A boundary between the crystalline and mixed layers (a white arrow). Note that outermost crystalline layer is massive. (E) Detailed view of the outer mixed layer. Note granular calcites. (F) An enlarged view of the crystalline layer. Note that some of them have a central hole. (G) Most of the calcareous layer contain abundant protein fibers (dark portion) and calcites. (H–K) Radial view of the whole eggshell in SE (H, J) and corresponding BSE (I, K) images. The boundary layer of the eggshell is marked by a dashed line in I and K. Calcites exist to the middle of the eggshell. (L) An enlarged view of the outer compact calcareous layer. Note that wedge- or column-like structure is absent.
(TIF)

S15 Fig Additional BSE and SE images of *Eublepharis macularius* eggshells. (A) The wave-like pattern in the lower part of a stem-like structure is clearly seen in low magnification. The eggshell thickness is marked by two white bars on the left. (B–E) Detailed view of the dense cap-like structure. D and E show well-polished surfaces so that sections of calcite granules are more clearly exposed. Note holes in the calcite granules. (F) The stem-like structure is a mixture of calcites and protein fibers. Note protein fibers (white arrows). (G–H) White arrows in G and H point stem-like structures. Also, note the wave-like undulation of protein fibers below the wedge-like shell unit. (I) Another “stem” of the column shows their close association with protein fibers.
(TIF)

S16 Fig EDS data of *Paroedura pictus* eggshells. Note the high weight percentage of P in the residual materials of the blocky layer.
(PDF)

S17 Fig EDS data of *Paroedura stumpfii* eggshells. Note the high weight percentage of P and the presence of F and Cl in the residual materials of the blocky layer.
(PDF)

S18 Fig EDS data of *Phelsuma grandis* eggshells. Note the high weight percentage of P and the presence of F and Cl in the blocky layer.
(PDF)

S19 Fig Additional inverse pole figure (IPF) maps of rigid gekkotan eggshells. The colors represent the same crystallographic information as in Fig 9. The hexagonal columns in the IPF map show the main direction of c-axis. Note that a- and b-axes were not considered. Outside of eggshell is up. (A) *Gekko gecko*. Note the small and randomly oriented grains in the middle layer. (B) *Paroedura pictus*. The EBSD analysis was conducted on the chamber-like structure. The result showed that the columns in the chamber-like structure converge to the top of the chamber which is different from the typical c-axis direction of calcite grains in the main eggshell.
(TIF)
