# Supplementary material for: A comparative study of eggshells of Gekkota with morphological, chemical compositional and crystallographic approaches and its evolutionary implications
Source: PLoS One. 2018 Jun 22;13(6):e0199496. doi: 10.1371/journal.pone.0199496 (PMC6014675; doi:10.1371/journal.pone.0199496)
Supplement: S1 Text — (DOCX) [file pone.0199496.s001.docx]

Supplementary Information

A comparative study of eggshells of Gekkota with morphological, chemical compositional and crystallographic approaches and its evolutionary implications

Seung Choi^1, *^, Seokyoung Han^1^, Noe-Heon Kim^1^, Yuong-Nam Lee^1, *^

^1^ School of Earth and Environmental Sciences, Seoul National University, Seoul, 08826, South Korea

**Supplementary Text S1. Detailed description of each eggshell by the respective methods.**

**Microstructural features under polarized light microscope**

*Gekko gecko*

The eggshell is mainly composed of four layers: the innermost shell membrane, the columnar layer composed of the jagged columnar structure, the plain layer, and the outermost covering layer (Figs 3A, 4A). The columnar layer shows the columnar extinction pattern under cross-polarized light. The plain layer is divided into three sublayers from inside to outside: a faint dark band, a transparent layer, and a thin dark band. This pattern is far clearer in thicker thin sections (i.e., more than 30 μm). The plain layer does not have any extinction pattern under cross-polarized light. Ornamentation is irregularly distributed on the outer surface as nodes and some nodes have an empty space. “Pore-like” (*sensu* [S1]) structures are observed in the center of the nodes, but it seems that they only reached the jagged columnar layer. The width of the pore-like structure becomes thinner towards the columnar layer (S1 Fig). The pore-like structure usually begins from the empty space of the ornamentation. Rarely, large concave area (*sensu* [Packard and Hirsch, 1989]) occurs in the inner part of the eggshell but its associated concretion reported in [S2] is not observed (S1 Fig). The thickness of the eggshell without ornamentation is 0.261–0.283 mm (0.272 mm in average) whereas it is 0.305–0.335 mm (0.322 mm in average) with ornamentation.

*Paroedura pictus*

The eggshell is homogeneous under plane-polarized light and composed of the shell membrane, the columnar layer, and the outermost covering layer (Figs 3B, 4B). Under the cross-polarized light, triangular to polygonal extinction patterns exist from the bottom to the middle of the columnar layer (Fig 4B2). The outer surface is sometimes characterized by irregular ridge-like ornamentation. The thickness of the eggshell is 0.027–0.039 mm (0.033 mm in average).

*Paroedura stumpfii*

The eggshell is composed of four layers: the shell membrane, the plain layer where no columnar structure exists, the porous layer, and the outermost covering layer (Figs 3C, 4C). Protruding calcite concretions, similar to those of *Phelsuma madagascariensis* eggshell [S2], are occasionally observed in the inner surface (S1 Fig). The extinction pattern is columnar to sub-triangular and some of them reach the outer surface. The porous layer is filled with elliptical but highly irregular pores (see also BSE image below). The thickness of eggshell is 0.079–0.090 mm (0.084 mm in average).

*Phelsuma grandis*

The eggshell can be differentiated into five layers: the shell membrane, the blocky layer, the columnar layer with a dark band in the middle, and the surface layer with granular ornamentation, and the very thin covering layer which is a mixture of proteins and calcites (Figs 3D, 4D). The blocky layer is wavy and not as bright as the columnar layer above under the cross-polarized light. The columnar layer is the main part of the eggshell. Similar to the eggshell of *Gekko gecko*, a dark band exists in the middle of the eggshell. Columnar extinction pattern is apparent under the cross-polarized light which resembles that of *Gekko gecko* eggshell. However, contrary to *Gekko gecko* eggshell, it extends beyond the middle part of the eggshell and nearly reaches the outer surface. At the surface layer, numerous calcite granules (ornamentations) are distributed as *Phelsuma madagascarensis* eggshell [2,3]. These granules are covered by a very thin covering layer (see also BSE image below). Although extinction of these granules are also observed, the exact pattern of extinction is not extractable due to their tiny size. The thickness of eggshell is 0.110–0.128 mm (0.116 mm in average).

*Correlophus ciliatus*

The eggshell is composed of four layers: the boundary layer, the shell membrane composed of proteins occupying the inner half of the eggshell, the stem-like structure which is a mixture of calcites and proteins, and the outermost cap-like structure where no protein fiber exists and compact than the stem-like structure below (Figs 3E, 4E). The boundary layer and shell membrane do not show any characteristic features under the light microscope. The boundary between the shell membrane and stem-like structure is gradational under plane-polarized light. Sub-triangular to polygonal extinction patterns are observed at the stem-like structure under cross-polarized light and some of these extinctions reach the outer surface. The cap-like structure is laterally continuous. The outer surface of the eggshell is smooth but a few bumps exist, which is a reflection of the wave-like ornamentation of surface (see Fig 5E1). The thickness of the eggshell ranges from 0.153–0.167 mm with an average of 0.160 mm. The average thickness of the calcareous layer is 0.090 mm.

*Rhacodactylus leachianus*

The eggshell is mainly composed of four layers: the boundary layer, the shell membrane, the mixed layer where calcites and proteins coexist, and the crystalline layer solely composed of calcites (Figs 3F, 4F). The boundary layer and shell membrane are not characteristic. The boundary between the shell membrane and mixed layer is abrupt, compared to *Correlophus ciliatus* eggshell. The mixed layer shows columnar or wedge-like extinction pattern which is more extended longitudinally to the bottom than that of *Correlophus ciliatus* eggshell under cross-polarized light. This extinction pattern reaches the boundary between the mixed and crystalline layers. The crystalline layer (see BSE image below) is very thin and lacks extinction pattern under cross-polarized light. The outer surface of the eggshell is mainly smooth but low relief mounds are present with pore-like structures (see also Fig 6F). These pore-like structures link the interior of the shell to the exterior, piercing the calcareous layer. The thickness of eggshell ranges from 0.216–0.229 mm with an average of 0.223 mm. The average thickness of the calcareous layer is 0.156 mm.

*Eublepharis macularius*

The eggshell is composed of four layers: the boundary layer, the shell membrane, the stem-like structure, and the cap-like structure (Figs 3G, 4G). The boundary layer and shell membrane are not characteristic under polarized light microscope. The stem-like and cap-like structures do not show sharp extinction pattern but their pattern is similar to that of *Correlophus ciliatus* eggshell in that sub-triangular to polygonal extinction is dominant. In high magnification, the outer surface of the eggshell is characterized by the wavy undulation, which is similar to those of scincid lizard eggshells [S4]. Each unit of undulation is bounded by the “pore-like” structures (S1 Fig). The thickness of the eggshell is 0.066–0.076 mm (0.071 mm in average), and the average thickness of the calcareous layer is 0.036 mm.

**Ultrastructural features in SEM images**

***Secondary Electron Image***

*Gekko gecko* (Fig 5A; S2 Fig)

The outer surface is distinguishable by its nodular ornamentation (Fig 5A1). They are dome-shaped with around 100 μm in diameter. Each ornamentation is covered with morning glory-shaped polygonal calcareous structures that have a central concavity. Not all the polygonal structures look the same but a few of them are composed of minute columns. The polygonal structures are also distributed on the surface of the covering layer. Below the polygonal structure, starfish-like structures cover the covering layer. The covering layer beneath the polygonal structures looks smooth. This layer is occasionally cracked so the underlying plain layer can be seen through the cleavage. The fresh radial section of *Gekko gecko* eggshell shows three distinct layers: the columnar layer composed of the jagged columnar structure, the outer two-thirds plain layer, and the outermost covering layer (Fig 5A2; [S2]). The innermost surface of the columnar layer is needle-like (*sensu* [S1]) and horizontal fissures are observed in the inner part of the columns. The stacked calcite plates forming a column are clearly observable in the inner region of the columnar layer. The plain layer is amorphous and not characteristic. Protein fibers are distributed in both columnar and plain layers and are randomly oriented. Where these fibers are eliminated, vesicles (*sensu* [S5]) and hollow tube-like pits are left behind. The width of protein fibers are equivalent to those of vesicles. Remnants of the shell membrane (i.e., a membrane between the eggshell and albumen) are distributed across the inner surface of the eggshell. The inner surface is filled with the acute tips of jagged columns (Fig 5A3). As pointed out by [S2], a group of tips has the same orientation. In high magnification, the stacked plate structure of the tip is prominent which is definitely different from the radiating mammillary layer of bird eggshells [S6,S7].

*Paroedura pictus* (Fig 5B; S3 Fig)

The outer surface is covered with reticular ornamentation in low magnification (Fig 5B1). Several pore-like openings exist which are round to sub-oval in shape but not all of them penetrated to the inner end. The covering layer is very thin so underlying spherical shell elements are seen through it. Where the covering layer was peeled off, minute spherical shell elements are observed. The radial section of the eggshell could be divided into three layers: the inner columnar layer where columnar structure is clearly seen, the outer columnar layer where columnar structure is weakly developed, and the thin covering layer (Fig 5B2). The inner surface of the columnar layer is needle-like. The outer columnar layer is relatively amorphous compared to the inner columnar layer but some columns are traceable to the outer surface. The outer end of the columnar layer has ridge-like ornamentation, confirming that ornamentation in the outer surface is made by the calcites, but not by protein fibers. Many vesicles are observed in the eggshell regardless of the location. Occasionally, chamber-like structure occupies the eggshell. The upper surface of this structure is composed of well-developed columnar structure that converges towards the top of the chamber. In addition, several porous fan-shaped honeycomb-like structures are observed in the chamber. The inner surface of the eggshell is covered with the shell membrane with spongy calcite granules. Where the shell membrane was peeled off, the inner surface of columnar layer was exposed to show the characteristic pits (=chamber-like structure) that vary in diameter (Fig 5B3). These pits are hollow or porous granular structures are observed inside them. Sometimes, they are filled with the calcareous matter (=honeycomb-like structure?) whose function is mysterious or covered with membrane-like matter (possibly the remnants of block layer; see EDS analysis below). The general inner surface of columnar layer shows needle-like structure.

*Paroedura stumpfii* (Fig 5C; S4 Fig)

The outer surface is similar to that of *Paroedura pictus* eggshell in that ornamentation covers the main eggshell below but the ornamentations of *Paroedura stumpfii* eggshell are more flattened than those of *Paroedura pictus* eggshell (Fig 5C1). Its radial section is fairly different from those of other rigid gekkotan eggshells and could be divided into three layers: the inner half plain layer, the outer half porous layer, and the outermost covering layer (Fig 5C2). The plain layer does not have the columnar structure unlike other rigid gekkotan eggshells. Although stacked calcite plates are seen from this layer, they do not show a unidirectional pattern but somewhat random orientation. In contrast, the outer half of the eggshell (=porous layer) is highly porous and composed of spherical shell elements. The porous layer is filled with highly irregular pores. The outer surface of the eggshell is generally flat but ornamentations are distributed as in *Paroedura pictus* eggshell. The covering layer is slightly separated from the main eggshell. The inner surface is covered with the shell membrane with spongy calcite granules that cover needle-like structures in inner view. A few protruding calcite concretions are observed on the inner surface (Fig 5C3). The tips of needle-like structure resemble those of other rigid gekkotan eggshells.

*Phelsuma grandis* (Fig 5D; S5 Fig)

In low magnification, the outer surface shows reticular ornamentation but not as clear as those of *Paroedura* eggshells (Fig 5D1). In high magnification, minute polygonal calcite crystals (no more than 1 μm^2^ in area) are observable on the surface which are fused together to form a smooth surface texture. The radial section is mainly composed of five layers: the innermost shell membrane with loose calcite granules, the blocky layer, the jagged columnar layer which is a main part of the eggshell, the surface layer where calcite granules and protein are interwoven, and the very thin covering layer (Fig 5D2). The shell membrane is mostly composed of protein fibers and spongy calcite granules. The blocky layer is morphologically different from the columnar layer above in that it lacks any structural characters. The tip of the jagged columnar layer is needle-like and resemble the inner surface of other rigid gekkotan eggshells. Occasionally, horizontal fissures and stacked calcite plates that constitute a column are observed near the inner part of the columnar layer as in *Gekko gecko* and *Paroedura pictus* eggshells. The jagged columnar layer is the main part of the eggshell. Contrary to the eggshell of *Gekko gecko*, however, these columnar structures reach the outer surface as in the case of *Phelsuma madagascarensis* eggshell [S2]. Above the columnar layer, a surface layer exists where spherical calcite granules are interwoven with fibrous proteins [S3]. Finally, the covering layer covers the outermost end of the eggshell. The shell membrane is composed of loose calcite granules embedded in the protein matrix in inner view. These oval granules consist of tiny platy particles. Where the shell membrane was peeled off, the exposed inner surface of blocky layer shows platy units. Where the blocky layer was eliminated further, needle-like structure is observed (Fig 5D3) with protein fibers paralleled to the inner surface. However, they are not as acute as those of other rigid gekkotan eggshells, which might be related to failed embryogenesis of the material [S2].

*Correlophus ciliatus* (Fig 5E; S6 Fig)

The outer surface has conspicuous ripple-like ornamentation (Fig 5E1). Ridges are parallel and the wavelength between the ridges is approximately 57 μm. The outer surface is composed of suboval granules and many of them had a low relief depression at the central region. In some places, however, each granule has a volcano-like convex structure with a pinhole in the center. It seems that this convex structure represents the top layer of each columnar shell units. The radial section of the eggshell could be divided into four layers: the innermost boundary layer composed of very compact protein fibers, the shell membrane, the stem-like structure that have columnar or wedge-like shapes, and the cap-like structure which is composed of suboval granules (Fig 5E2). The inner half of the eggshell is solely composed of the shell membrane. The stem-like structure gradually begins from the shell membrane. There are numerous protein fibers in the stem-like structure but they are not as much as the underlying shell membrane. The upper portion of the stem-like structure is composed of dense calcites with the nearly complete absence of protein fibers. The columnar or wedge-like shell units (which are composed of both stem- and cap-like structures) are approximately 35 μm tall, and structurally very similar to tuatara eggshell [S8]. The boundary layer is composed of rigid protein fibers and morphologically different from the shell membrane lying above (Fig 5E3).

*Rhacodactylus leachianus* (Fig 5F; S7 Fig)

The outer surface is featureless in low magnification (Fig 5F1). It has a smooth surface without ornamentation. In high magnification, the minute granular constituents are observed on the outer surface. Except for capsule-like structure in some areas, they are flattened with a concave central region as in the case of *Correlophus ciliatus* eggshell. The granular structures are also fused together to form a flattened surface. The radial section has a similar structure with that of *Correlophus ciliatus* eggshell. It could be divided into four layers: the innermost boundary layer composed of compact fibers, the shell membrane, the mixed layer of calcites and proteins without columnar or wedge-like structure, and the outermost crystalline layer composed of very thin minute granules (Fig 5F2). The shell membrane of the eggshell is composed of proteins. Unlike other soft gekkotan eggshells, the mixed layer that may be homologous to stem-like structure of other soft eggshells does not have columnar or wedge-like structure. The outer portion of mixed layer and crystalline layer are solely composed of calcite and protein fibers do not exist. The inner surface of the eggshell (=boundary layer) is composed of rigid protein fibers (Fig 5F3). Rarely, convex mounds are observed where protein fibers are dominant. Compared to *Correlophus ciliatus* eggshell, protein fibers are more conspicuous in *Rhacodactylus leachianus* eggshell. Many elongated globular structures are observed but it is not certain whether they are microbes or eggshell components.

*Eublepharis macularius* (Fig 5G; S8 Fig)

The outer surface is composed of closely packed calcareous blocks as that of a scincid lizard *Lampropholis* eggshell (Fig 5G1; [S4]). These blocks composed of spherical shell elements are sub-angular to sub-round in shape. In many cases, central depressions exist on the blocks which resemble those of tuatara eggshells [S8]. The eggshell could be divided into four layers in radial view: the innermost boundary layer, the shell membrane, the stem-like structure, and the outermost cap-like structure (Fig 5G2). The eggshell of *Eublepharis macularius* has a deep shell membrane that occupies most of the eggshell thickness [S3,S9]. In addition, protein fibers near the calcareous layer show wave-like pattern. Contrary to the conventional knowledge that the thin calcareous layer simply overlies the shell membrane below (e.g., [S3]), calcite shell units are composed of stem- and cap-like structures as *Correlophus ciliatus* and tuatara eggshells. The outer surface also shows wave-like outline as scincid lizard eggshells in radial view due to the calcite blocks and their convexity [S4]. The boundary layer does not look very tough compared to other soft gekkotan eggshells so that fibrous proteins in the shell membrane are seen through the boundary layer (Fig 5G3).

***Backscattered Electron Image***

*Gekko gecko* (Fig 6A; S9 Fig)

The inner half of the eggshell is characterized by the sub-parallel horizontal accretion lines, which may be horizontal fissures mentioned in Secondary Electron image. They are highly conspicuous in the innermost part of the columnar layer (around one-sixth of the whole eggshell), but they abruptly weaken and gradually fade out at the middle of the eggshell. Circular structures with a central hole appear where accretion lines become weak. A central hole in the circular structures is much smaller than the vesicles in the eggshell. The plain layer is filled with circular structures that become larger at the outer surface. Contrary to the circular structures in the columnar layer, many of them in the plain layer do not have a central hole. The ornamentation is crater-shaped and its cavity is filled with an enigmatic bulbous structure. The tiny polygonal structure at covering layer is observable. The distribution of the vesicles is noticeable in BSE image. Many of them are circular to sub-circular (tube-like shapes are just a reflection of the random orientation of protein fibers). The density of vesicles is usually higher near the outer surface than any other regions. Near the pore-like structure, the curve of horizontal accretion lines is concave up which is matched with the bending of dark bands near the pore-like structure (Fig 4A).

*Paroedura pictus* (Fig 6B; S10 Fig)

The spongy calcite granules are observed in the shell membrane. In rare cases, the blocky layer is present between the shell membrane and columnar layer (see EDS analysis below). Above the shell membrane (and blocky layer) is a massive calcareous sublayer without any characteristics in the inner part of the columnar layer. The main columnar layer continues to the outer surface with abundant circular structures with central holes. These structures are more densely concentrated in the inner part than the outer part of the columnar layer. The chamber-like structures contain a different quantity of calcareous matter. Usually, fully filled calcareous matter coexists with a well-preserved blocky layer (=membrane-like matter in Secondary Electron image). The outer surface of the eggshell is mostly flat except for a few irregularly distributed ornamentations. The calcite in the covering layer is slightly separated from the columnar layer below.

*Paroedura stumpfii* (Fig 6C; S11 Fig)

The BSE image can be further differentiated into four sublayers. The inner part of the plain layer (one-fourth of the eggshell) has horizontal fissures with the needle-like structure at its innermost end. In the outer part of the plain layer, circular structures with a central hole begin to appear and continue to the outer end of the eggshell. The porous layer is highly porous with vertically extended irregular pores, composed of circular structures. Although circular structures are present in the porous layer, they are less developed than those of the plain layer. No protein fibers exist in the pores of the porous layer. The outermost part of the porous layer is a continuous calcareous sublayer occasionally punctured by orifices. In very rare cases, the blocky layer is present between the shell membrane and columnar layer as *Paroedura pictus* eggshell (see EDS analysis below).

*Phelsuma grandis* (Fig 6D; S12 Fig)

The BSE image shows six different sublayers. The shell membrane is made up of porous calcite granules and protein fibers. The calcareous part of the eggshell begins by the blocky layer which is detached from the main eggshell. The inner end of the columnar layer is characterized by the needle-like structure. Around one-fourth of the columnar layer is distinguished by its sub-parallel accretion lines, which are similar to those of *Gekko gecko* eggshell. This structure extends to the middle of the columnar layer but becomes gradually weaken. The most characteristic feature of the outer three-fourth of the columnar layer is circular structures with a central hole. These structures are highly concentrated in the middle part of the columnar layer. Vesicles are also seen from the columnar layer but not as common as those of *Gekko gecko* eggshell. The shape of the circular structures become clear in the outer part of the columnar layer. The ornamentations are bud-like, containing circular structures. The outermost part of the eggshell is composed of the very thin covering layer (nearly 1 μm in width).

*Correlophus ciliatus* (Fig 6E; S13 Fig)

In BSE images, the inner half of the eggshell is occupied by the shell membrane, and the outer half is filled with stem- and cap-like structures. The stem-like structures contains loose calcites which increase towards the outer surface. At the outer part of the stem-like structure, protein fibers are nearly absent. The stem-like structure is composed of granular calcites. It is notable that the pore-like structures connect the exterior to the inner region of the eggshell. The shape of these pore-like structures is irregular but chamber-like structures are frequently observed below them; the irregular spaces in chamber-like structures are connected by narrow and complicate canals (which may be protein fibers). The calcite crystals at the cap-like structure are massive and clearly different from the granular calcites of the stem-like structure.

*Rhacodactylus leachianus* (Fig 6F; S14 Fig)

The BSE image of *Rhacodactylus leachianus* eggshell is also similar to that of *Correophus* *ciliatus* eggshell. However, the calcareous layer of the eggshell is proportionally larger than that of *Correlophus ciliatus* eggshell, and the boundary between the shell membrane and calcareous layers is more clearly defined. In addition, the outer part of the mixed layer, where no protein fiber exists, is more developed than the outer part of the stem-like structure of *Correlophus ciliatus* eggshell. The mixed layer is composed of granular calcites. The pore-like structure is wider and less complicated than that of *Correlophus ciliatus* eggshell which are distributed at approximately regular intervals. However, they may be cracks caused by the water intake and subsequent inflation of the eggshell during the incubation [S1,S10]. In most cases, pore-like structures also have variously sized chambers beneath the surface. The calcite crystals at the crystalline layer are massive as the cap-like structure of *Correlophus ciliatus* eggshell and some of them have a central dark hole.

*Eublepharis macularius* (Fig 6G; S15 Fig)

The columnar structure of the calcareous layer is confirmed in BSE images. The lower part of the calcareous layer is a mixture of protein fibers and calcites as other soft gekkotan eggshells whereas its outermost part (=cap-like structure) is completely composed of calcite granules. Several spherical shell elements of the cap-like structure have holes as other rigid gekkotan eggshells but the holes are randomly distributed rather than located in the center.

**Chemical compositional analysis using FE-EPMA and EDS**

*Gekko gecko* (Fig 7A; Fig 8A)

Mg profile showed that more Mg was concentrated on the outer half of the eggshell. The thickness of the Mg-poor region was approximately consistent with the height of the columnar layer. Mg was particularly enriched in the outer margin of the plain layer and ornamentation. In ornamentation, Mg showed double peak due to the additional Mg peak at the bottom of the ornamentation. P showed an opposite distributional pattern that it was concentrated on the inner surface and became scarce to the outer surface. However, the overall signal intensity of P was rather gradual than that of Mg and was present all over the eggshell. It was notable that the concentration of P was somewhat higher at ornamentations than the outer part of the plain layer. Abundant S was present in the covering layer. Although not as abundant as in the covering layer, considerable amount of S was present in the calcareous plain layer just below the covering layer. The ornamentations were formed above the S-rich area of the plain layer. The signal of Ca was the strongest among all elements and it did not show any characteristic pattern but it confirmed that polygonal structures at the covering layer (Figs 5A1; 6A) were composed of calcium.

*Paroedura pictus* (Fig 7B; Fig 8B)

Mg was concentrated on the outer end of the columnar layer. P had an opposite pattern as *Gekko gecko* eggshell that the highest P concentration appeared on the inner surface and it became lower as it goes to the outer surface. Level of S was the highest on the covering layer but the columnar layer just below the covering layer also had S as a component. As in *Gekko gecko* eggshell, Ca showed the strongest signal. It appeared that the Ca level of *Paroedura pictus* eggshell was gradually increased from the inner surface compared to *Gekko gecko* eggshell but it would be an artifact caused by the extreme thinness of *Paroedura pictus* eggshell.

On the other hand, additional analyses using EDS were conducted on the putative remnant blocky layer between the shell membrane and columnar layer. The result showed that the concentration on P in this layer was exceptionally high (more than 11.0 wt%) compared to the main eggshell (less than 0.8 wt%) (see S16 Fig).

*Paroedura stumpfii* (Fig 7C; Fig 8C)

Mg had the highest peak in the outer end of the porous layer slightly below the covering layer. P level was the highest on the inner surface and then gradually became lower as it goes to the outer surface. At the outer end of the porous layer, however, it increased again. S had the highest peak in the covering layer. The profile of Ca was similar to that of *Paroedura pictus* eggshell but it became low in the porous layer compared to the plain layer due to the vertical pores.

As in *Paroedura pictus* eggshell, compositional analyses using EDS also proved that high concentration of P (more than 4.0 wt%) presented in the putative blocky layer beneath the plain layer, where P did not exceed 0.70 wt%. Moreover, the presence of F was also confirmed in the putative blocky layer, whose concentration exceeded 1.5 wt% (see S17 Fig) as well as the rare occurrence of Cl. F was absent in the main eggshell.

*Phelsuma grandis* (Fig 7D; Fig 8D)

Mg was highly concentrated on the blocky layer, the outer part of the columnar layer, and the surface layer or covering layer. P had an exceptionally high concentration in the blocky layer and porous calcite granules in the shell membrane. Although it was hard to see the delicate changes of P concentration in the columnar layer due to the exceptionally high concentration of P in the blocky layer, the concentration of P became gradually lower as it goes to the outer surface in the columnar layer as other rigid eggshells. In the outer end of the columnar layer and the surface layer, however, it became high again. S was highly concentrated on the both shell membrane and covering layer. It was notable that the level of S became higher in the outermost part of the columnar layer as *Gekko gecko* eggshell. The profile of Ca initiated from the porous calcite granules in the shell membrane and continued to the outer surface of the columnar layer. As the case of *Paroedura stumpfii* eggshell, a drop of Ca concentration at the surface layer reflects the region where calcareous matters do not exist.

Additional compositional analyses using EDS were conducted on the inner edge of the blocky layer. The result showed that F and Cl are present along with other trace elements such as Si (see S18 Fig).

*Correlophus ciliatus* (Fig 7E; Fig 8E)

The outer portion of the eggshell was cracked during the experiment. Accordingly, the data of cracked region should be disregarded, and the remnants of the eggshell above the crack are the continuation of the main eggshell.

The intensity of Mg gradually increased from the inner end of the calcareous layer and became stronger as it goes to the outer surface. Contrary to rigid gekkotan eggshells, P level of *Correlophus ciliatus* became higher as it goes to the outer surface, especially near the ornamentations. S was highly concentrated on the shell membrane of the eggshell and was poorly present in the calcareous layer but the transition was gradual. Ca signal gradually increased from the boundary between the shell membrane and calcareous layer, reflecting the proportion of calcites and proteins in the stem-like structure. Along with BSE images, this result confirmed that Ca was not just present in the outer margin of the eggshell like a thin covering as derived squamate eggshells but it already existed from the middle of the eggshell.

*Rhacodactylus leachianus* (Fig 7F; Fig 8F)

Mg signal was distinctive in that it had a high concentration at the inner end of the calcareous layer, then diminished in the middle of it, and finally, gradually increased near the outer end of the mixed layer. The boundary between the shell membrane and mixed layer had the highest Mg intensity. The signal of P was concordant with that of Mg. P was intensively deposited in the boundary between the two layers. After its decline in the middle of the calcareous layer, it weakly increased near the outer end of the mixed layer. Contrary to the case of Mg, P was also present in the shell membrane like other soft gekkotan eggshells and had a higher peak in the boundary layer. The pattern of S resembled that of *Correlophus ciliatus* eggshell in that most of S was concentrated in the shell membrane. Unlike *Correlophus ciliatus* eggshell, however, S concentration was rather higher in the middle of the calcareous layer, then it dropped to a lower level in the outer end of the mixed layer. Like *Correlophus ciliatus* eggshell, there was no Ca in the shell membrane. Ca began to be detected from the boundary or the two layers and its concentration retained to the outer one-fourth point of the calcareous layer, and at the outer part of the mixed layer where a dense calcite layer developed, it reached the highest level. Ca profile also confirmed the existence of the carbonate “stem” in the middle of the eggshell.

*Eublepharis macularius* (Fig 7G; Fig 8G)

Mg level increased from the “stem” of the calcareous layer and reached its highest peak in the outer surface. P was detected and marked with high level of concentration in the boundary layer as *Rhacodactylus leachianus* eggshell*.* It increased from the boundary between the shell membrane and calcareous layer. The level of P increased to the outer surface and reached its highest peak in there. The signal of S was the highest in the shell membrane, then at the calcareous layer, it gradually dropped. The Ca profile began in the middle of the eggshell and gradually increased as other soft gekkotan eggshells. The existence of calcite “stem” was also confirmed.

**Crystallographic analysis using EBSD**

*Gekko gecko* (Fig 9A; S19 Fig)

Compared to other rigid gekkotan eggshells, the eggshell of *Gekko gecko* showed several unique crystallographic characters: the inner part of the plain layer was characterized by very fine and randomly aligned grains; the different calcite arrangement at the ornamentation with pore-like structure. The grain size of *Gekko gecko* eggshell varied: the outer surface had fine grains but they gradually became taller and wider as they go to the inner surface. However, smaller grains appeared again in the middle of the eggshell (see S19 Fig). The c-axis orientations of calcites in the outer surface were irregular but those below were vertically oriented. The smaller grains in the inner part of the plain layer showed highly erratic orientation. After that, the “uprightness” of the calcite became stronger as it goes to the inner surface of the eggshell. The IPF map also showed the unique calcite orientations near the pore-like structure and ornamentation. The grains near the ornamentation were bigger and preferred horizontal c-axis orientation than those of other surface areas. The “middle layer” where tiny randomly-oriented grains exist was lifted near the pore-like structure (see also faint dark band in Fig 4A1), implying that this “middle layer” is concordant with a faint dark band in thin section image.

*Paroedura pictus* (Fig 9B; S19 Fig)

The small grains were distributed in the outer end of the columnar layer whereas larger and fan-shaped grains were situated in the inner columnar layer. The c-axis orientations in the outer columnar layer were somewhat chaotic compared to the well-organized grains in the inner one. Large and fan-shaped grains, which became wider transversally as they go to the inner surface, had a strong alignment that their c-axes are perpendicular to the eggshell surface. The intensity of c-axis alignment became stronger as it goes to the inner surface. In addition, it was observed that the columns in a pit (=chamber-like structure) converged into the top of the chamber (S19 Fig). Those columns were also fan-shaped in morphology and wider arcs headed to the inner surface of the eggshell.

*Paroedura stumpfii* (Fig 9C)

The outer end of the porous layer had small grain size while the inner part of the eggshell had large and fan-shaped grains. The outer porous layer had the most complex c-axis orientation in the eggshell. The grains of the porous layer were extended to the plain layer and they had weakly organized orientation compared to those of the plain layer. The plain layer had well-organized calcite grains, whose c-axis aligned perpendicular to the eggshell surface. The intensity of alignment was the highest on the inner end of the plain layer.

*Phelsuma grandis* (Fig 9D)

The main pattern was the same as *Paroedura* eggshells. However, it is notable that the *Phelsuma grandis* eggshell had much slender calcite grains, which had nearly the same height as whole eggshell thickness. It might accord closely with the observation that *Phelsuma* had well-developed columnar structure compared to other rigid gekkotan eggshells (Fig 4D; Fig 5D2; S5 Fig; [S2]).

*Correlophus ciliatus* (Fig 9E)

As mentioned above, the calcareous layer of soft gekkotan eggshells could be divided into two layers: the outer cap-like structure and the inner stem-like structure composed of calcites and proteins. Like rigid gekkotan eggshells, the calcite grains in the cap-like structures were smaller than those of the stem-like structure. The calcite grains of the stem-like structure were highly porous due to the proteins but they still showed a fan-shaped structure as rigid gekkotan eggshells. The c-axis orientations of calcite crystals in the cap-like structure and outer parts of the stem-like structure were paralleled to the outer surface. On the other hand, the c-axis of calcites in the middle and inner parts of the stem-like structure were aligned perpendicular to the eggshell surface. The intensity of alignment became stronger as it goes to the inner surface like rigid gekkotan eggshells.

*Rhacodactylus leachianus* (Fig 9F)

The overall pattern was the same as *Correlophus ciliatus* eggshell. One important difference was that *Rhacodactylus leachianus* eggshell had deeper outer calcareous layer whose c-axes were paralleled to the surface. As in *Correlophus ciliatus* eggshell, the c-axes of the mixed layer were perpendicular to the eggshell surface.

*Eublepharis macularius* (Fig 9G)

It was difficult to figure out the grain size differences between the cap-like and stem-like structures due to the poor signal of the calcareous layer caused by its extreme thinness (less than 10 μm). The calcite crystals on the cap-like structure had more horizontal c-axis than those of the stem-like structure. In addition, as other gekkotan eggshells, the stem-like structure showed strong c-axis alignment compared to that of cap-like structure.

References

S1. Packard MJ, DeMarco VG. Eggshell structure and formation in eggs of oviparous reptiles. In: Deeming DC, Ferguson MWJ, editors. Egg incubation: its effects on embryonic development in birds and reptiles; 1991. pp. 53–69.

S2. Packard MJ, Hirsch KF. Structure of shells from eggs of the geckos *Gekko gecko* and *Phelsuma madagascariensis*. Can J Zool. 1989;67(3): 746–758.

S3. Schleich HH, Kästle W. Reptile egg-shells SEM atlas. Stuttgart: Gustav Fischer Verlag; 1988.

S4. Osborne L, Thompson MB. Chemical composition and structure of the eggshell of three oviparous lizards. Copeia. 2005;2005(3): 683–692.

S5. Mikhailov KE. Avian eggshells: an atlas of scanning electron micrographs. 1^st^ ed. Tring: British Ornithologists’ Club Occasional Publications. No. 3; 1997.

S6. Zelenitsky DK, Modesto SP, Currie PJ. Bird-like characteristics of troodontid theropod eggshell. Cretac Res. 2002;23: 297–305.

S7. Zelenitsky DK, Modesto SP. New information on the eggshell of ratites (Aves) and its phylogenetic implications. Can J Zool. 2003;81(6): 962–970.

S8. Packard MJ, Thompson MB, Goldie KN, Vos M. Aspects of shell formation in eggs of the tuatara*, Sphenodon punctatus*. J Morphol. 1988;197(2): 147–157.

S9. Deeming DC. Eggshell structure of lizards of two sub-families of the Gekkonidae. Herpetol J. 1988;1: 230–234.

S10. Andrews RM. Novel eggshell of the New Caledonian Diplodactylid gecko species *Correlophus ciliatus* (=*Rhacodactylus ciliatus*). J Herpetol. 2017;51(2): 173–177.
