# Supplementary material for: A comparative study of eggshells of Gekkota with morphological, chemical compositional and crystallographic approaches and its evolutionary implications
Source: PLoS One. 2018 Jun 22;13(6):e0199496. doi: 10.1371/journal.pone.0199496 (PMC6014675; doi:10.1371/journal.pone.0199496)
Supplement: S1 Table — (DOCX) [file pone.0199496.s002.docx]

**Supplementary Information**

A comparative study of eggshells of Gekkota with morphological, chemical compositional and crystallographic approaches and its evolutionary implications

Seung Choi^1, *^, Seokyoung Han^1^, Noe-Heon Kim^1^, Yuong-Nam Lee^1, *^

^1^ School of Earth and Environmental Sciences, Seoul National University, Seoul, 08826, South Korea

**Supplementary Table S1**. Gekkotan eggshell micrographs from Polarized Light Microscope or SEM observation in the literatures. Modified from [S1].

| Family Name | Scientific Name | Reference |
| --- | --- | --- |
| Gekkonidae | *Chondrodactylus angulifer*  *Cyrtopodion kotschyi* (=*Cyrtodactylus kotschyi*)  *Gehyra mutilata*  *Gekko gecko*  *Hemidactylus bouvieri*  *Hemidactylus mabouia*  *Hemidactylus turcicus*  *Lepidodactylus lugubris*  *Paroedura pictus*  *Paroedura stumpfii*  *Phelsuma agalegae*  *Phelsuma grandis*  *Phelsuma guentheri*  *Phelsuma madagascarensis* | S2  S3  S2  S3–5; **This study**  S3  S6  S3;S7;S8  S8  **This study**  **This study**  S2  **This study**  S2;S9  S3;S4;S10 |
| Phyllodactylidae | *Gymnodactylus caspicus*  *Ptyodactylus hasselquistii*  *Tarentola delalandii*  *Tarentola gigas*  *Tarentola mauritanica* | S11  S3  S3;S12  S3  S3;S10;S13;S14 |
| Diplodactylidae | *Correlophus ciliatus*  *Rhacodactylus leachianus* | **This study**  **This study** |
| Eublepharidae | *Eublepharis macularius*  *Hemitheconyx caudicinctus* | S2;S3;S8;**This study**  S2 |

References

S1. Packard MJ, DeMarco VG. Eggshell structure and formation in eggs of oviparous reptiles. In: Deeming DC, Ferguson MWJ, editors. Egg incubation: its effects on embryonic development in birds and reptiles; 1991. pp. 53–69.

S2. Deeming DC. Eggshell structure of lizards of two sub-families of the Gekkonidae. Herpetol J. 1988;1:230–234.

S3. Schleich HH, Kästle W. Reptile egg-shells SEM atlas. Stuttgart: Gustav Fischer Verlag; 1988.

S4. Packard MJ, Hirsch KF. Structure of shells from eggs of the geckos *Gekko gecko* *and Phelsuma madagascariensis*. Can J Zool. 1989;67(3):746–758.

S5. Hirsch KF, Quinn B. Eggs and eggshell fragments from the Upper Cretaceous Two Medicine Formation of Montana. J Vert Paleontol. 1990;10(4):491–511.

S6. Grine FE, Kitching JW. Scanning electron microscopy of early dinosaur egg shell structure: a comparison with other rigid sauropsid eggs. Scanning Microsc. 1987;1(2):615–630.

S7. Packard MJ, Packard GC, Boardman TJ. Structure of eggshells and water relations of reptilian eggs. Herpetologica. 1982:136–155.

S8. Packard MJ, Hirsch KF. Scanning electron microscopy of eggshells of contemporary reptiles. Scan Electron Microsc. 1986;4:1581–1590.

S9. Deeming DC, Ferguson MWJ. Incubation and embryonic development in reptiles and birds. In: Tullett SG, editor. Avian Incubation; 1991. pp. 3–37

S10. Hirsch KF, Packard MJ. Review of fossil eggs and their shell structure. Scanning Microsc. 1987;1(1):383–400.

S11. Mikhailov KE. Fossil and recent eggshell in amniotic vertebrates: fine structure, comparative morphology and classification. Spec Pap Palaeontol. 1997;56:1–80.

S12. Hirsch KF, Krishtalka L, Stucky R. Revision of the Wind River Faunas, Early Eocene of Central Wyoming. Part 8. First fossil lizard egg (? Gekkonidae) and list of associated lizards. Ann Carnegie Mus. 1987;56(12):223 –230.

S13. Erben HK, Newesely H. Kristalline bausteine und mineralbestand von kalkigen eischalen. Biomineralisation. 1972;6:32–48.

S14. Krampitz GP, Erben HK. Kriesten K. Über Aminosaurenzusammensetzung und Struktur von Eischalen. Biomineralisation. 4:87–99.
