# Supplementary figures and images for: A comparative study of eggshells of Gekkota with morphological, chemical compositional and crystallographic approaches and its evolutionary implications
Source: PLoS One. 2018 Jun 22;13(6):e0199496. doi: 10.1371/journal.pone.0199496 (PMC6014675; doi:10.1371/journal.pone.0199496)

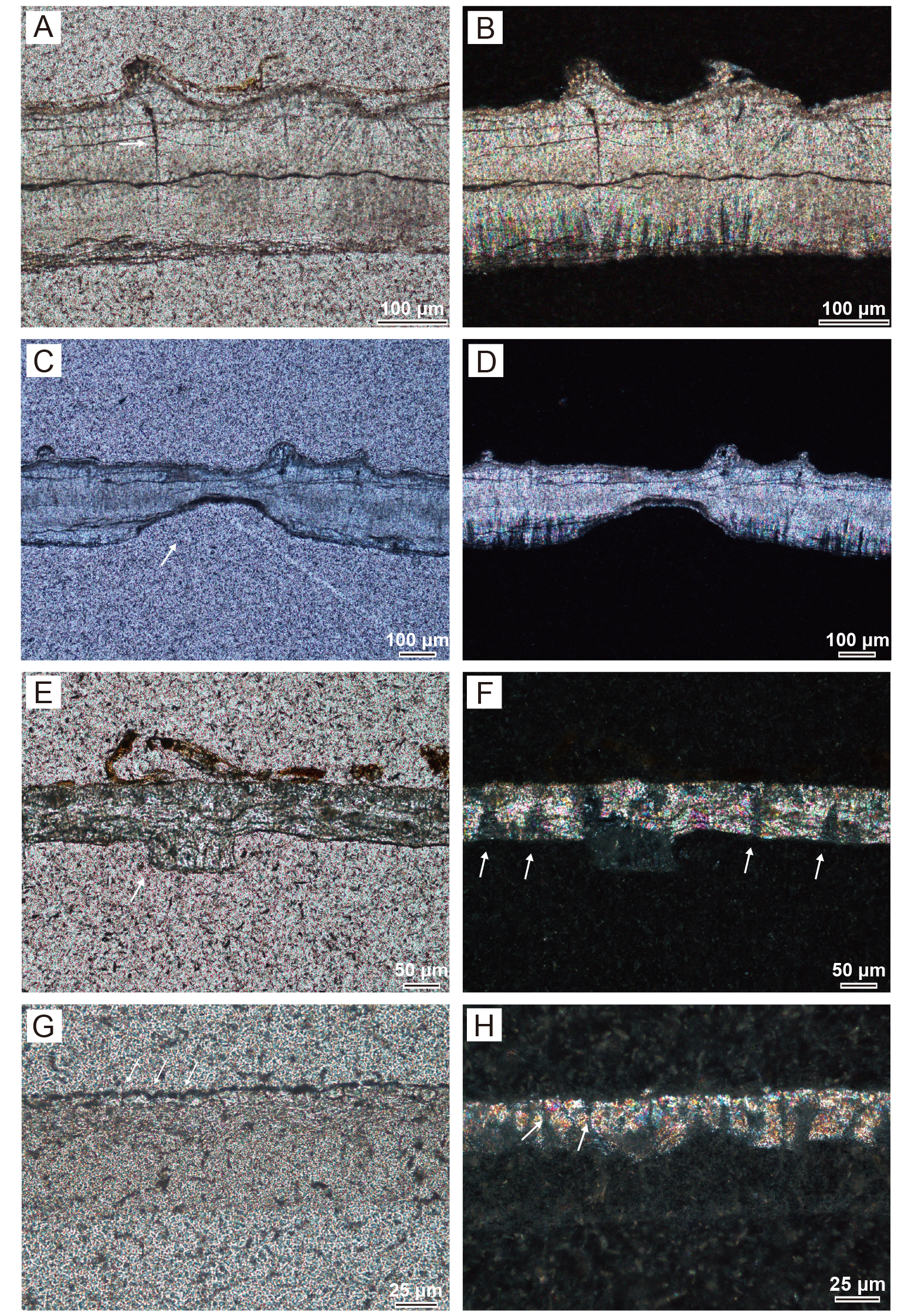

Supplement: S1 Fig — Outside of eggshell is up. (A–D) Gekko gecko. A white arrow in A points the pore-like structure. Note that it becomes thinner toward the inner surface. A white arrow in C shows a big concavity in the inner surface. (E–F) Paroedura stumpfii. A white arrow in E points a calcite concretion. White arrows in F mark the triangular or columnar extinction pattern. (G–H) Eublepharis macularius. Note the pore-like structures are present between the shell units (white arrows). (TIF) [file pone.0199496.s003.tif]

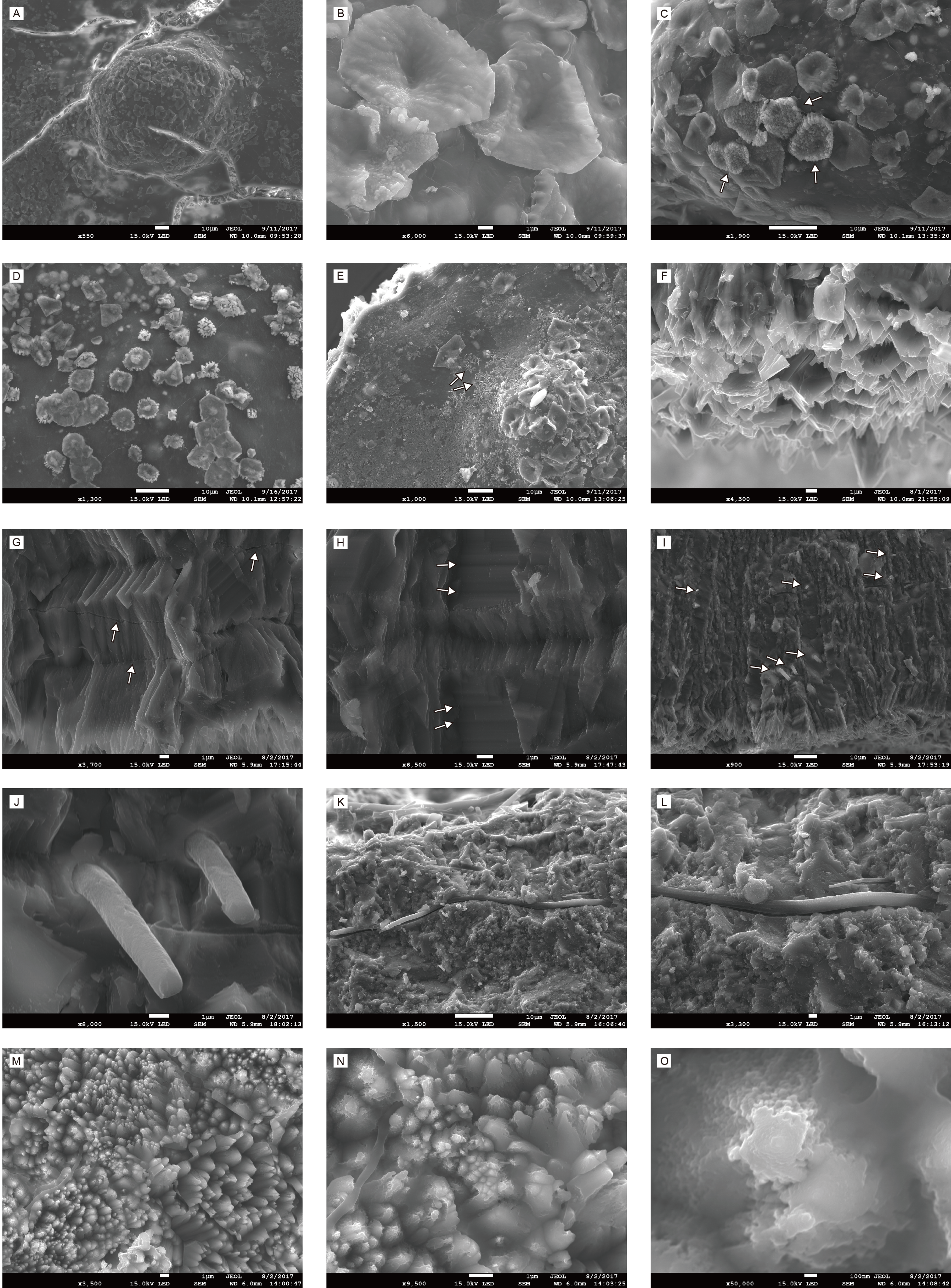

Supplement: S2 Fig — (A) An enlarged view of a nodular ornamentation covered with polygonal calcareous structures. (B) An enlarged view of polygonal calcareous structures. Note the depression in the central region. (C) Different types of calcareous structure in the outer surface. White arrows indicate the calcareous structure composed of minute columns. (D) Polygonal calcareous structures on the surface of the covering layer which might be in different developmental stages. (E) Starfish-like structures in the covering layer (white arrows). (F) Needle-like innermost tips of jagged columnar structure. (G) Horizontal fissures observed in the inner part of the columnar layer (white arrows). (H) A magnified view of horizontal fissures. White arrows mark the stacked calcite plates. (I) Protein fibers distributed in the eggshell. (J) An enlarged view of protein fibers. Note that the diameter of protein fiber is consistent with those of vesicles. (K–L) Protein fibers exposed in lateral view. (M–N) Acute tips of needle-like structures in inner view. Note that they can be grouped by their directions. (O) Stacked plate-like structure of the needle-like structures in high magnification. (TIF) [file pone.0199496.s004.tif]

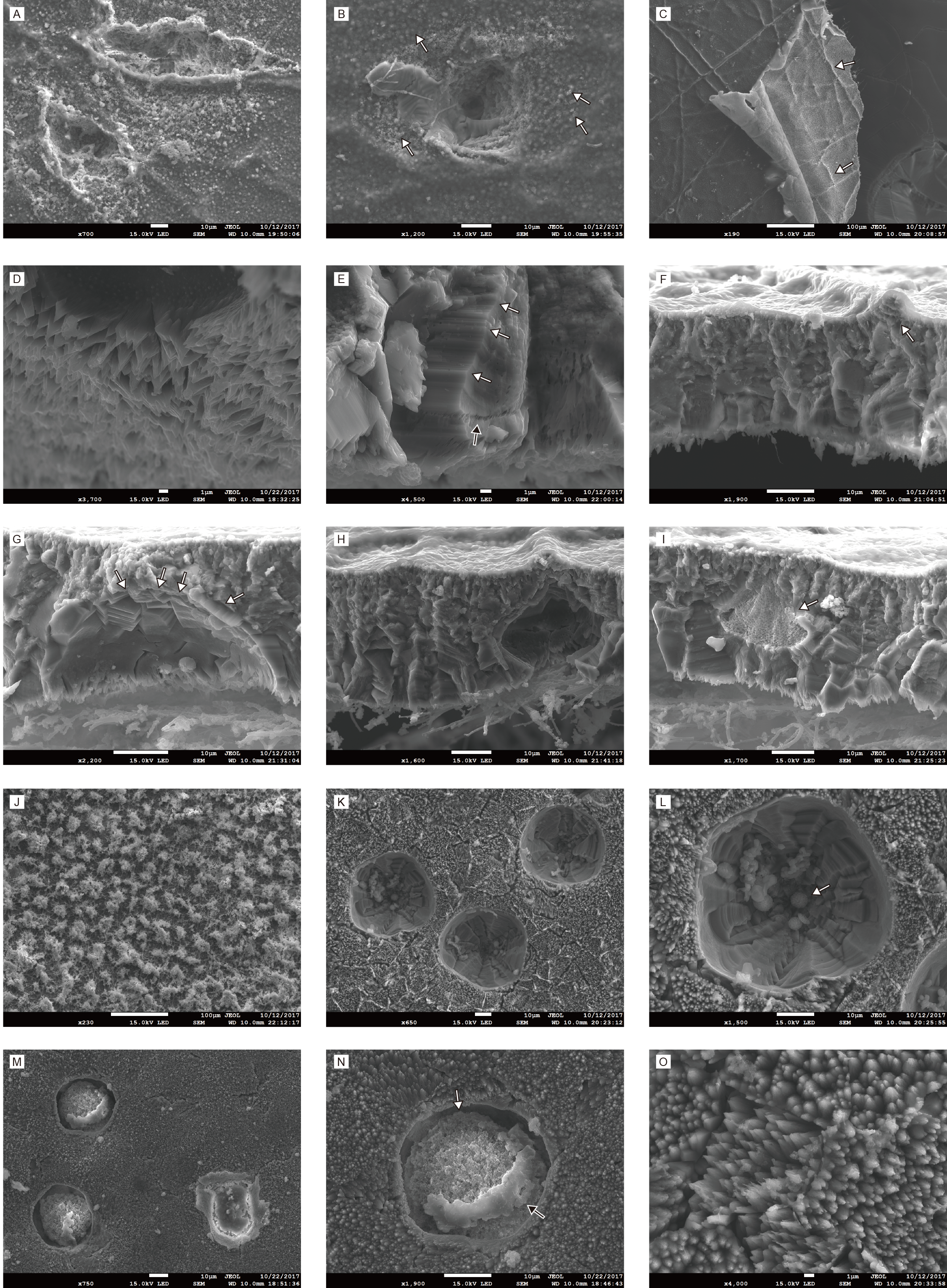

Supplement: S3 Fig — (A–B) Pore-like structures. Note spherical shell elements at the external surface of the eggshell (white arrows) (C) When the covering layer is peeled off, the calcareous ridge-like ornamentation is exposed (white arrows), confirming that they are not protein fibers. (D) Needle-like tips in the inner surface of the eggshell. (E) Stacked calcite plates (white arrows) and horizontal fissure (a black arrow) in the inner columnar layer. (F) Radial view of the ridge-like ornamentation, showing that ornamentation is composed of calcites. (G–H) A chamber-like structure. Several columnar structures converge to the top of the chamber (white arrows). (I) A honeycomb-like structure in a fractured chamber-like structure (a white arrow). (J) Shell membrane interwoven with spongy calcite granules. (K–L) An enlarged view of pits in the inner surface. Note the converging columnar structure in the wall of pits and porous granules in the middle of a pit (a white arrow). (M–N) About half of the pits are filled with calcareous materials (a white arrow) and are covered with membrane-like structure which is possibly residual materials of the blocky layer (a black arrow). (O) Acute tips of needle-like structures. Note that they can be grouped by their orientations. (TIF) [file pone.0199496.s005.tif]

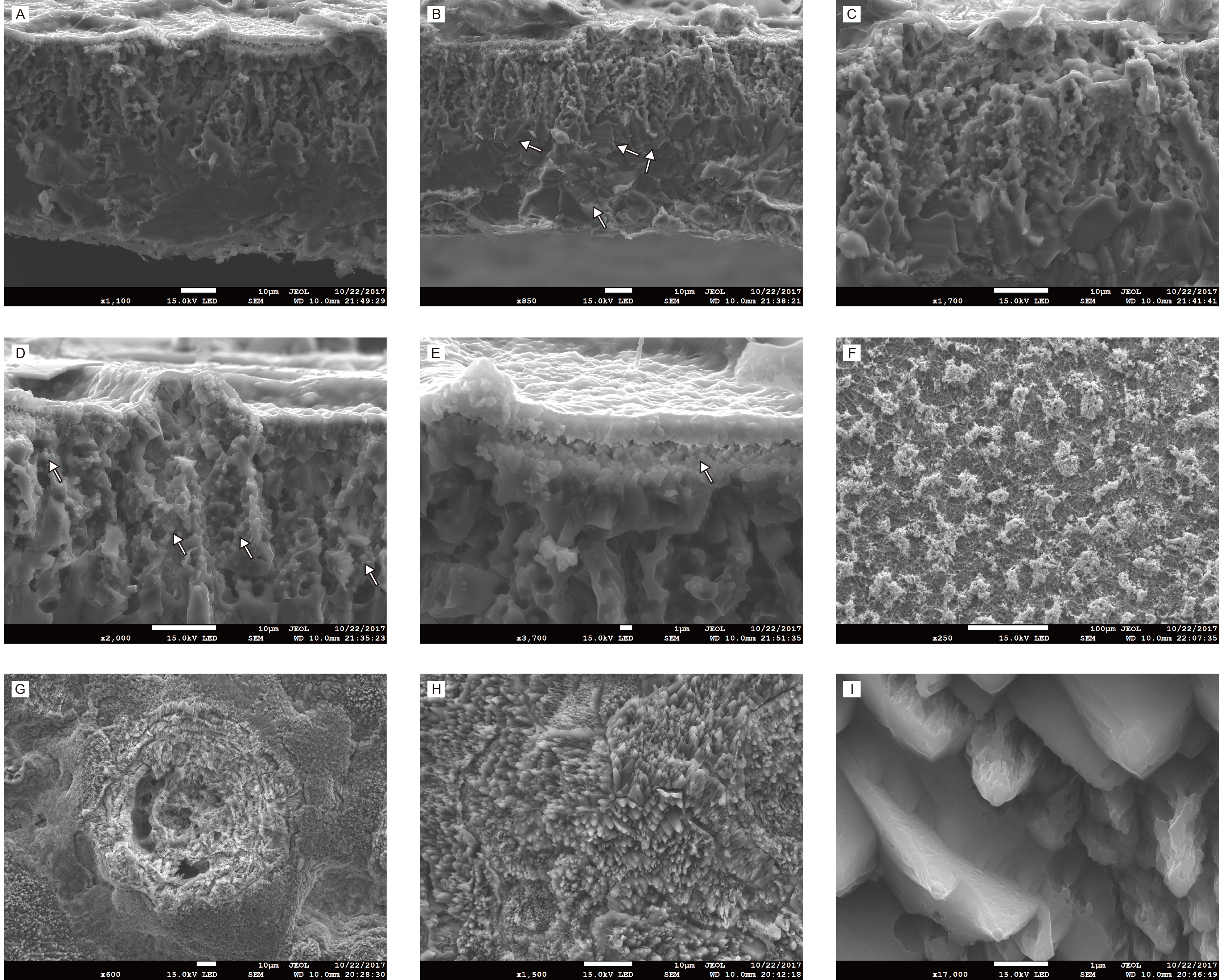

Supplement: S4 Fig — (A) A radial view of the eggshell. The columnar structure is absent in many different sections. (B) Stacked calcite plates (white arrows) are observed in the plain layer. Nevertheless, their orientations are irregular compared to the other rigid gekkotan eggshells. (C) An enlarged view of the porous layer. Note that the shell constituting calcite granules are apparent. (D) Ornamentation in radial view. The spherical shell elements are conspicuous at the porous layer (white arrows). (E) The covering layer is slightly separated from the main eggshell (a white arrow). (F) The shell membrane interwoven with spongy calcite granules. (G) A protruding calcite concretion is filled with needle-like structures. (H) Needle-like structures in the inner surface of the plain layer. Note the orientations of these structures. (I) An enlarged view of needle-like structures shows stacked calcite plates as Gekko gecko eggshell. (TIF) [file pone.0199496.s006.tif]

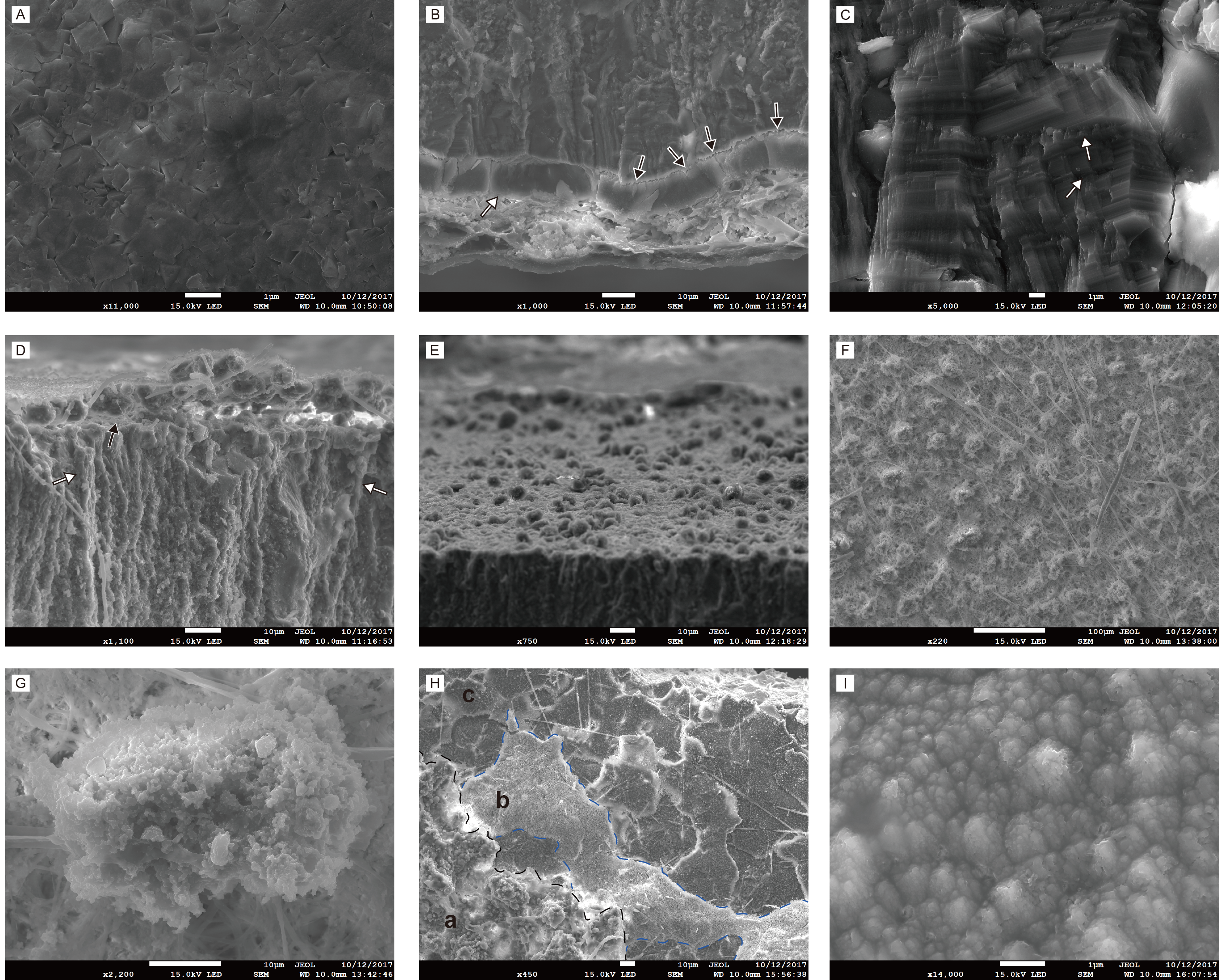

Supplement: S5 Fig — (A) High magnification view of the outer surface of the covering layer. Minute calcite crystals are fused together. (B) An enlarged view of the inner part of the eggshell. The blocky layer is laterally continuous (a white arrow). Black arrows mark the boundary between the blocky and columnar layers. Note needle-like structures. (C) Prominent stacked calcite plates in the inner part of the columnar layer and intermittent horizontal fissures (white arrows). (D) Columnar structures are traceable to the outer surface of the eggshell (white arrows). Note that spherical calcite granules are interwoven with protein fibers (= surface layer; a black arrow). (E) When the covering layer is peeled off, the surface layer is exposed showing spherical calcite granules. (F) Innermost shell membrane interwoven with spongy calcite granules. (G) A magnified view of spongy calcite granule, which is composed of spherical subgrains. (H) This inner view shows three consecutive layers: (a) the shell membrane (outlined by black dashed line), (b) the blocky layer (outlined by blue dashed line), and (c) the inner surface of the columnar layer. The needle-like tips have different cluster orientations. (I) Needle-like structures in the inner surface of the columnar layer. Note that they are comparatively blunt than other rigid gekkotan eggshells, presumably due to the failed embryogenesis. (TIF) [file pone.0199496.s007.tif]

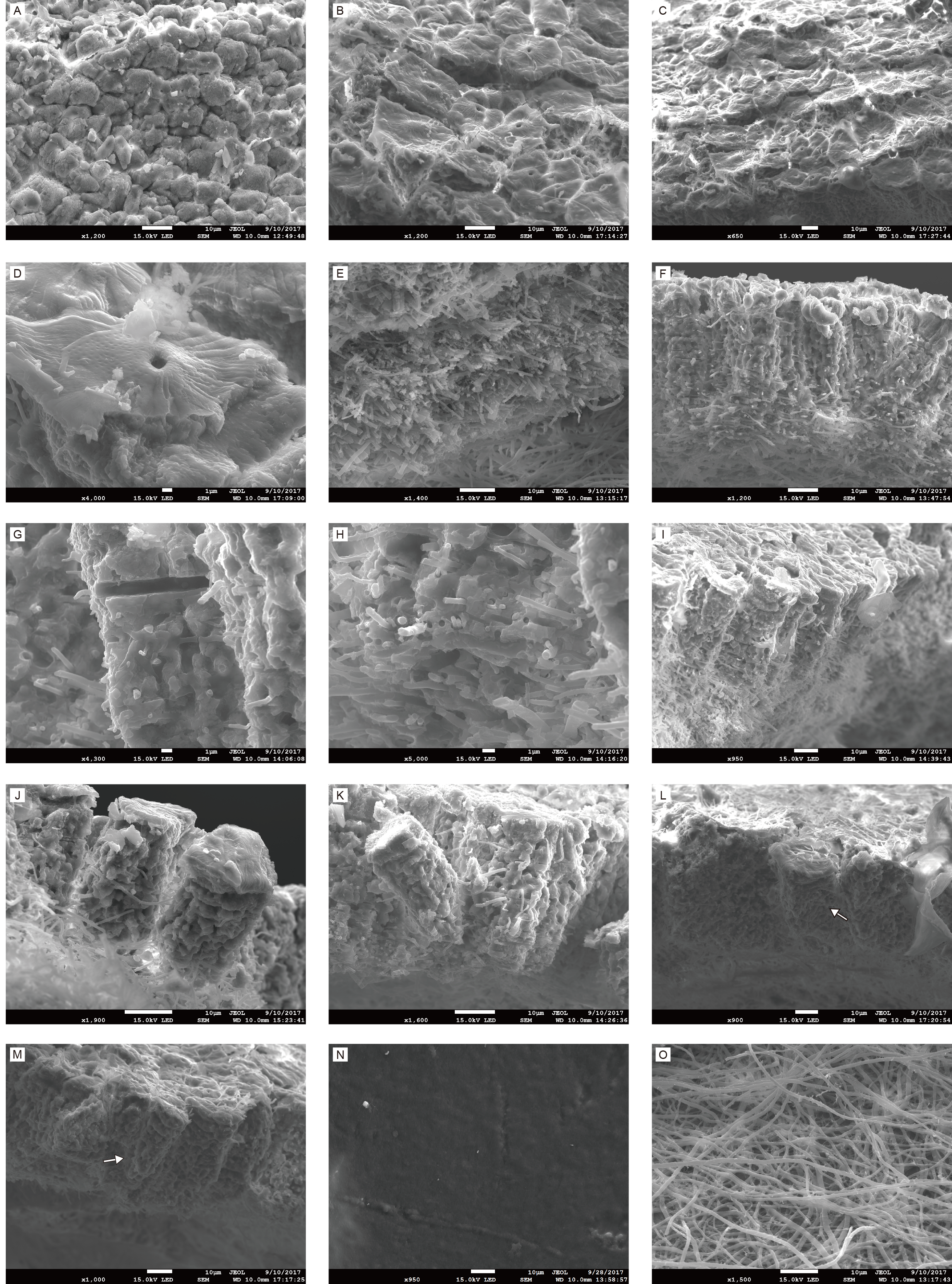

Supplement: S6 Fig — (A) A magnified view of suboval granules in the outer surface. (B–D) Occasionally, volcano-like convex structures make up the outer surface of the eggshell. (E) The shell membrane from outer view. (F–H) A columnar calcareous layer composed of both stem- and cap-like structures. The crystalline calcites are intertangled with protein fibers. (I–M) Columnar or wedge-like structures are present in the calcareous layer (white arrows). Note that each column is composed of cap-like and stem-like structures as tuatara eggshell [15]. (N) The boundary layer seen from the inner view. (O) The boundary layer seen from the outer view. Note that fibers in the boundary layer are more robust and tough compared to those of the shell membrane (compare (O) with (E)). (TIF) [file pone.0199496.s008.tif]

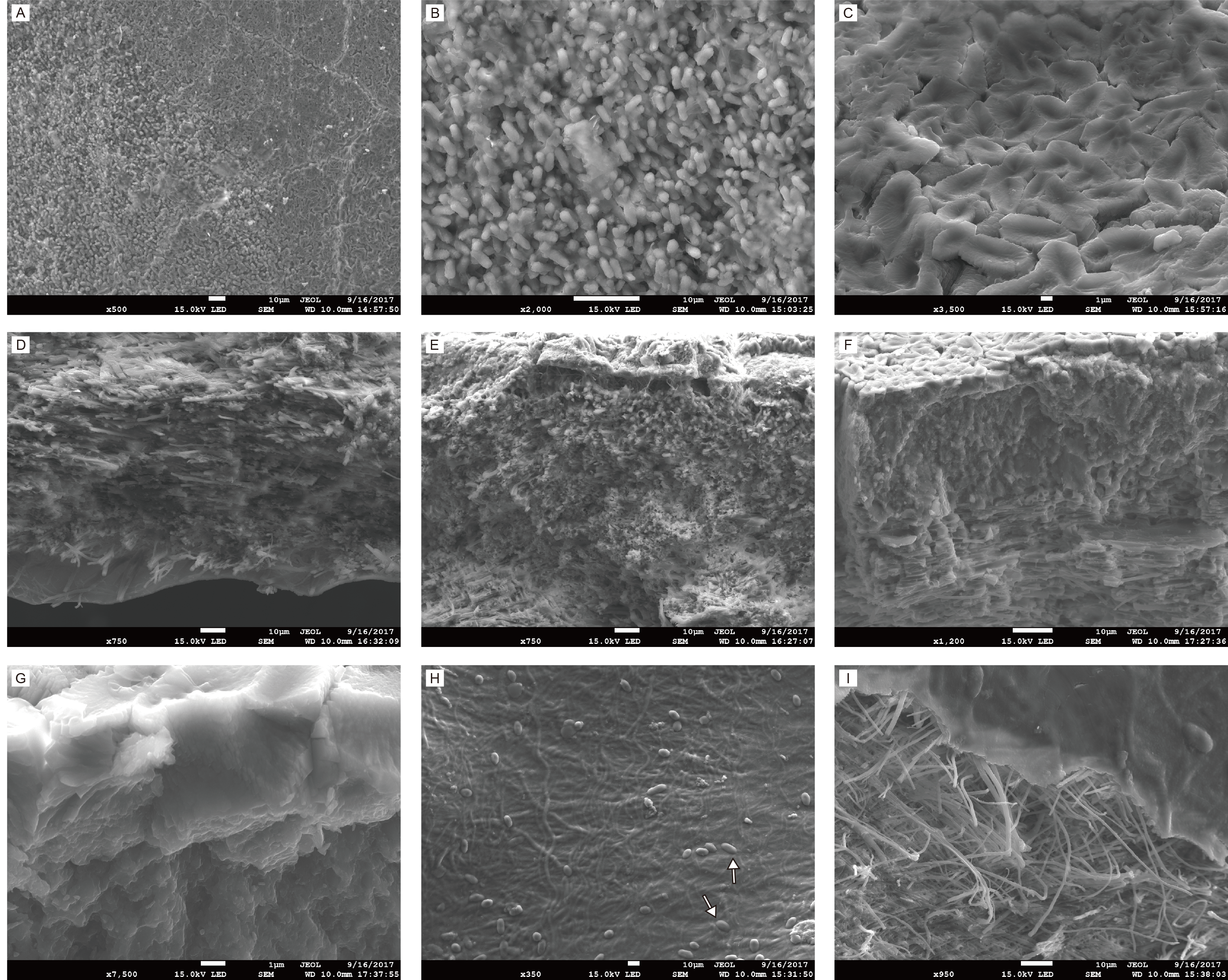

Supplement: S7 Fig — (A) A gradual boundary between the capsule-like and flattened granules in the outer surface. (B) An enlarged view of the capsule-like granules. (C) Enlarged view of the flattened granules. It is notable that the central concavity of the granules is similar to “cup-like central depression” of tuatara eggshell [15]. (D) The shell membrane and boundary layer. (E) Calcareous outer part of the eggshell (Mixed and crystalline layers). Note the coexistence of calcites and protein fibers. (F) The outer part of the mixed layer where few protein fiber exists. Note the absence of columnar or wedge-like structure. (G) An enlarged view of the outermost crystalline layer (= granules in the outer surface). (H) The boundary layer from inner view. Protein fibers are more conspicuous than those of Correlophus ciliatus eggshell. Elongated globular structures may be microbes (white arrows). (I) Thick and tough protein fibers constituting the boundary layer is observed from the inner view. (TIF) [file pone.0199496.s009.tif]

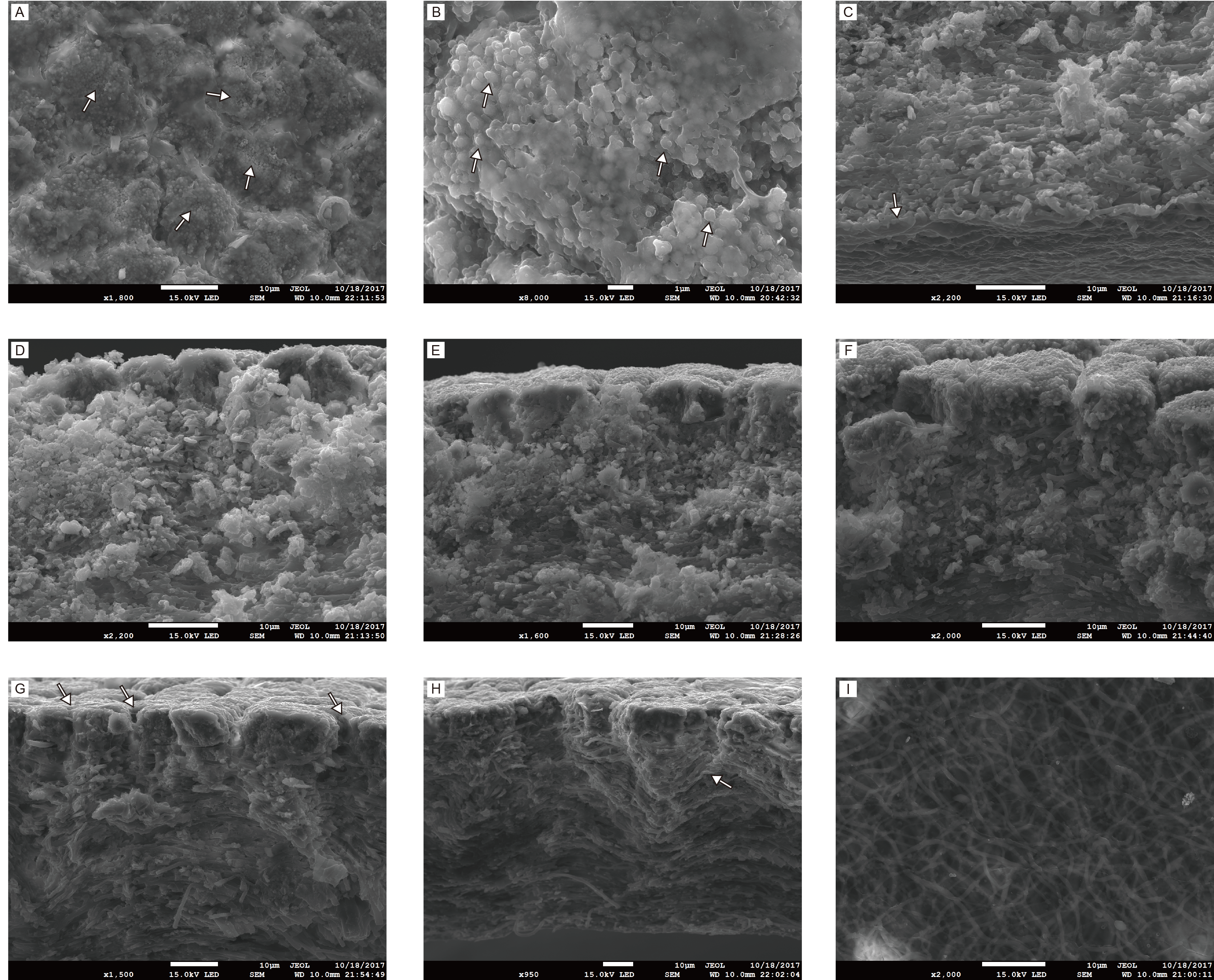

Supplement: S8 Fig — (A) Closely packed calcareous blocks in the outer surface. White arrows mark the central depressions. (B) An enlarged view of a calcareous block composed of spherical shell elements (white arrows). (C) The shell membrane and boundary layers. A white arrow marks the boundary layer. (D–F) The outer part of the shell membrane and the calcareous layer composed of stem- and cap-like structures. Note that few protein fibers exist in the cap-like structure. (G) The gaps between the calcite blocks make the pore-like structure (white arrows). (H) A calcite block that has wedge-like “stem” in the lower part. Note the association between the “stem” and the wave-like pattern of protein fibers. (I) Relatively thick and tough protein fibers (compared to those of shell membrane) exist in the boundary layer. (TIF) [file pone.0199496.s010.tif]

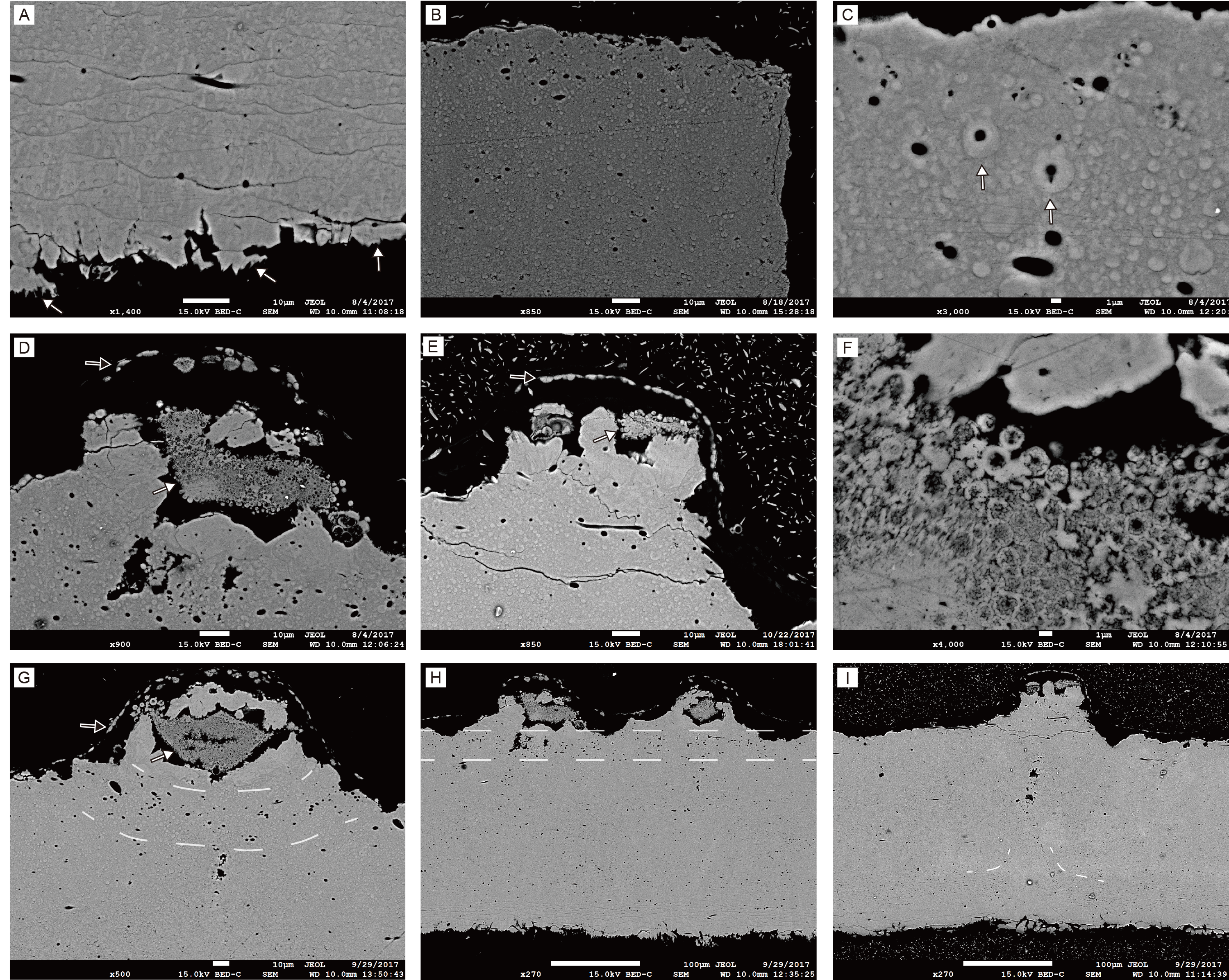

Supplement: S9 Fig — (A) An enlarged view of sub-parallel horizontal accretion lines. Note needle-like structures (white arrows). (B) An enlarged view of the outer margin of the eggshell. The circular structures are well-developed near the outer surface. (C) The typical circular structure with a central hole (white arrows). (D–E) Detailed view of ornamentations. A bulbous structure within the ornamentation is marked by a white arrow. The polygonal calcareous structures above the ornamentation are marked by a black arrow. Note that the gap between the ornamentation and linear polygonal grains is filled with proteins (= the covering layer) which is invisible in BSE images. (F) An enlarged view of bulbous structures. (G–H) Vesicles are often highly concentrated near the ornamentations (bounded by dashed lines) and the outer surface. The arrows point the same structure as in D–E. (I) Note that accretion lines are bent near the pore-like structure (dashed lines). (TIF) [file pone.0199496.s011.tif]

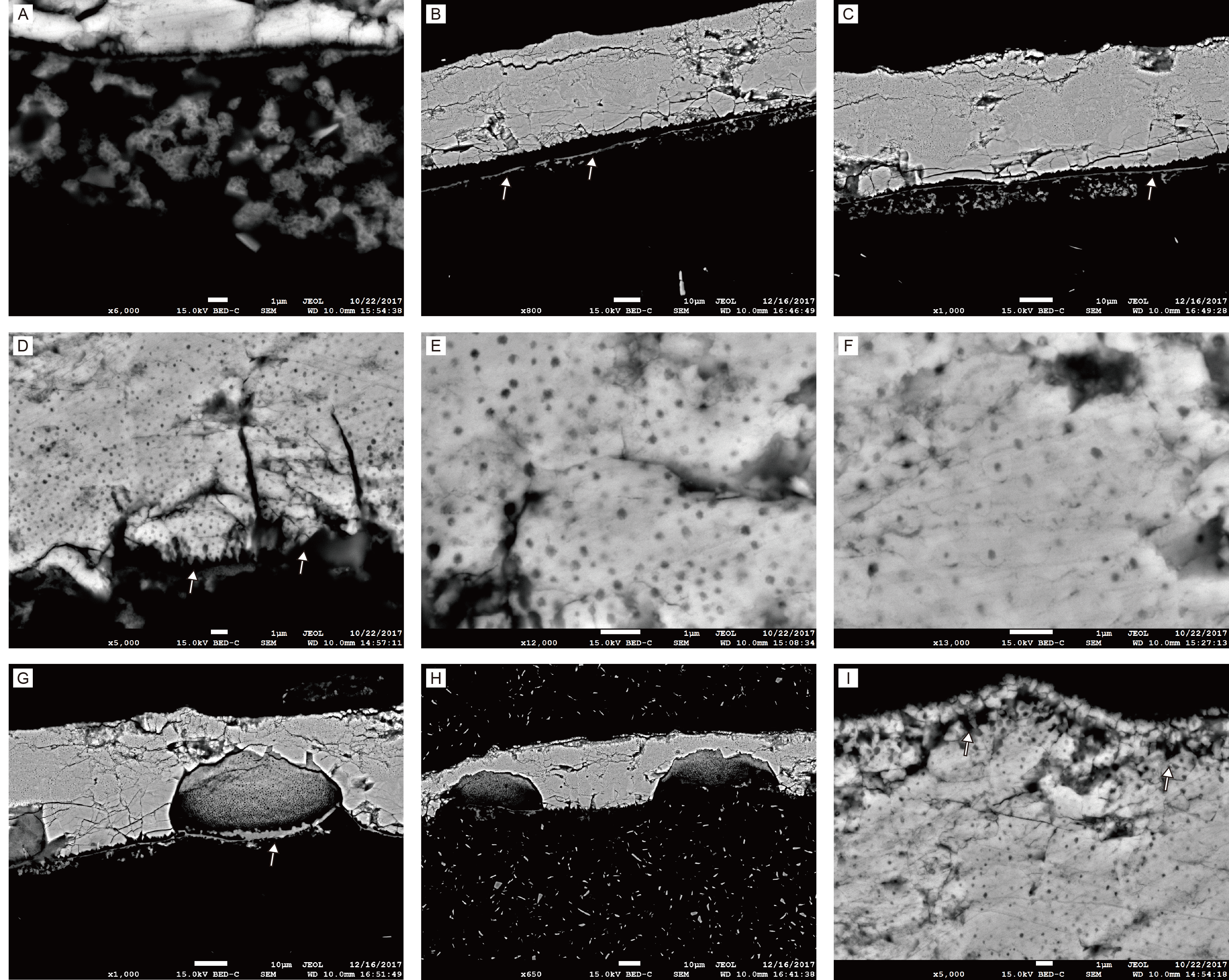

Supplement: S10 Fig — (A) An enlarged view of spongy calcite granules in the shell membrane. (B–C) Laterally continuous blocky layer is present but merely observed in this sample (white arrows). (D) The inner end of the columnar layer shows the needle-like structures (white arrows). (E–F) Abundant circular structures with a central hole. This structure is more clearly seen in the outer region of the columnar layer. (G–H) Radial view of the chamber-like structures. Some of the chamber-like structures show shrunken calcareous matters without the blocky layer (H) whereas unaffected calcareous material is covered with the well-developed blocky layer (an arrow in G). (I) A ridge-like ornamentation. A covering layer is slightly separated from the columnar layer (white arrows). (TIF) [file pone.0199496.s012.tif]

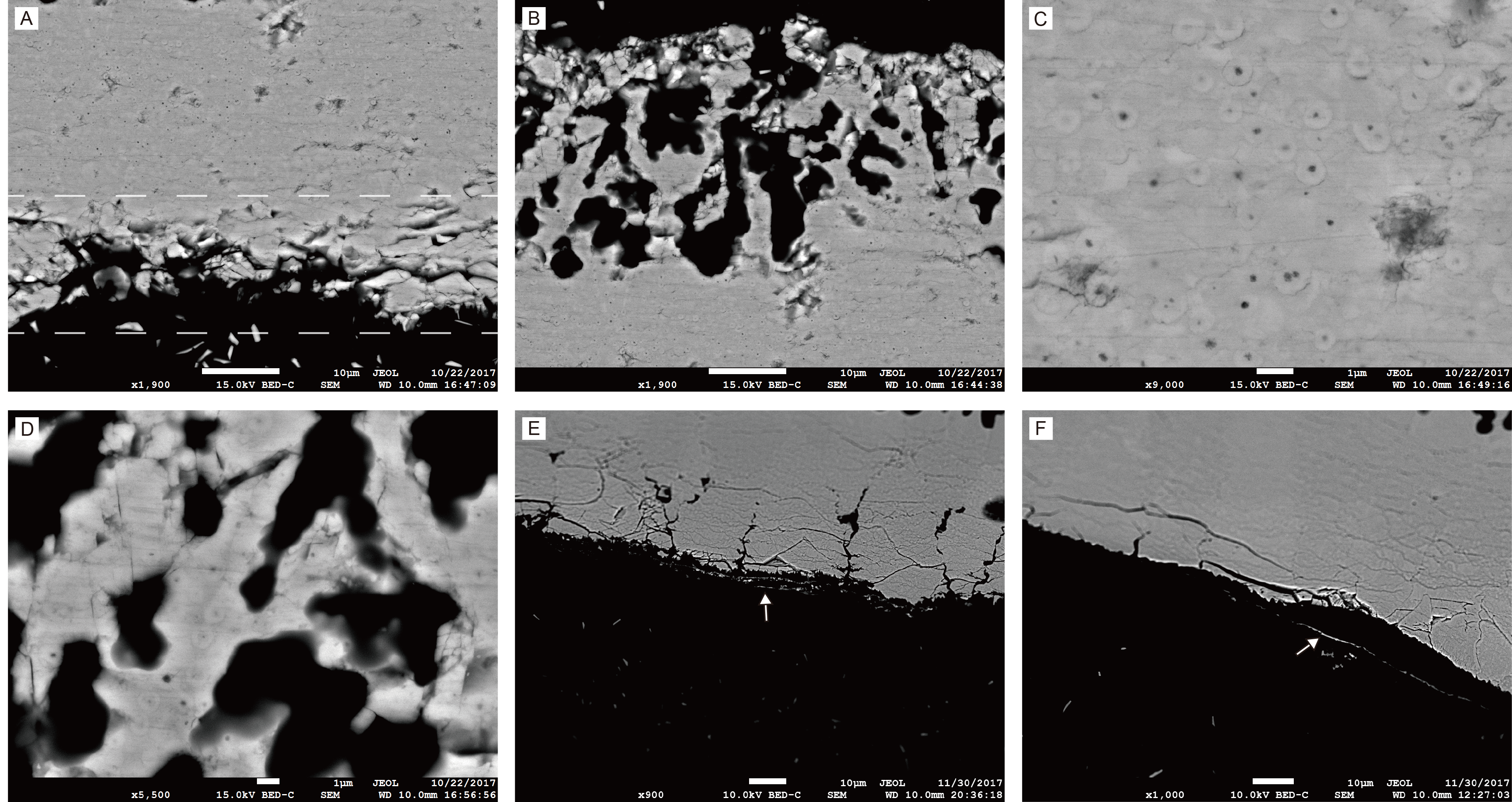

Supplement: S11 Fig — (A) The inner part of the plain layer. Note the absence of circular structures in this part (bounded by dashed lines). (B) An enlarged view of the porous layer which is highly irregular in shape. (C–D) The circular structures are well-developed in the plain layer, while those of the porous layer are weakly-developed. (E–F) The residual blocky layer is rarely observed beneath the main eggshell (white arrows). (TIF) [file pone.0199496.s013.tif]

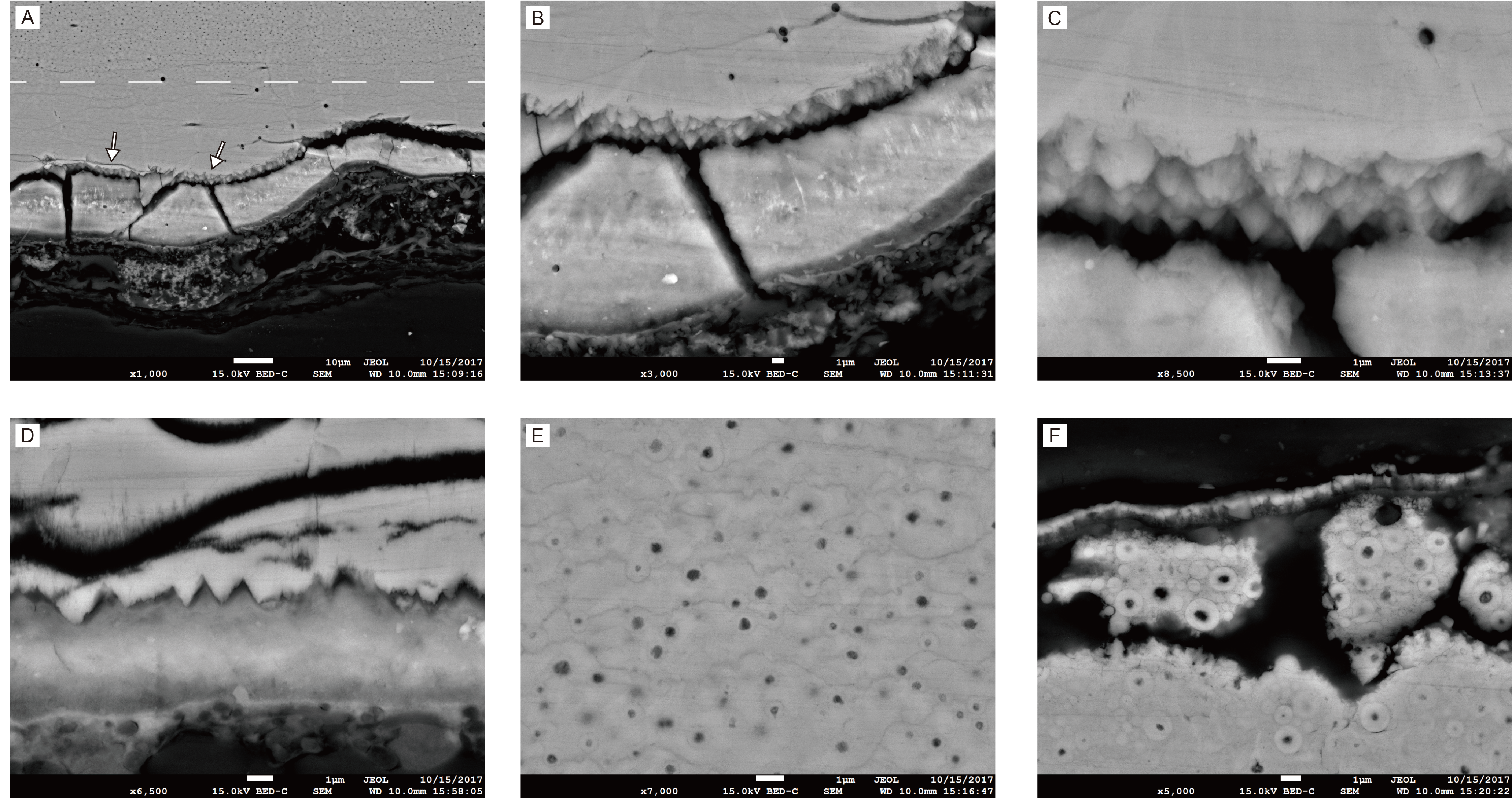

Supplement: S12 Fig — (A–C) An enlarged view of the inner region of eggshell. Note needle-like structures (white arrows in A). The circular structure is absent in the inner one-fourth of the columnar layer (bounded by a dashed line in A). (D) The columnar and blocky layers are interlocked by needle-like structure of the columnar layer. (E) Detailed view of circular structures. This structure is highly concentrated in the middle of the columnar layer. (F) Bud-like ornamentation composed of circular structures. (TIF) [file pone.0199496.s014.tif]

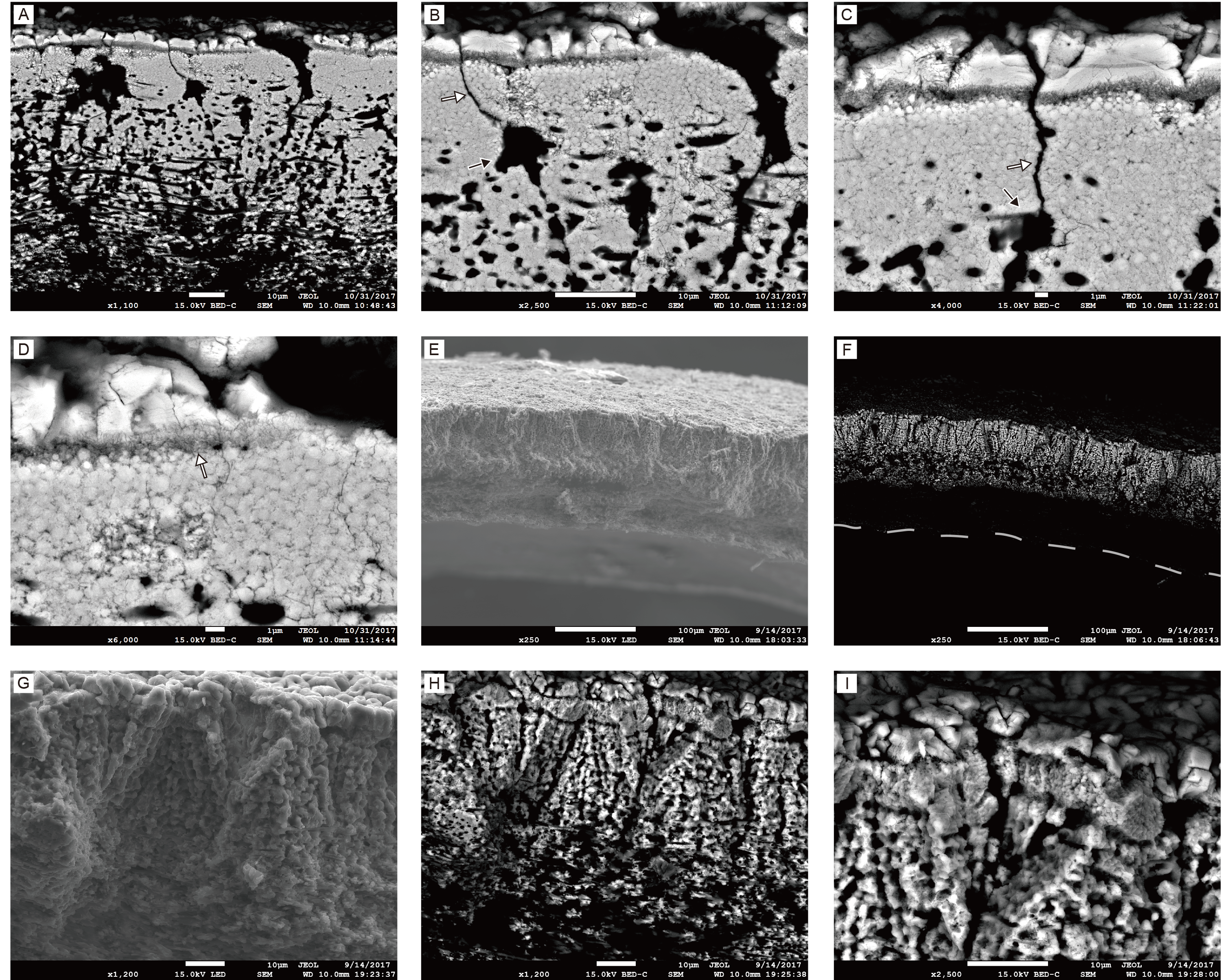

Supplement: S13 Fig — (A–C) Magnified views of the outer calcareous layer. Note pore-like structures (a white arrow) and concomitant chamber-like structures (a black arrow). (D) An enlarged view of the boundary between the stem-like and cap-like structures (a white arrow). Note that stem-like structure is composed of granular calcites whereas the cap-like structure is massive. (E–F) Radial view of the whole eggshell in SE (E) and BSE (F) images. The boundary layer of the eggshell is marked by a dashed line in (F). It confirms the existence of calcites to the middle of the Correlophus ciliatus eggshell. (G–I) Radial view of the calcareous layer of eggshell in SE (G) and BSE (H–I) images. Note that high porosity in the calcareous layer is caused by the presence of protein fibers. (TIF) [file pone.0199496.s015.tif]

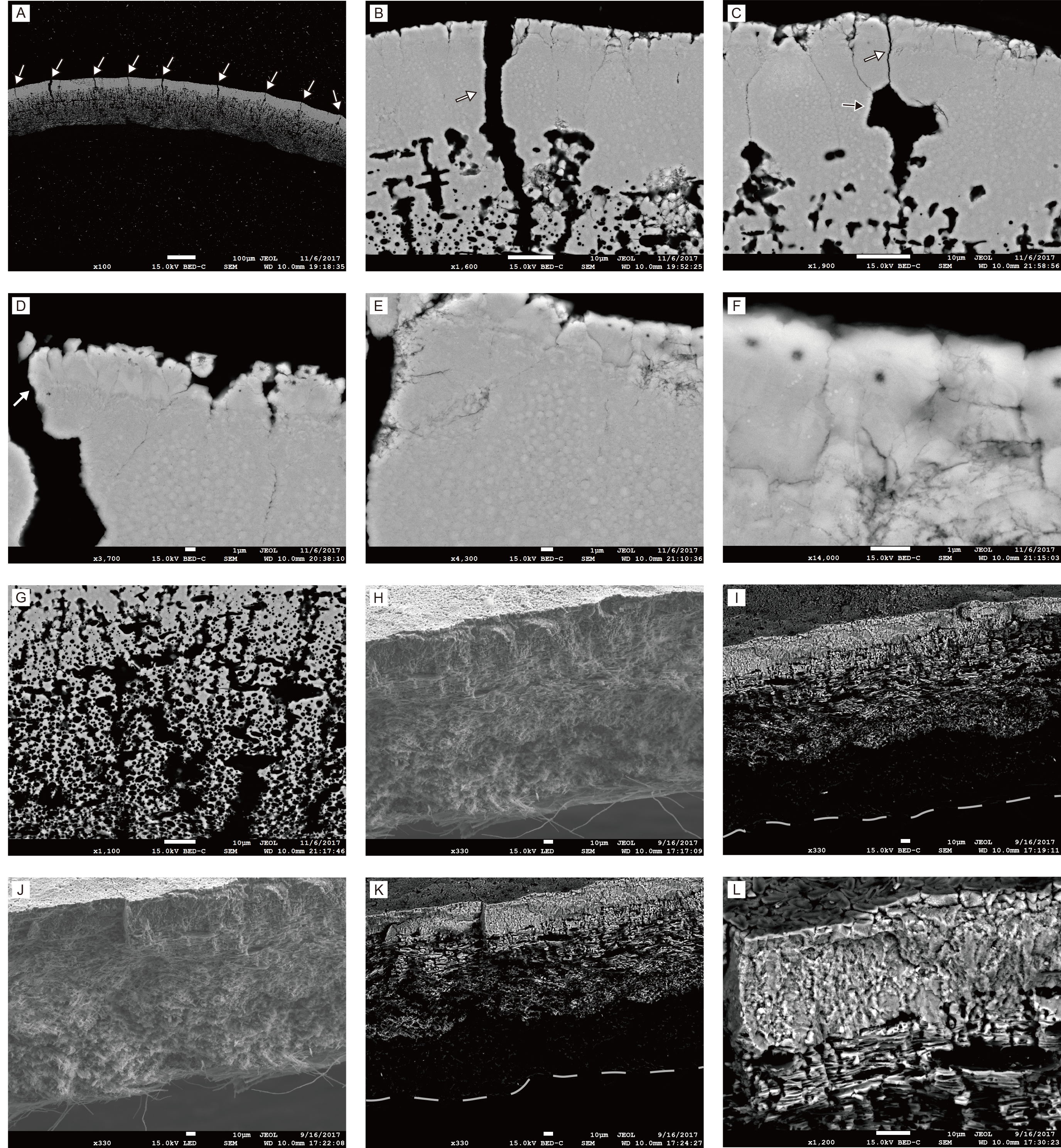

Supplement: S14 Fig — (A) Pore-like structures are regularly distributed in the outer calcareous layer (white arrows). (B–C) Pore-like (white arrows) and associated chamber-like structures (a black arrow). (D) A boundary between the crystalline and mixed layers (a white arrow). Note that outermost crystalline layer is massive. (E) Detailed view of the outer mixed layer. Note granular calcites. (F) An enlarged view of the crystalline layer. Note that some of them have a central hole. (G) Most of the calcareous layer contain abundant protein fibers (dark portion) and calcites. (H–K) Radial view of the whole eggshell in SE (H, J) and corresponding BSE (I, K) images. The boundary layer of the eggshell is marked by a dashed line in I and K. Calcites exist to the middle of the eggshell. (L) An enlarged view of the outer compact calcareous layer. Note that wedge- or column-like structure is absent. (TIF) [file pone.0199496.s016.tif]

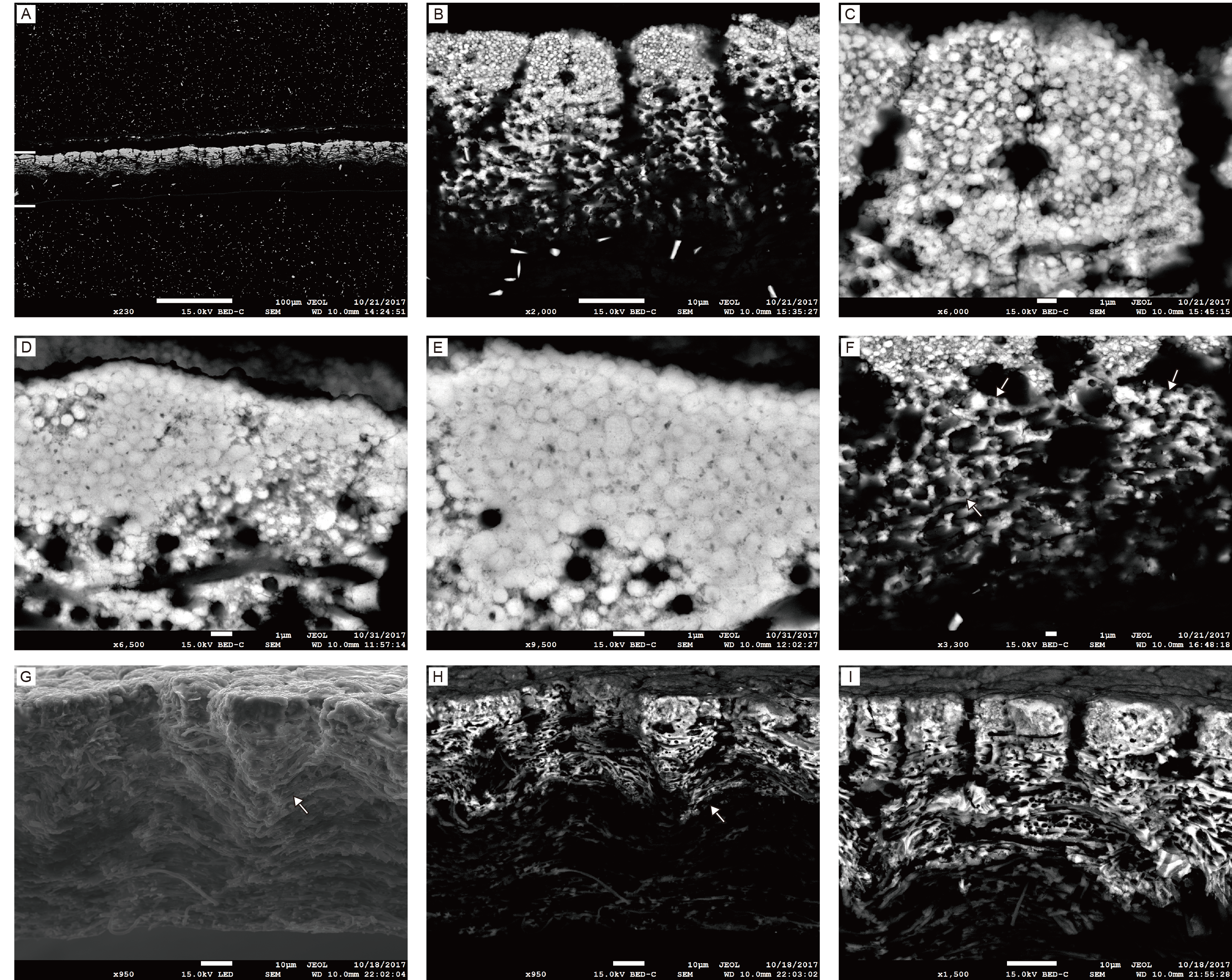

Supplement: S15 Fig — (A) The wave-like pattern in the lower part of a stem-like structure is clearly seen in low magnification. The eggshell thickness is marked by two white bars on the left. (B–E) Detailed view of the dense cap-like structure. D and E show well-polished surfaces so that sections of calcite granules are more clearly exposed. Note holes in the calcite granules. (F) The stem-like structure is a mixture of calcites and protein fibers. Note protein fibers (white arrows). (G–H) White arrows in G and H point stem-like structures. Also, note the wave-like undulation of protein fibers below the wedge-like shell unit. (I) Another “stem” of the column shows their close association with protein fibers. (TIF) [file pone.0199496.s017.tif]

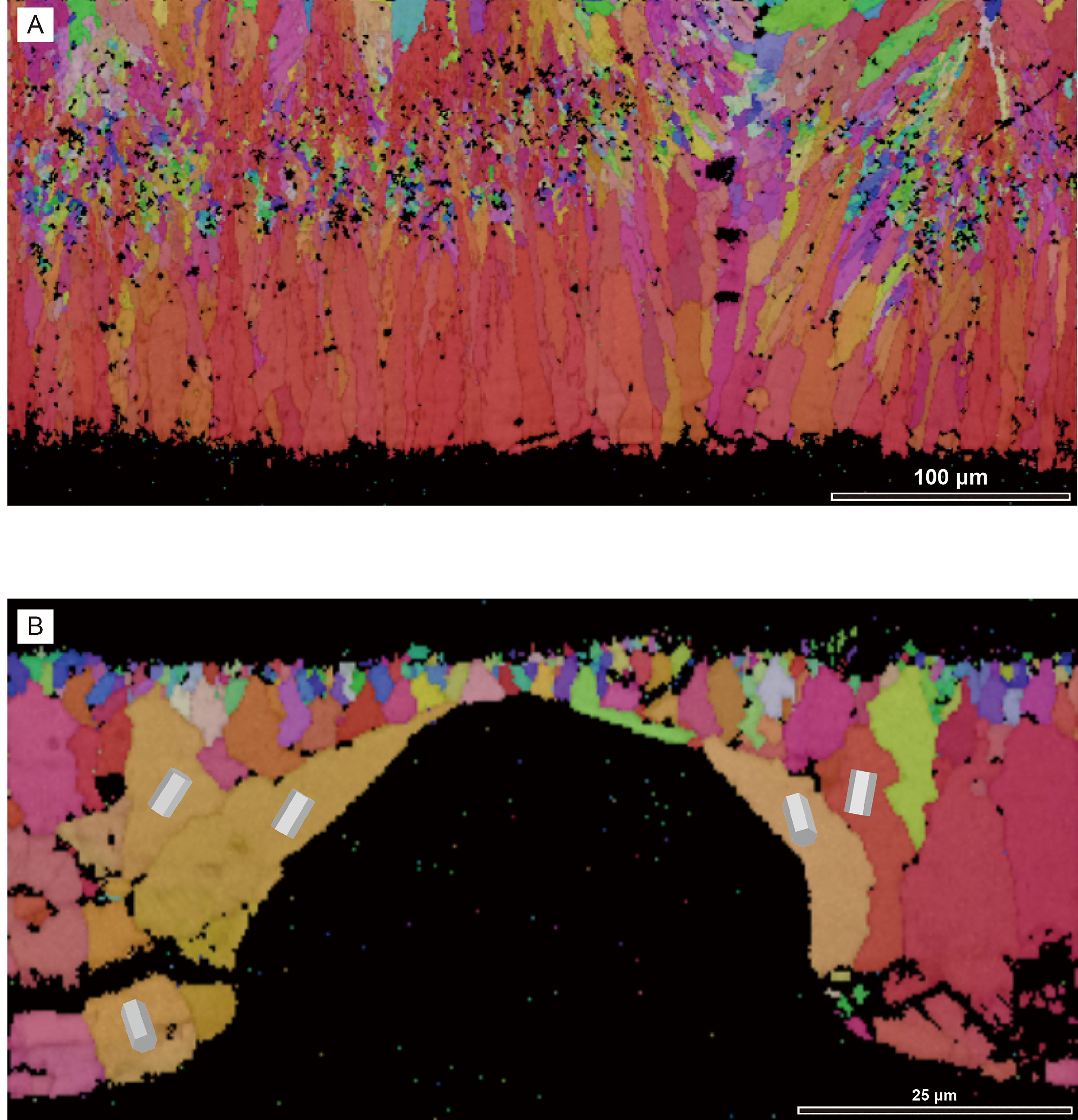

Supplement: S19 Fig — The colors represent the same crystallographic information as in Fig 9. The hexagonal columns in the IPF map show the main direction of c-axis. Note that a- and b-axes were not considered. Outside of eggshell is up. (A) Gekko gecko. Note the small and randomly oriented grains in the middle layer. (B) Paroedura pictus. The EBSD analysis was conducted on the chamber-like structure. The result showed that the columns in the chamber-like structure converge to the top of the chamber which is different from the typical c-axis direction of calcite grains in the main eggshell. (TIF) [file pone.0199496.s021.tif]
