# Supplementary material for: A comparative study of eggshells of Gekkota with morphological, chemical compositional and crystallographic approaches and its evolutionary implications
Source: PLoS One. 2018 Jun 22;13(6):e0199496. doi: 10.1371/journal.pone.0199496 (PMC6014675; doi:10.1371/journal.pone.0199496)
Supplement: S16 Fig — Note the high weight percentage of P in the residual materials of the blocky layer. (PDF) [file pone.0199496.s018.pdf]

Finding F

12/19/2017

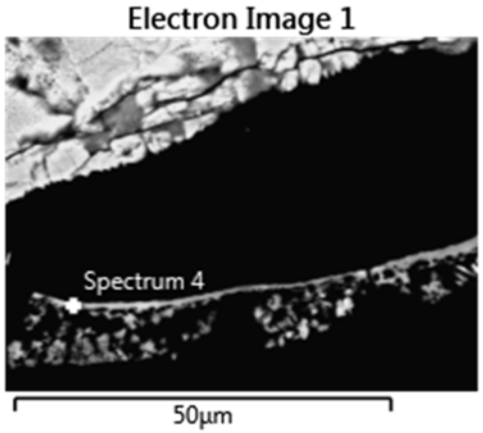

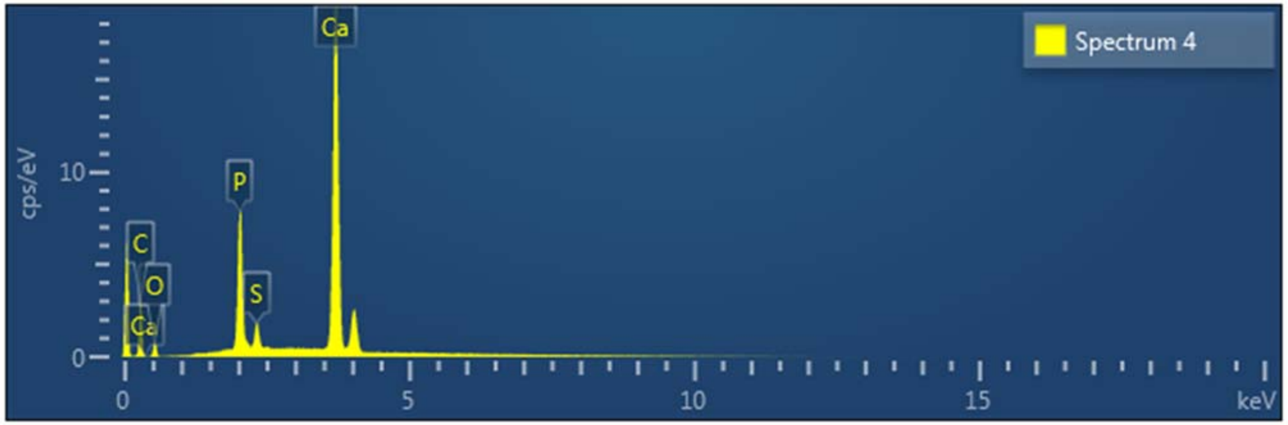

Element	Line Type	Apparent Concentration	k Ratio	Wt%	Wt% Sigma	Standard Label	Factory Standard	Standard Calibration Date
C	K series	0.33	0.00335	21.26	0.30	C Vit	Yes	
O	K series	0.16	0.00052	8.38	0.25	SiO2	Yes	
P	K series	1.04	0.00582	11.40	0.11	GaP	Yes	
S	K series	0.14	0.00123	2.41	0.07	FeS2	Yes	
Ca	K series	3.47	0.03098	56.55	0.29	Wollastonite	Yes	
Total:				100.00				

OXFORD INSTRUMENTS
The Business of Science®

Finding F

12/19/2017

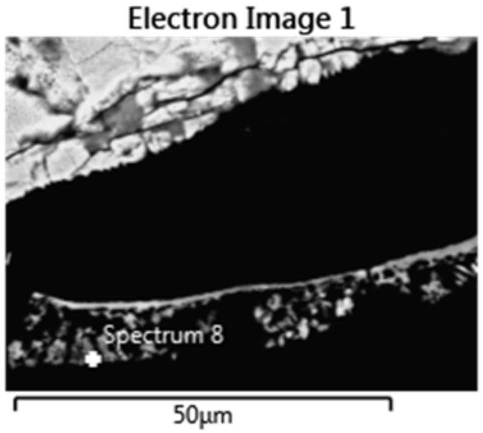

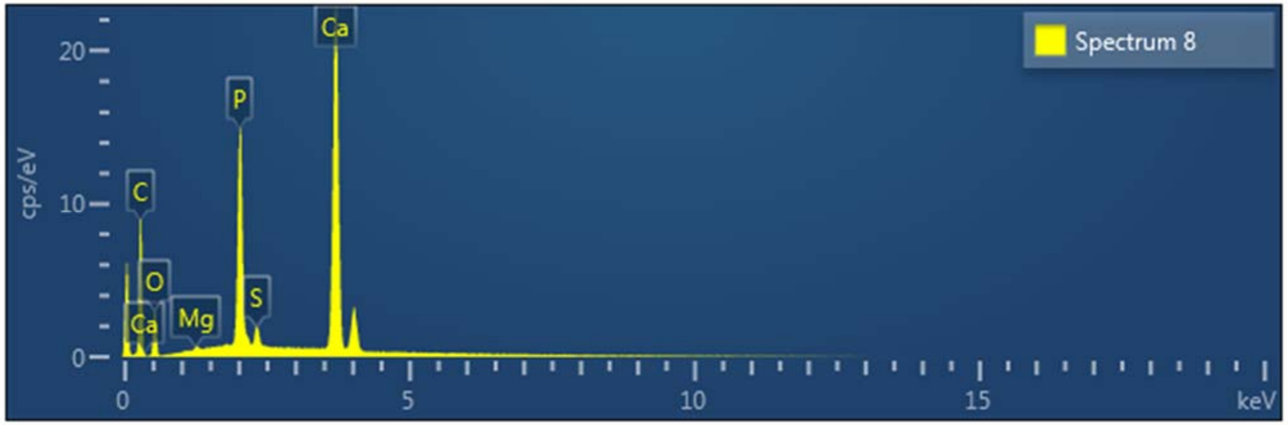

Element	Line Type	Apparent Concentration	k Ratio	Wt%	Wt% Sigma	Standard Label	Factory Standard	Standard Calibration Date
C	K series	0.97	0.00974	33.34	0.31	C Vit	Yes	
O	K series	0.56	0.00189	14.39	0.27	SiO2	Yes	
Mg	K series	0.02	0.00012	0.19	0.03	MgO	Yes	
P	K series	1.97	0.01104	12.07	0.11	GaP	Yes	
S	K series	0.14	0.00118	1.31	0.05	FeS2	Yes	
Ca	K series	4.20	0.03749	38.70	0.24	Wollastonite	Yes	
Total:				100.00				

Finding F

12/19/2017

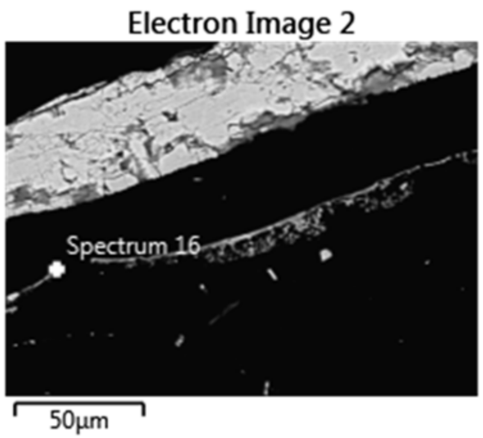

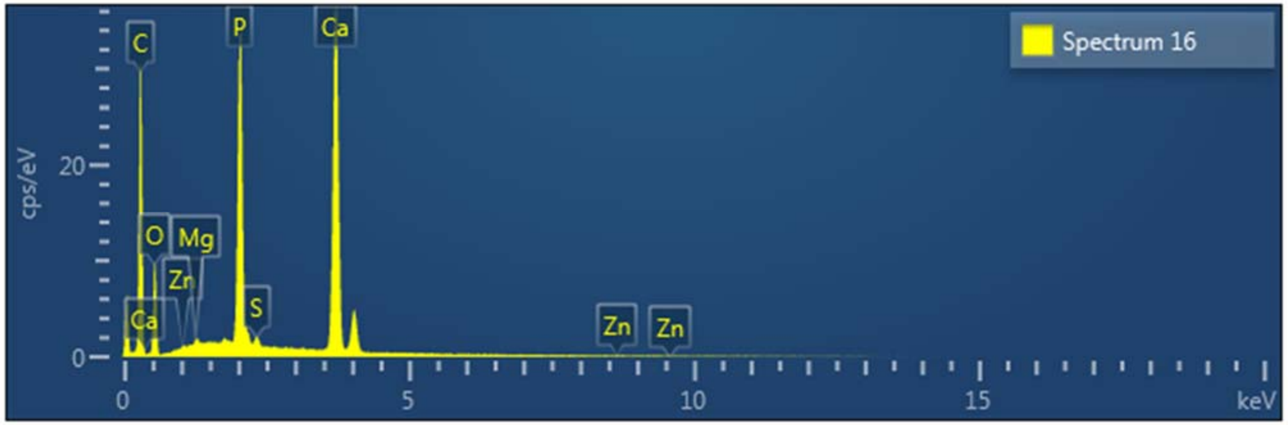

Element	Line Type	Apparent Concentration	k Ratio	Wt%	Wt% Sigma	Standard Label	Factory Standard	Standard Calibration Date
C	K series	3.21	0.03212	43.10	0.29	C Vit	Yes	
O	K series	1.82	0.00612	17.68	0.26	SiO2	Yes	
Mg	K series	0.05	0.00030	0.20	0.03	MgO	Yes	
P	K series	4.50	0.02515	11.76	0.11	GaP	Yes	
S	K series	0.10	0.00088	0.42	0.04	FeS2	Yes	
Ca	K series	6.71	0.05996	26.63	0.18	Wollastonite	Yes	
Zn	L series	0.02	0.00025	0.21	0.07	Zn	Yes	
Total:				100.00				

Finding F

12/19/2017

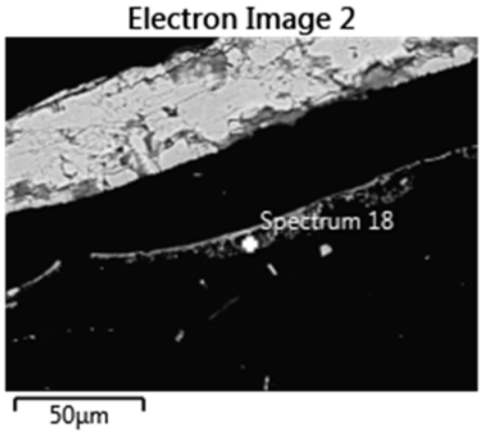

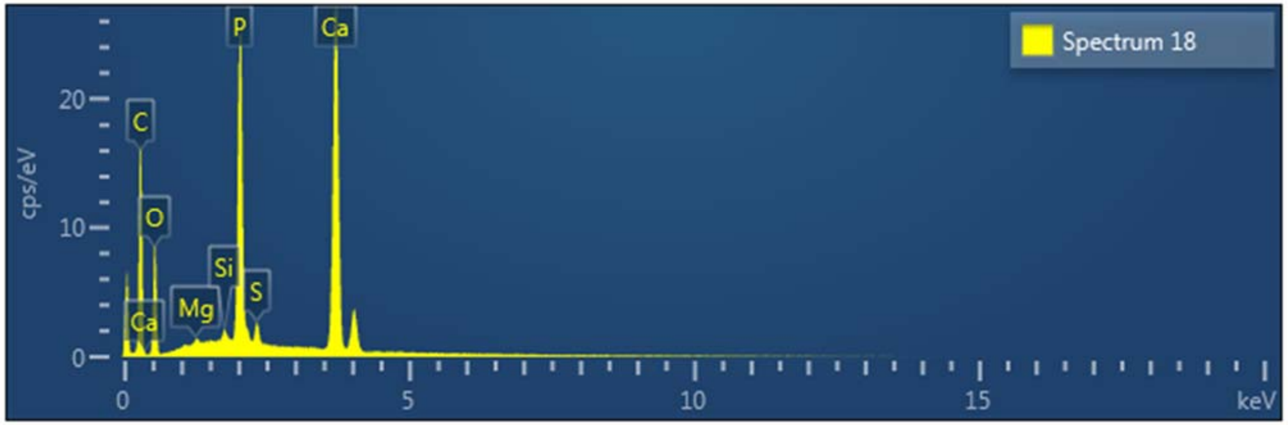

Element	Line Type	Apparent Concentration	k Ratio	Wt%	Wt% Sigma	Standard Label	Factory Standard	Standard Calibration Date
C	K series	1.75	0.01755	36.69	0.31	C Vit	Yes	
O	K series	1.60	0.00539	21.38	0.28	SiO2	Yes	
Mg	K series	0.03	0.00020	0.19	0.03	MgO	Yes	
Si	K series	0.06	0.00044	0.30	0.03	SiO2	Yes	
P	K series	3.33	0.01861	12.39	0.11	GaP	Yes	
S	K series	0.17	0.00147	1.00	0.05	FeS2	Yes	
Ca	K series	4.98	0.04453	28.05	0.20	Wollastonite	Yes	
Total:				100.00				

OXFORD INSTRUMENTS
The Business of Science®

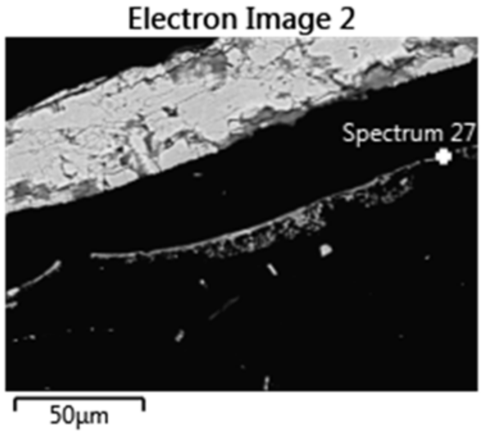

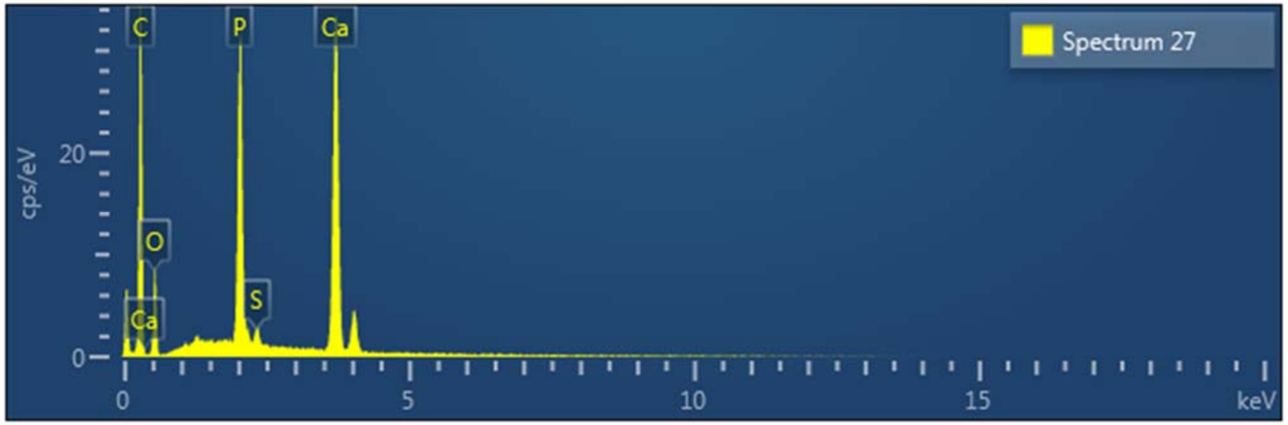

Element	Line Type	Apparent Concentration	k Ratio	Wt%	Wt% Sigma	Standard Label	Factory Standard	Standard Calibration Date
C	K series	3.64	0.03638	47.51	0.44	C Vit	Yes	
O	K series	1.58	0.00532	15.81	0.40	SiO2	Yes	
P	K series	4.16	0.02325	11.01	0.16	GaP	Yes	
S	K series	0.18	0.00152	0.73	0.06	FeS2	Yes	
Ca	K series	6.17	0.05516	24.94	0.28	Wollastonite	Yes	
Total:				100.00				

Finding F

12/19/2017

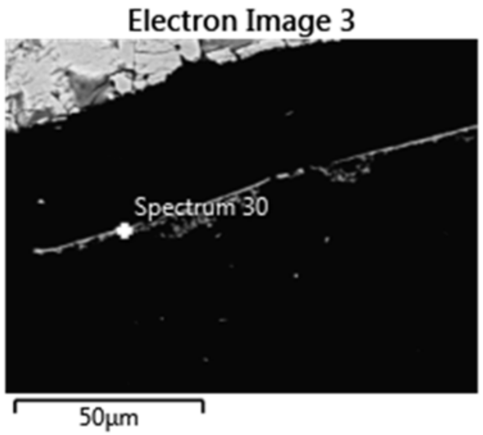

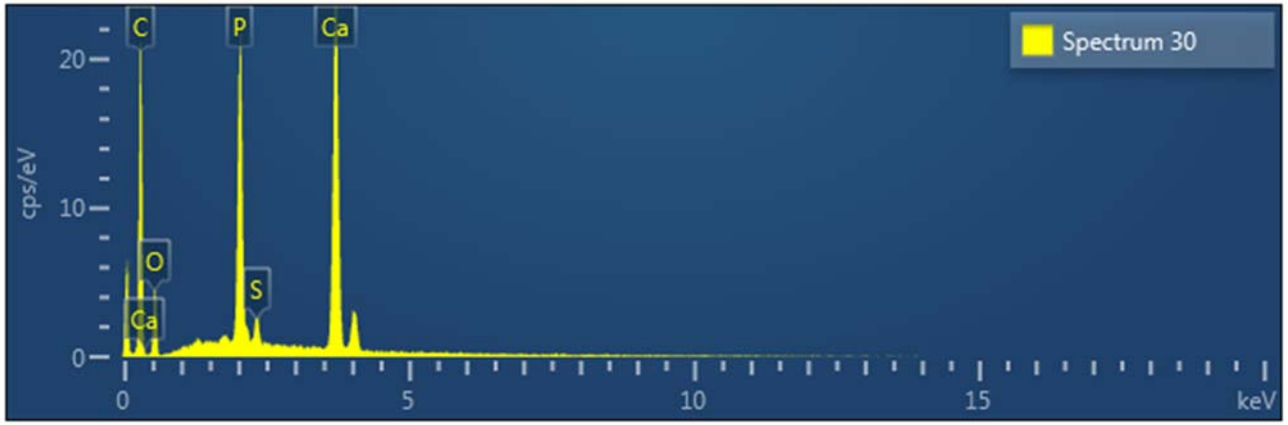

Element	Line Type	Apparent Concentration	k Ratio	Wt%	Wt% Sigma	Standard Label	Factory Standard	Standard Calibration Date
C	K series	2.23	0.02231	47.06	0.49	C Vit	Yes	
O	K series	0.85	0.00285	13.58	0.43	SiO2	Yes	
P	K series	2.76	0.01542	11.26	0.18	GaP	Yes	
S	K series	0.20	0.00169	1.26	0.08	FeS2	Yes	
Ca	K series	4.29	0.03837	26.84	0.32	Wollastonite	Yes	
Total:				100.00				

Finding F

12/19/2017

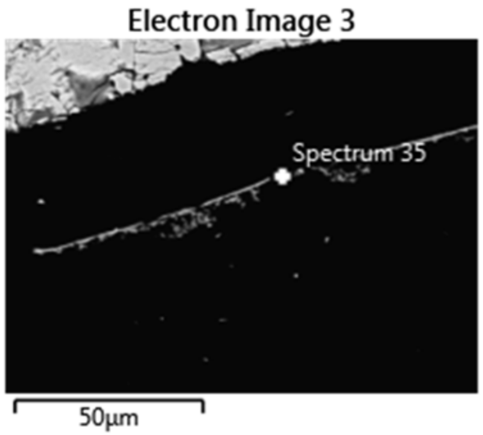

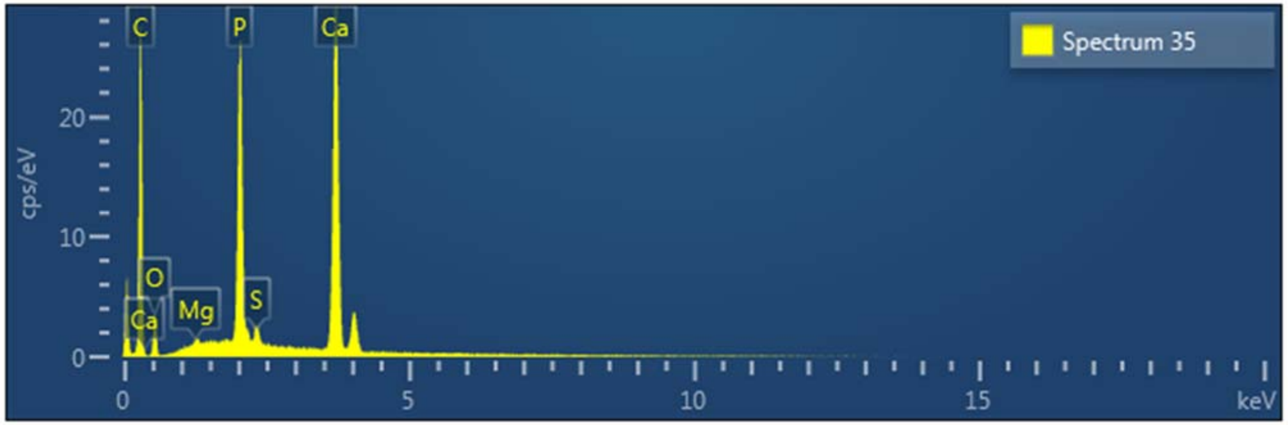

Element	Line Type	Apparent Concentration	k Ratio	Wt%	Wt% Sigma	Standard Label	Factory Standard	Standard Calibration Date
C	K series	2.89	0.02893	50.22	0.41	C Vit	Yes	
O	K series	0.66	0.00222	9.29	0.33	SiO2	Yes	
Mg	K series	0.03	0.00021	0.17	0.05	MgO	Yes	
P	K series	3.43	0.01919	11.69	0.16	GaP	Yes	
S	K series	0.16	0.00137	0.86	0.07	FeS2	Yes	
Ca	K series	5.31	0.04747	27.77	0.28	Wollastonite	Yes	
Total:				100.00				

OXFORD INSTRUMENTS
The Business of Science®

Finding F

12/19/2017

Electron Image 3

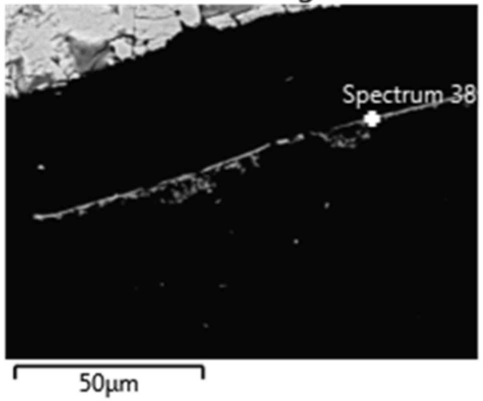

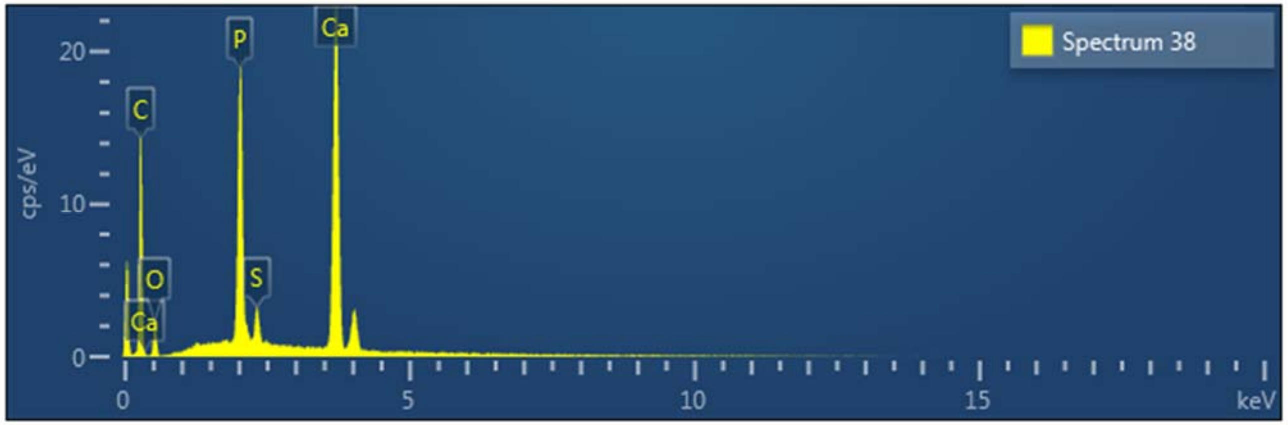

Element	Line Type	Apparent Concentration	k Ratio	Wt%	Wt% Sigma	Standard Label	Factory Standard	Standard Calibration Date
C	K series	1.56	0.01557	44.18	0.49	C Vit	Yes	
O	K series	0.44	0.00147	9.27	0.37	SiO2	Yes	
P	K series	2.49	0.01390	12.40	0.19	GaP	Yes	
S	K series	0.25	0.00211	1.94	0.09	FeS2	Yes	
Ca	K series	4.21	0.03762	32.22	0.35	Wollastonite	Yes	
Total:				100.00				

OXFORD INSTRUMENTS
The Business of Science®

Finding F

12/19/2017

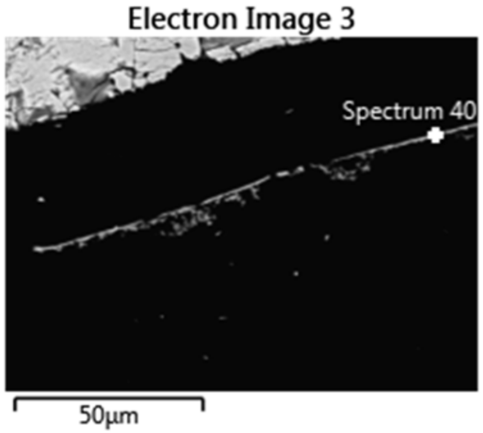

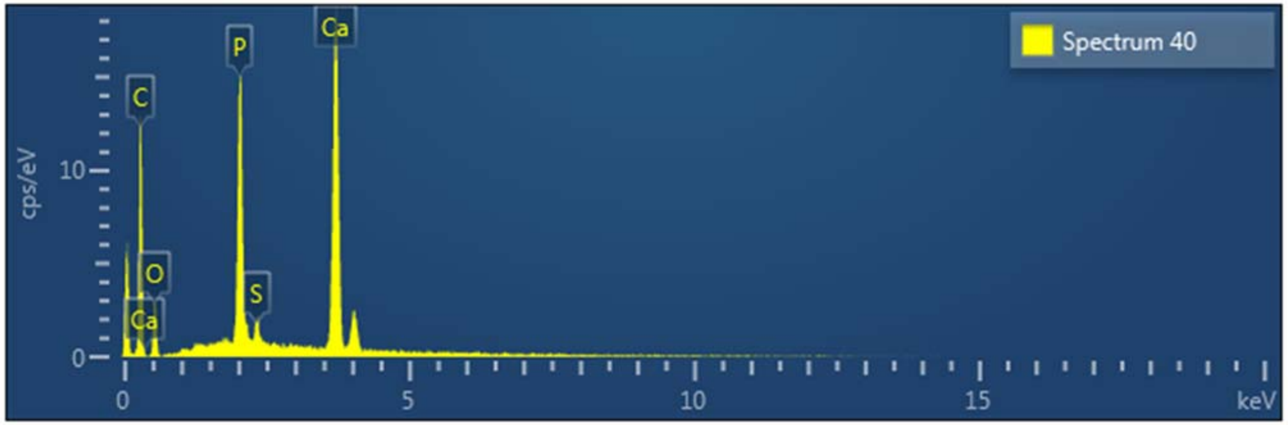

Element	Line Type	Apparent Concentration	k Ratio	Wt%	Wt% Sigma	Standard Label	Factory Standard	Standard Calibration Date
C	K series	1.34	0.01342	42.80	0.76	C Vit	Yes	
O	K series	0.57	0.00191	13.63	0.64	SiO2	Yes	
P	K series	1.96	0.01096	11.72	0.28	GaP	Yes	
S	K series	0.13	0.00114	1.25	0.13	FeS2	Yes	
Ca	K series	3.36	0.03001	30.61	0.53	Wollastonite	Yes	
Total:				100.00				

Finding F

12/19/2017

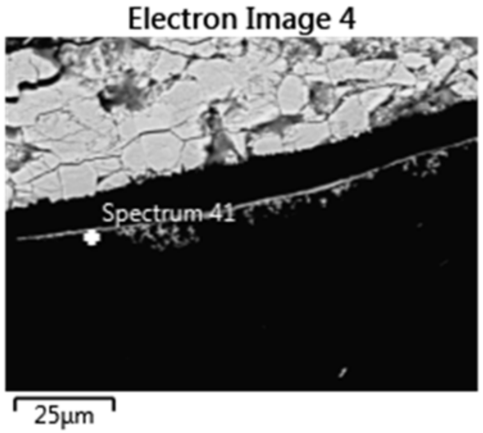

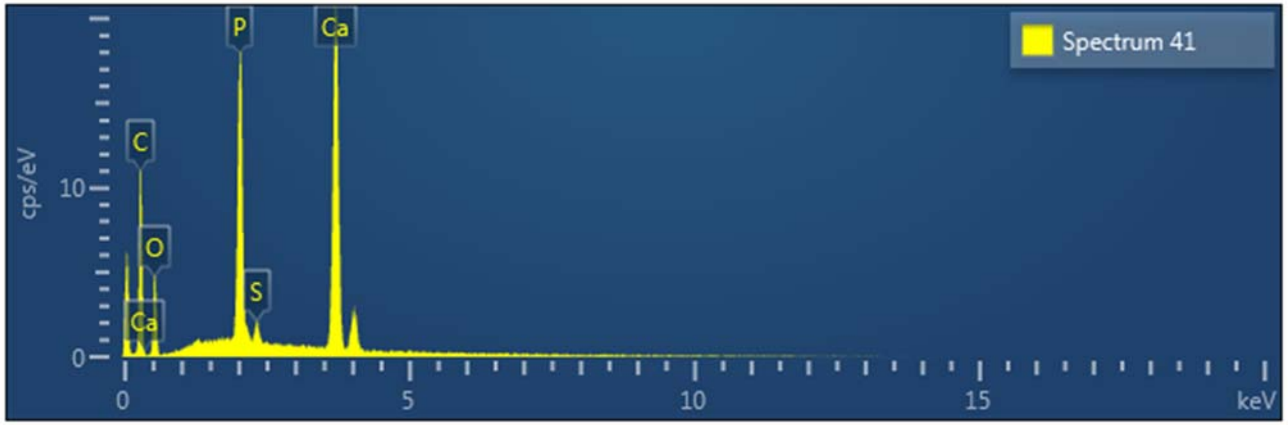

Element	Line Type	Apparent Concentration	k Ratio	Wt%	Wt% Sigma	Standard Label	Factory Standard	Standard Calibration Date
C	K series	1.16	0.01159	35.97	0.63	C Vit	Yes	
O	K series	0.90	0.00301	18.63	0.56	SiO2	Yes	
P	K series	2.35	0.01316	12.84	0.24	GaP	Yes	
S	K series	0.13	0.00110	1.10	0.10	FeS2	Yes	
Ca	K series	3.80	0.03397	31.46	0.43	Wollastonite	Yes	
Total:				100.00				

OXFORD INSTRUMENTS
The Business of Science®

Finding F

12/19/2017

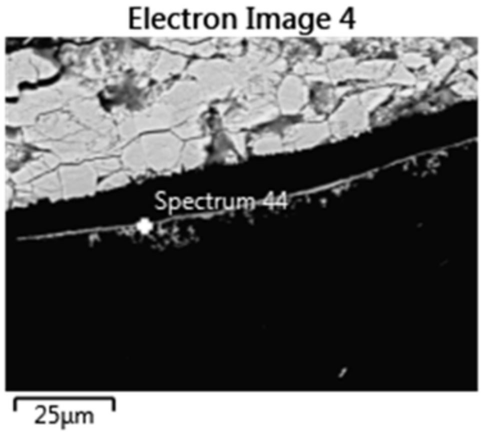

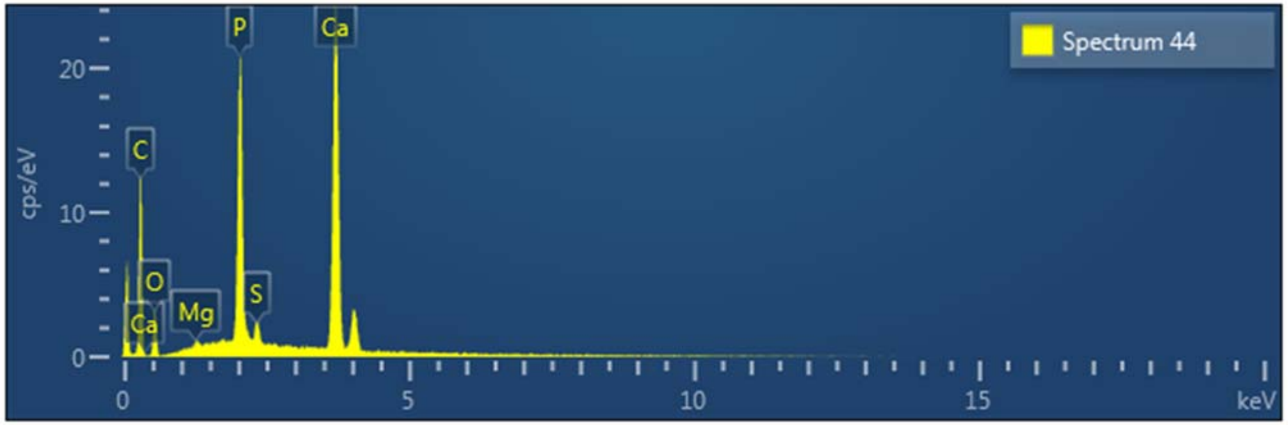

Element	Line Type	Apparent Concentration	k Ratio	Wt%	Wt% Sigma	Standard Label	Factory Standard	Standard Calibration Date
C	K series	1.35	0.01345	38.80	0.53	C Vit	Yes	
O	K series	0.62	0.00209	12.74	0.43	SiO2	Yes	
Mg	K series	0.03	0.00021	0.26	0.06	MgO	Yes	
P	K series	2.67	0.01491	13.28	0.20	GaP	Yes	
S	K series	0.15	0.00133	1.22	0.09	FeS2	Yes	
Ca	K series	4.44	0.03971	33.70	0.38	Wollastonite	Yes	
Total:				100.00				

OXFORD INSTRUMENTS
The Business of Science®

Finding F

12/19/2017

Electron Image 4

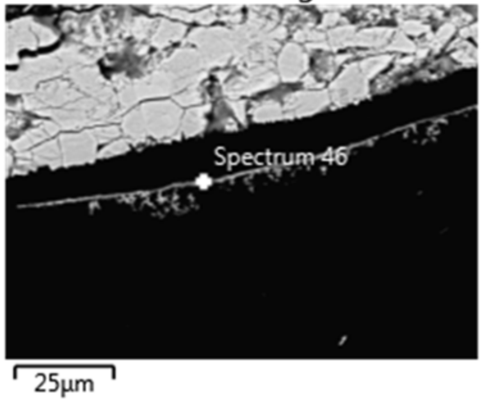

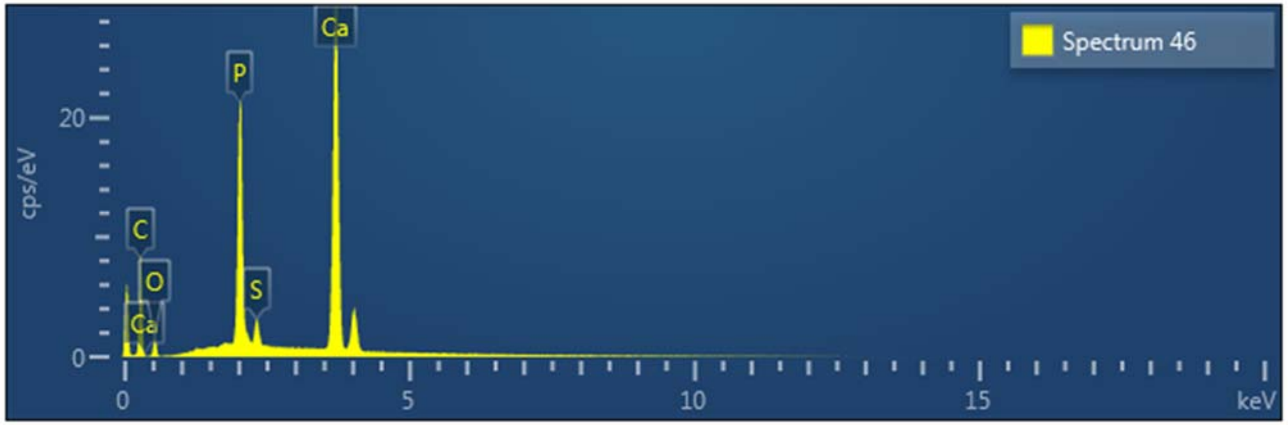

Element	Line Type	Apparent Concentration	k Ratio	Wt%	Wt% Sigma	Standard Label	Factory Standard	Standard Calibration Date
C	K series	0.89	0.00889	31.34	0.33	C Vit	Yes	
O	K series	0.30	0.00101	7.44	0.23	SiO2	Yes	
P	K series	2.83	0.01582	15.19	0.13	GaP	Yes	
S	K series	0.22	0.00193	1.93	0.06	FeS2	Yes	
Ca	K series	5.39	0.04814	44.10	0.27	Wollastonite	Yes	
Total:				100.00				

OXFORD INSTRUMENTS
The Business of Science®
