# Supplementary material for: A comparative study of eggshells of Gekkota with morphological, chemical compositional and crystallographic approaches and its evolutionary implications
Source: PLoS One. 2018 Jun 22;13(6):e0199496. doi: 10.1371/journal.pone.0199496 (PMC6014675; doi:10.1371/journal.pone.0199496)
Supplement: S17 Fig — Note the high weight percentage of P and the presence of F and Cl in the residual materials of the blocky layer. (PDF) [file pone.0199496.s019.pdf]

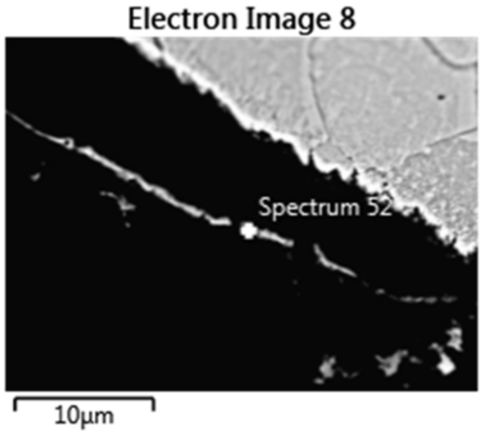

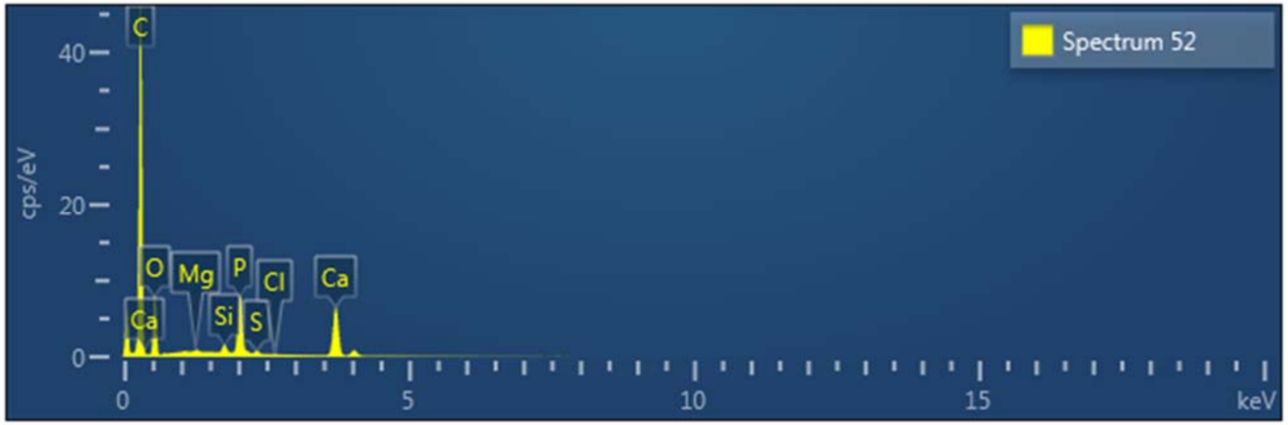

Element	Line Type	Apparent Concentration	k Ratio	Wt%	Wt% Sigma	Standard Label	Factory Standard	Standard Calibration Date
C	K series	0.73	0.00731	59.05	0.13	C Vit	Yes	
O	K series	0.26	0.00087	14.80	0.10	SiO2	Yes	
Mg	K series	0.00	0.00003	0.17	0.02	MgO	Yes	
Si	K series	0.02	0.00016	0.78	0.02	SiO2	Yes	
P	K series	0.26	0.00144	7.20	0.05	GaP	Yes	
S	K series	0.01	0.00010	0.48	0.02	FeS2	Yes	
Cl	K series	0.00	0.00001	0.07	0.02	NaCl	Yes	
Ca	K series	0.39	0.00351	17.45	0.09	Wollastonite	Yes	
Total:				100.00				

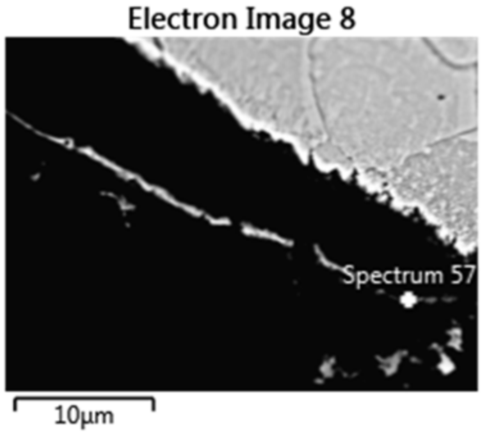

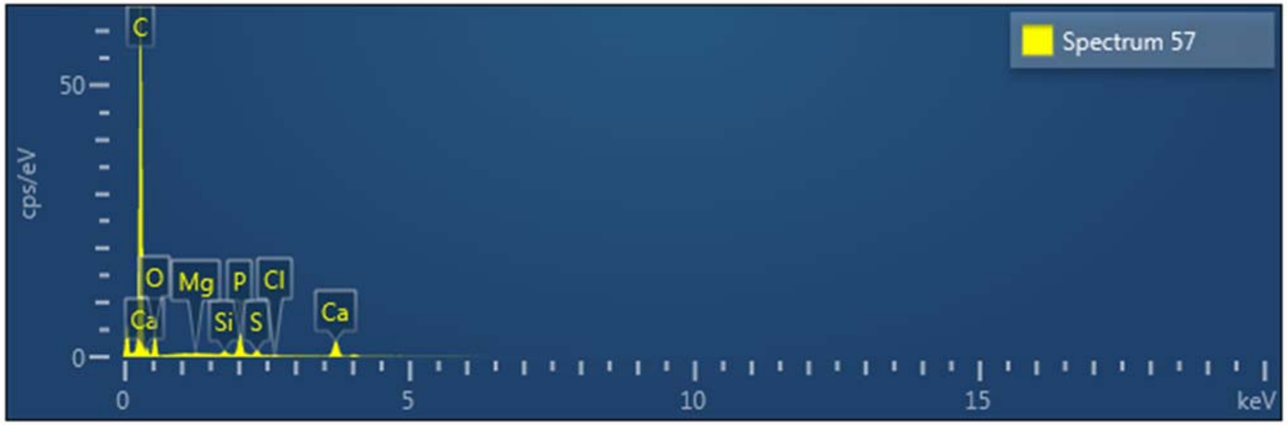

Element	Line Type	Apparent Concentration	k Ratio	Wt%	Wt% Sigma	Standard Label	Factory Standard	Standard Calibration Date
C	K series	1.01	0.01007	75.37	0.12	C Vit	Yes	
O	K series	0.16	0.00053	9.87	0.10	SiO2	Yes	
Mg	K series	0.00	0.00002	0.11	0.02	MgO	Yes	
Si	K series	0.01	0.00009	0.50	0.02	SiO2	Yes	
P	K series	0.13	0.00074	4.09	0.04	GaP	Yes	
S	K series	0.03	0.00023	1.22	0.03	FeS2	Yes	
Cl	K series	0.00	0.00004	0.23	0.02	NaCl	Yes	
Ca	K series	0.17	0.00156	8.62	0.08	Wollastonite	Yes	
Total:				100.00				

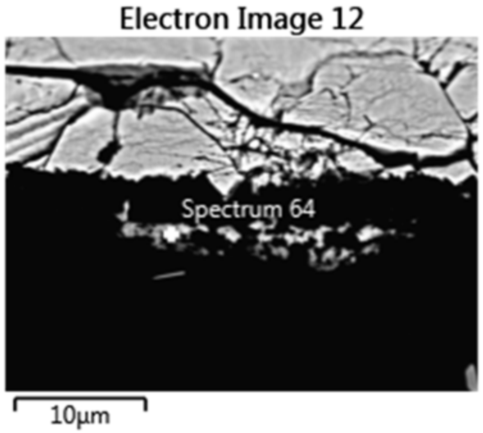

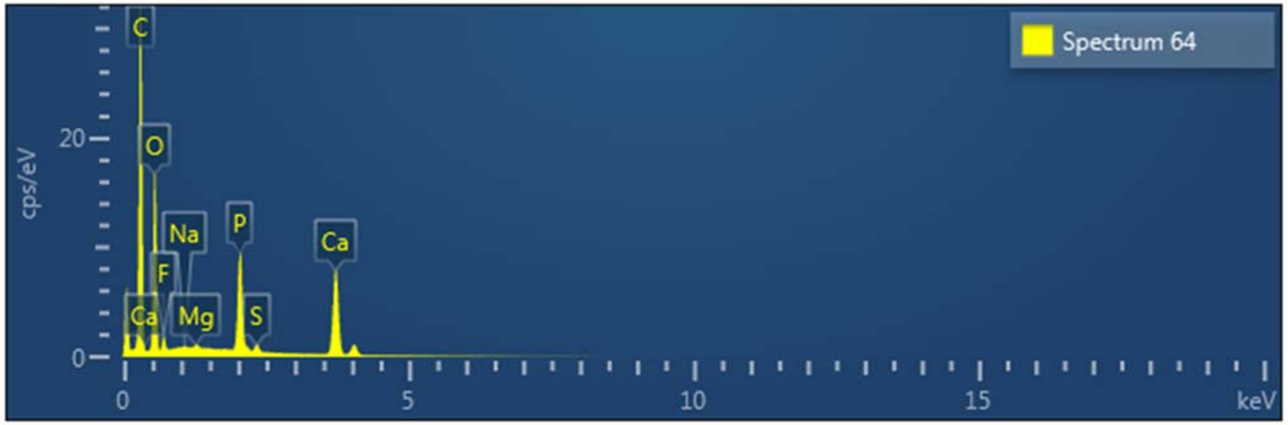

Element	Line Type	Apparent Concentration	k Ratio	Wt%	Wt% Sigma	Standard Label	Factory Standard	Standard Calibration Date
C	K series	0.48	0.00476	41.36	0.13	C Vit	Yes	
O	K series	0.55	0.00185	26.84	0.12	SiO2	Yes	
F	K series	0.08	0.00016	2.14	0.06	CaF2	Yes	
Na	K series	0.00	0.00001	0.08	0.02	Albite	Yes	
Mg	K series	0.01	0.00004	0.20	0.02	MgO	Yes	
P	K series	0.31	0.00172	8.29	0.05	GaP	Yes	
S	K series	0.02	0.00016	0.77	0.03	FeS2	Yes	
Ca	K series	0.48	0.00427	20.30	0.10	Wollastonite	Yes	
Total:				100.00				

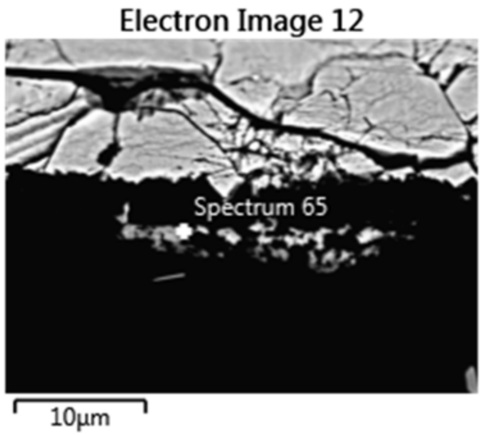

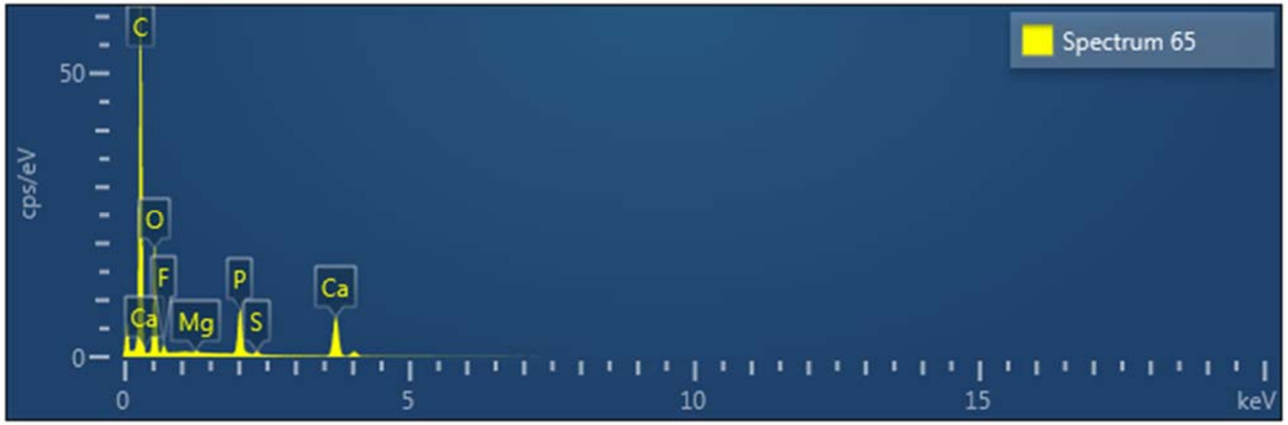

Element	Line Type	Apparent Concentration	k Ratio	Wt%	Wt% Sigma	Standard Label	Factory Standard	Standard Calibration Date
C	K series	0.92	0.00919	53.20	0.11	C Vit	Yes	
O	K series	0.63	0.00213	24.21	0.10	SiO2	Yes	
F	K series	0.09	0.00017	1.78	0.05	CaF2	Yes	
Mg	K series	0.00	0.00003	0.14	0.01	MgO	Yes	
P	K series	0.27	0.00152	5.80	0.04	GaP	Yes	
S	K series	0.02	0.00013	0.50	0.02	FeS2	Yes	
Ca	K series	0.42	0.00380	14.36	0.08	Wollastonite	Yes	
Total:				100.00				

OXFORD INSTRUMENTS
The Business of Science®

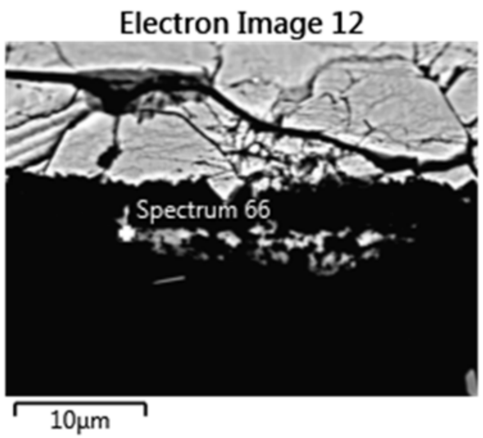

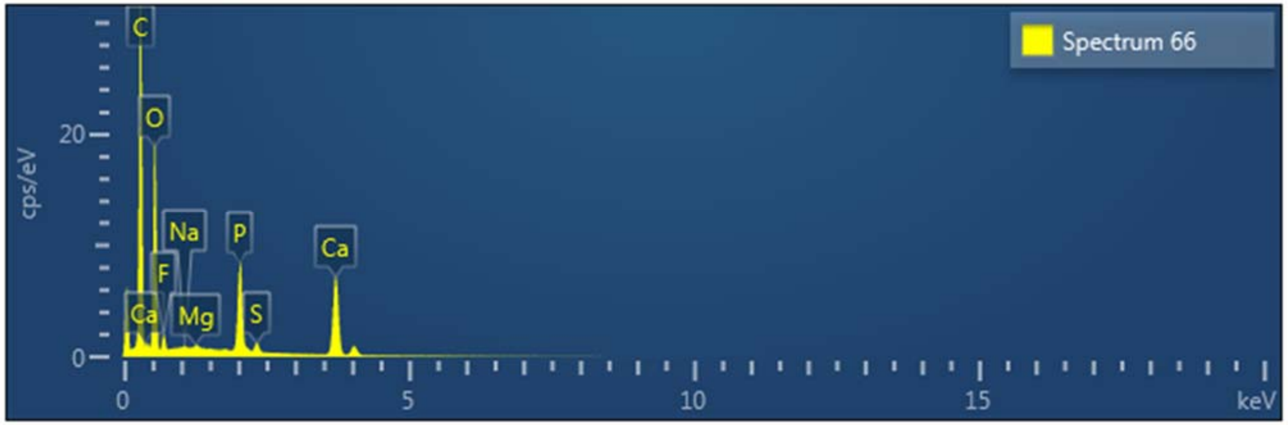

Element	Line Type	Apparent Concentration	k Ratio	Wt%	Wt% Sigma	Standard Label	Factory Standard	Standard Calibration Date
C	K series	0.46	0.00459	40.12	0.13	C Vit	Yes	
O	K series	0.63	0.00211	29.91	0.12	SiO2	Yes	
F	K series	0.09	0.00017	2.41	0.06	CaF2	Yes	
Na	K series	0.00	0.00002	0.12	0.02	Albite	Yes	
Mg	K series	0.00	0.00003	0.17	0.02	MgO	Yes	
P	K series	0.27	0.00151	7.48	0.05	GaP	Yes	
S	K series	0.02	0.00019	0.93	0.03	FeS2	Yes	
Ca	K series	0.43	0.00388	18.87	0.10	Wollastonite	Yes	
Total:				100.00				

OXFORD INSTRUMENTS
The Business of Science®

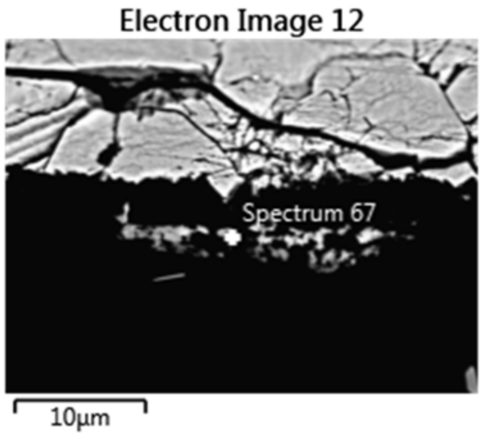

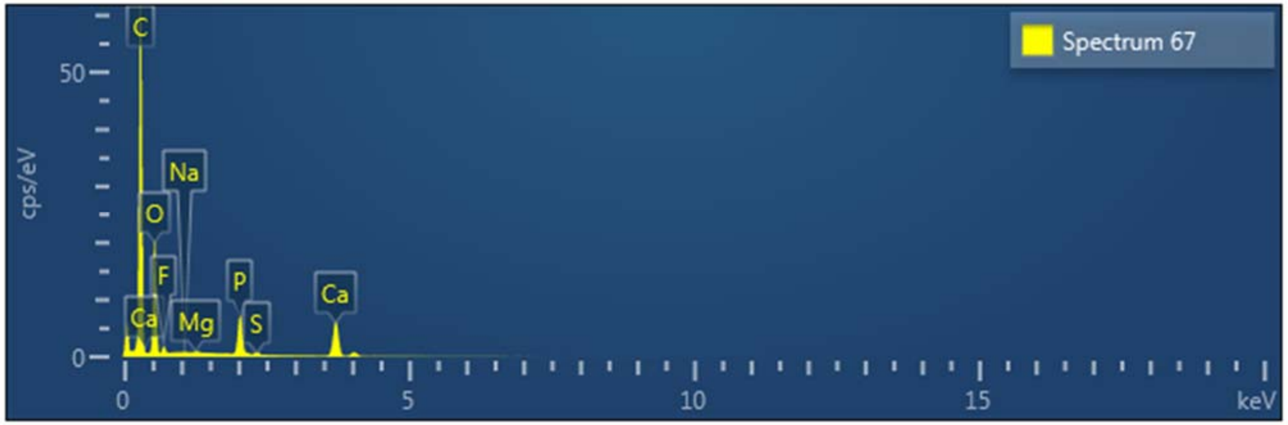

Element	Line Type	Apparent Concentration	k Ratio	Wt%	Wt% Sigma	Standard Label	Factory Standard	Standard Calibration Date
C	K series	0.92	0.00918	53.66	0.11	C Vit	Yes	
O	K series	0.66	0.00222	25.73	0.11	SiO2	Yes	
F	K series	0.08	0.00015	1.65	0.05	CaF2	Yes	
Na	K series	0.00	0.00001	0.05	0.02	Albite	Yes	
Mg	K series	0.00	0.00003	0.12	0.01	MgO	Yes	
P	K series	0.23	0.00131	5.25	0.04	GaP	Yes	
S	K series	0.01	0.00010	0.40	0.02	FeS2	Yes	
Ca	K series	0.37	0.00333	13.13	0.07	Wollastonite	Yes	
Total:				100.00				

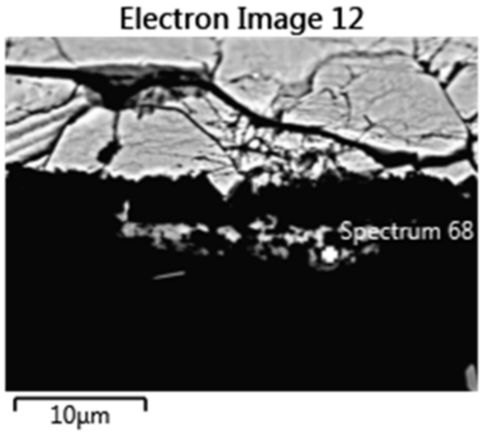

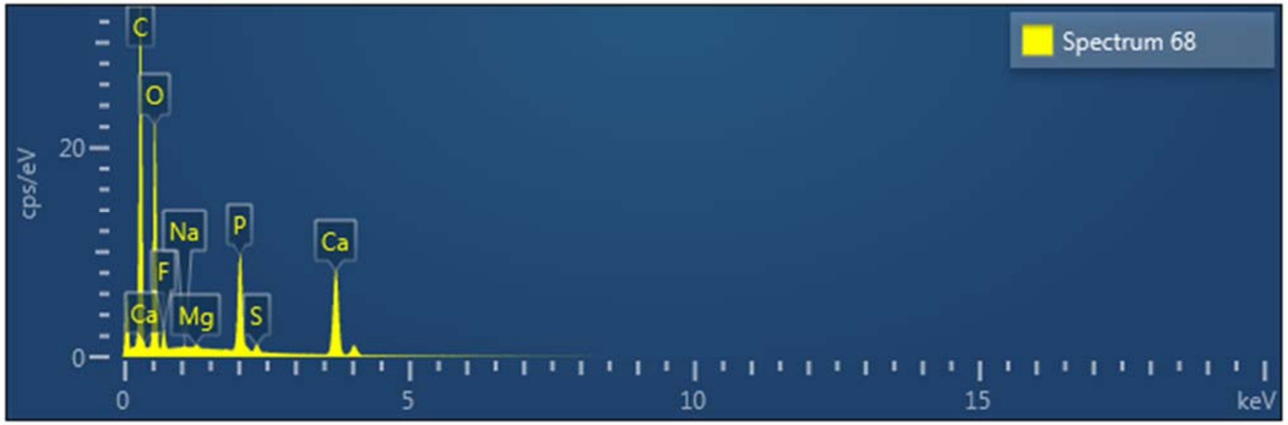

Element	Line Type	Apparent Concentration	k Ratio	Wt%	Wt% Sigma	Standard Label	Factory Standard	Standard Calibration Date
C	K series	0.49	0.00492	38.58	0.12	C Vit	Yes	
O	K series	0.73	0.00247	30.73	0.12	SiO2	Yes	
F	K series	0.11	0.00023	2.80	0.06	CaF2	Yes	
Na	K series	0.00	0.00001	0.08	0.02	Albite	Yes	
Mg	K series	0.01	0.00004	0.19	0.02	MgO	Yes	
P	K series	0.32	0.00177	7.78	0.05	GaP	Yes	
S	K series	0.02	0.00018	0.77	0.02	FeS2	Yes	
Ca	K series	0.50	0.00442	19.08	0.09	Wollastonite	Yes	
Total:				100.00				

OXFORD INSTRUMENTS
The Business of Science®

Electron Image 12

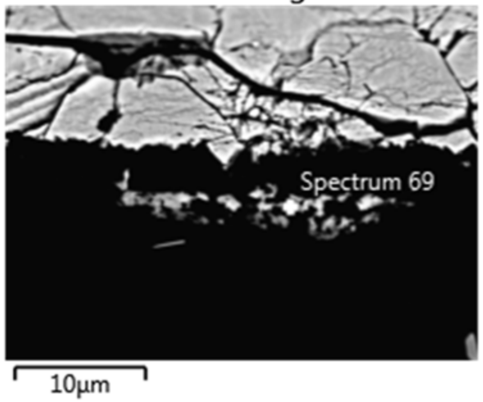

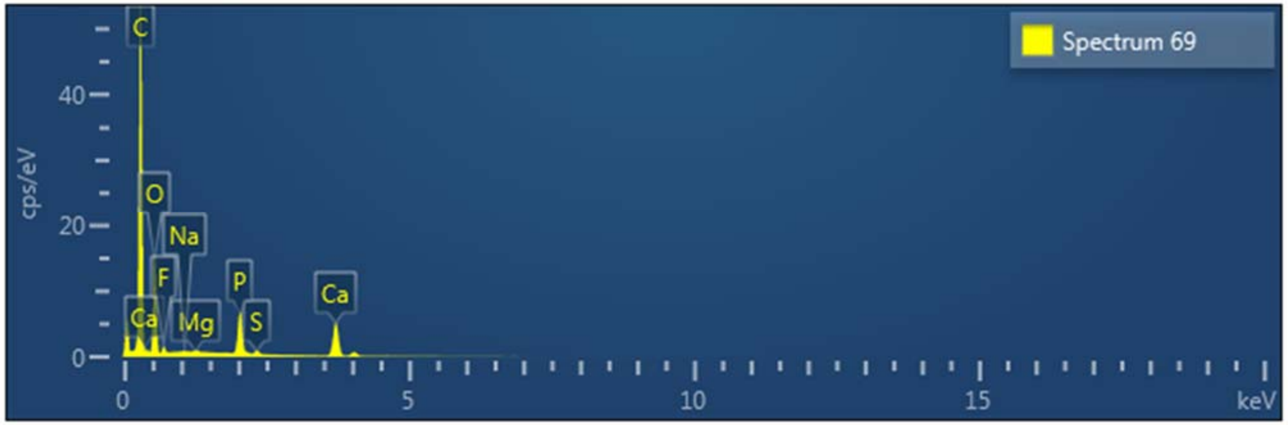

Element	Line Type	Apparent Concentration	k Ratio	Wt%	Wt% Sigma	Standard Label	Factory Standard	Standard Calibration Date
C	K series	0.79	0.00790	55.80	0.13	C Vit	Yes	
O	K series	0.50	0.00167	23.34	0.11	SiO2	Yes	
F	K series	0.06	0.00012	1.49	0.05	CaF2	Yes	
Na	K series	0.00	0.00001	0.07	0.02	Albite	Yes	
Mg	K series	0.00	0.00003	0.15	0.02	MgO	Yes	
P	K series	0.22	0.00122	5.72	0.04	GaP	Yes	
S	K series	0.01	0.00013	0.58	0.02	FeS2	Yes	
Ca	K series	0.31	0.00275	12.84	0.08	Wollastonite	Yes	
Total:				100.00				

OXFORD INSTRUMENTS
The Business of Science®

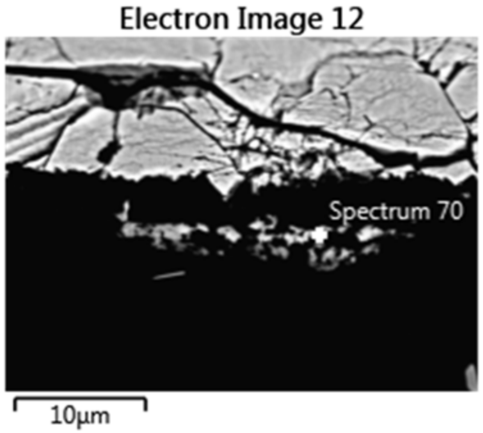

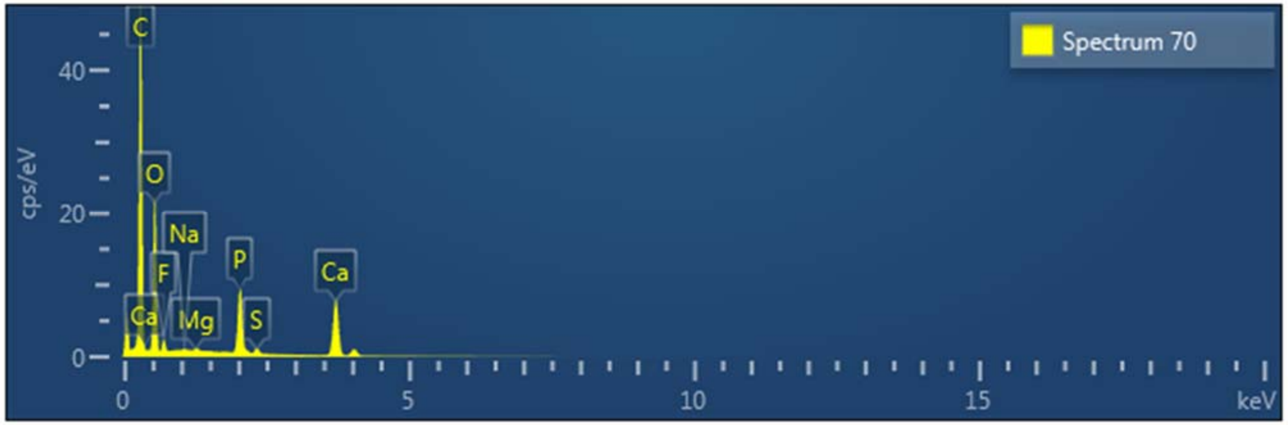

Element	Line Type	Apparent Concentration	k Ratio	Wt%	Wt% Sigma	Standard Label	Factory Standard	Standard Calibration Date
C	K series	0.73	0.00727	46.57	0.12	C Vit	Yes	
O	K series	0.71	0.00240	27.26	0.11	SiO2	Yes	
F	K series	0.10	0.00020	2.19	0.05	CaF2	Yes	
Na	K series	0.00	0.00001	0.08	0.02	Albite	Yes	
Mg	K series	0.01	0.00004	0.17	0.02	MgO	Yes	
P	K series	0.31	0.00171	6.69	0.04	GaP	Yes	
S	K series	0.02	0.00018	0.67	0.02	FeS2	Yes	
Ca	K series	0.48	0.00425	16.37	0.08	Wollastonite	Yes	
Total:				100.00				

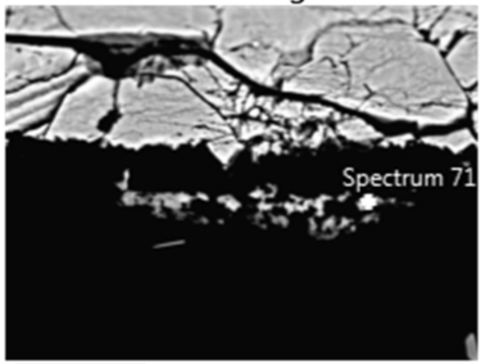

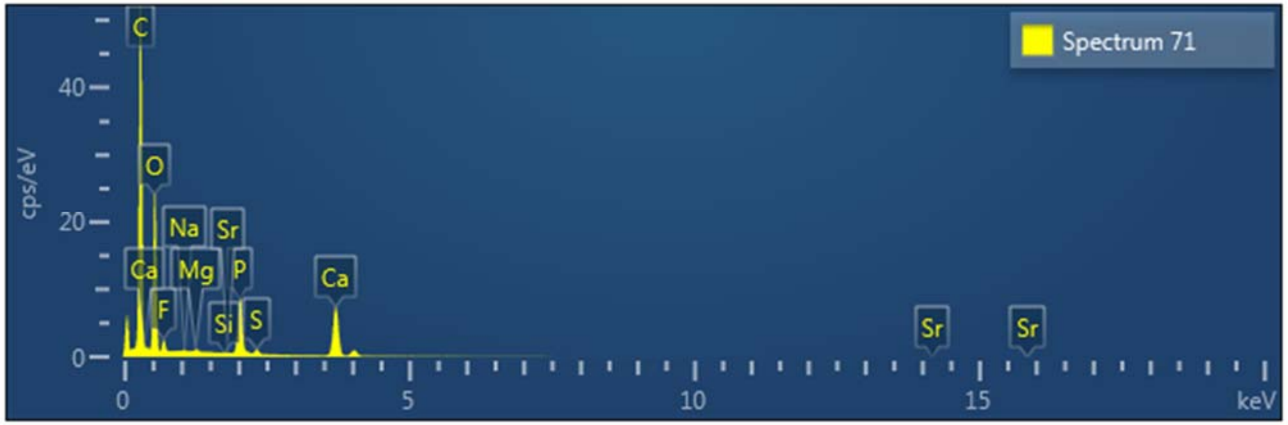

Element	Line Type	Apparent Concentration	k Ratio	Wt%	Wt% Sigma	Standard Label	Factory Standard	Standard Calibration Date
C	K series	0.78	0.00775	47.00	0.12	C Vit	Yes	
O	K series	0.80	0.00268	28.79	0.11	SiO2	Yes	
F	K series	0.11	0.00021	2.24	0.05	CaF2	Yes	
Na	K series	0.00	0.00001	0.08	0.02	Albite	Yes	
Mg	K series	0.01	0.00004	0.16	0.01	MgO	Yes	
Si	K series	0.00	0.00002	0.07	0.02	SiO2	Yes	
P	K series	0.28	0.00155	5.93	0.04	GaP	Yes	
S	K series	0.02	0.00016	0.61	0.02	FeS2	Yes	
Ca	K series	0.45	0.00398	14.95	0.08	Wollastonite	Yes	
Sr	L series	0.00	0.00004	0.18	0.05	SrF2	Yes	
Total:				100.00				

FSD Mixed Image 2

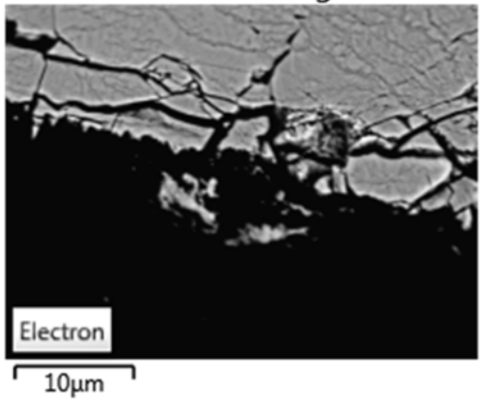

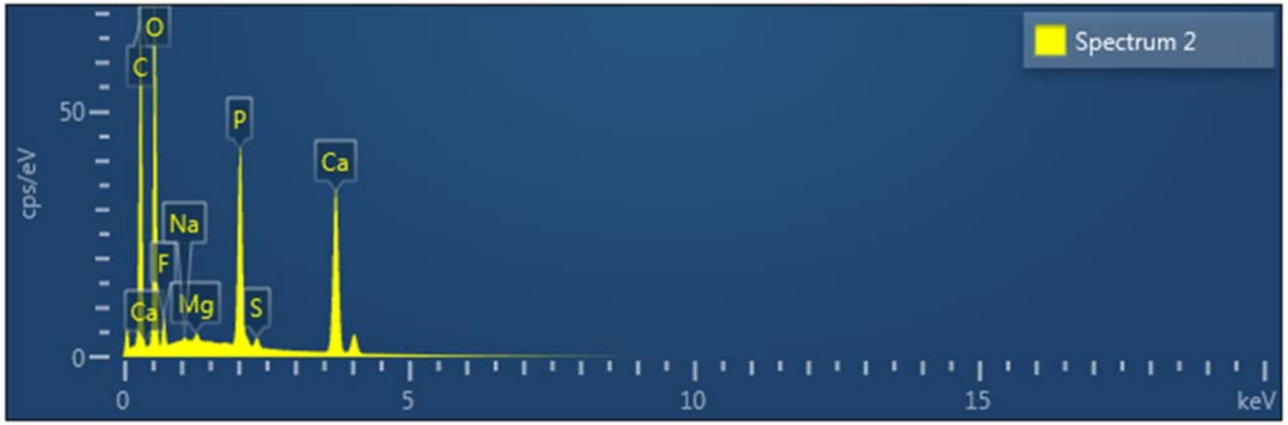

Element	Line Type	Apparent Concentration	k Ratio	Wt%	Wt% Sigma	Standard Label	Factory Standard	Standard Calibration Date
C	K series	1.04	0.01043	29.58	0.07	C Vit	Yes	
O	K series	2.28	0.00766	31.07	0.07	SiO2	Yes	
F	K series	0.36	0.00071	2.83	0.04	CaF2	Yes	
Na	K series	0.02	0.00006	0.13	0.01	Albite	Yes	
Mg	K series	0.03	0.00017	0.27	0.01	MgO	Yes	
P	K series	1.39	0.00776	10.69	0.03	GaP	Yes	
S	K series	0.06	0.00048	0.65	0.01	FeS2	Yes	
Ca	K series	2.05	0.01834	24.78	0.06	Wollastonite	Yes	
Total:				100.00				

OXFORD INSTRUMENTS
The Business of Science®
