# Supplementary material for: A comparative study of eggshells of Gekkota with morphological, chemical compositional and crystallographic approaches and its evolutionary implications
Source: PLoS One. 2018 Jun 22;13(6):e0199496. doi: 10.1371/journal.pone.0199496 (PMC6014675; doi:10.1371/journal.pone.0199496)
Supplement: S18 Fig — Note the high weight percentage of P and the presence of F and Cl in the blocky layer. (PDF) [file pone.0199496.s020.pdf]

Finding F

12/19/2017

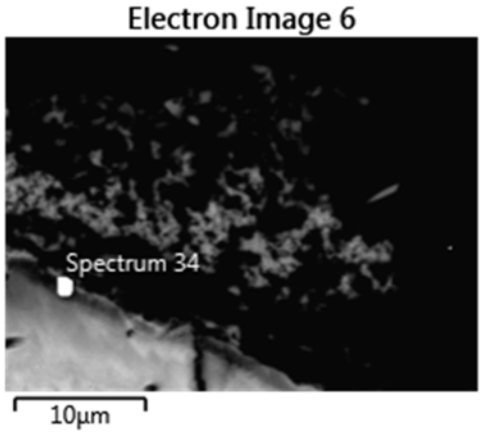

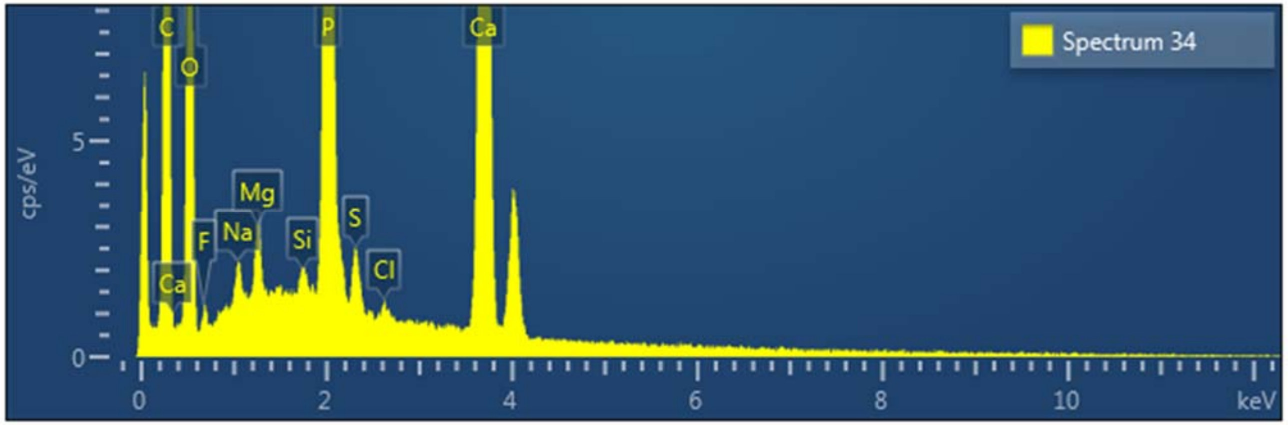

Element	Line Type	Apparent Concentration	k Ratio	Wt%	Wt% Sigma	Standard Label	Factory Standard	Standard Calibration Date
C	K series	2.78	0.02780	37.51	0.30	C Vit	Yes	
O	K series	3.73	0.01256	28.81	0.26	SiO2	Yes	
F	K series	0.19	0.00037	0.82	0.10	CaF2	Yes	
Na	K series	0.10	0.00043	0.38	0.04	Albite	Yes	
Mg	K series	0.14	0.00090	0.57	0.03	MgO	Yes	
Si	K series	0.05	0.00037	0.17	0.03	SiO2	Yes	
P	K series	4.25	0.02375	10.92	0.09	GaP	Yes	
S	K series	0.15	0.00127	0.59	0.03	FeS2	Yes	
Cl	K series	0.04	0.00035	0.16	0.03	NaCl	Yes	
Ca	K series	5.21	0.04652	20.07	0.15	Wollastonite	Yes	
Total:				100.00				

OXFORD INSTRUMENTS
The Business of Science®

Finding F

12/19/2017

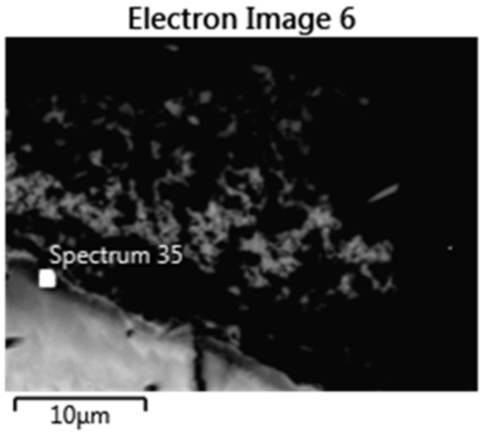

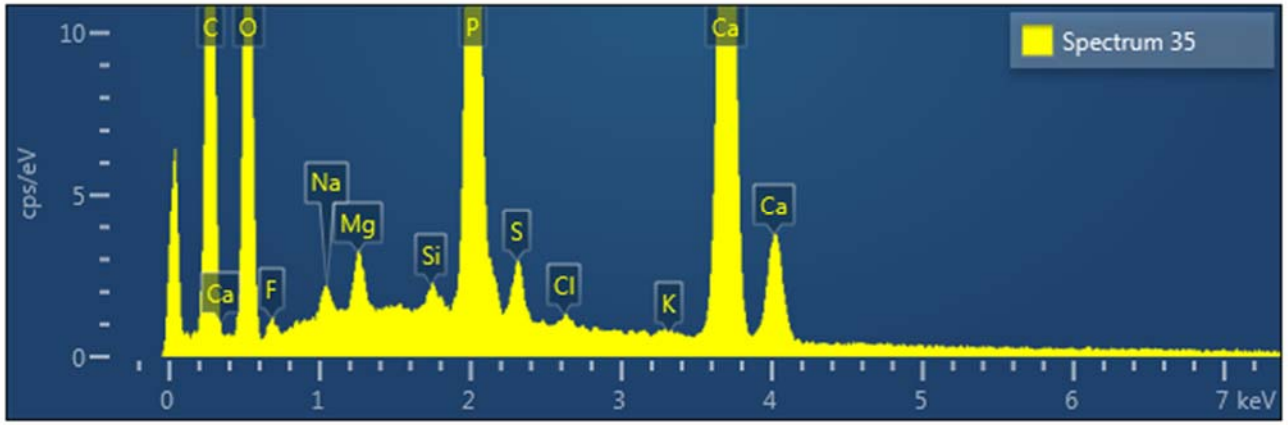

Element	Line Type	Apparent Concentration	k Ratio	Wt%	Wt% Sigma	Standard Label	Factory Standard	Standard Calibration Date
C	K series	3.14	0.03138	18.79	0.22	C Vit	Yes	
O				63.01				
F	K series	0.19	0.00038	0.53	0.07	CaF2	Yes	
Na	K series	0.10	0.00042	0.22	0.02	Albite	Yes	
Mg	K series	0.14	0.00091	0.34	0.02	MgO	Yes	
Si	K series	0.05	0.00041	0.11	0.01	SiO2	Yes	
P	K series	4.33	0.02423	6.01	0.05	GaP	Yes	
S	K series	0.19	0.00164	0.39	0.02	FeS2	Yes	
Cl	K series	0.04	0.00036	0.09	0.01	NaCl	Yes	
K	K series	0.03	0.00024	0.05	0.02	KBr	Yes	
Ca	K series	5.23	0.04676	10.47	0.08	Wollastonite	Yes	
Total:				100.00				

OXFORD INSTRUMENTS
The Business of Science®

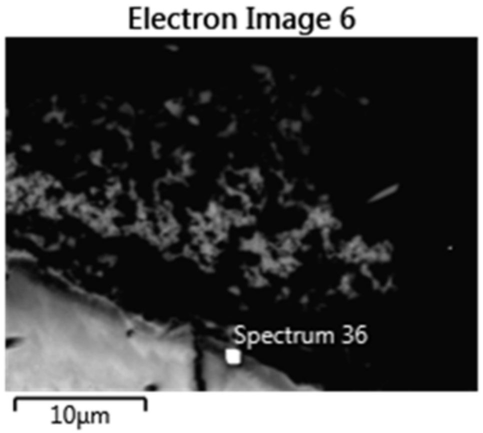

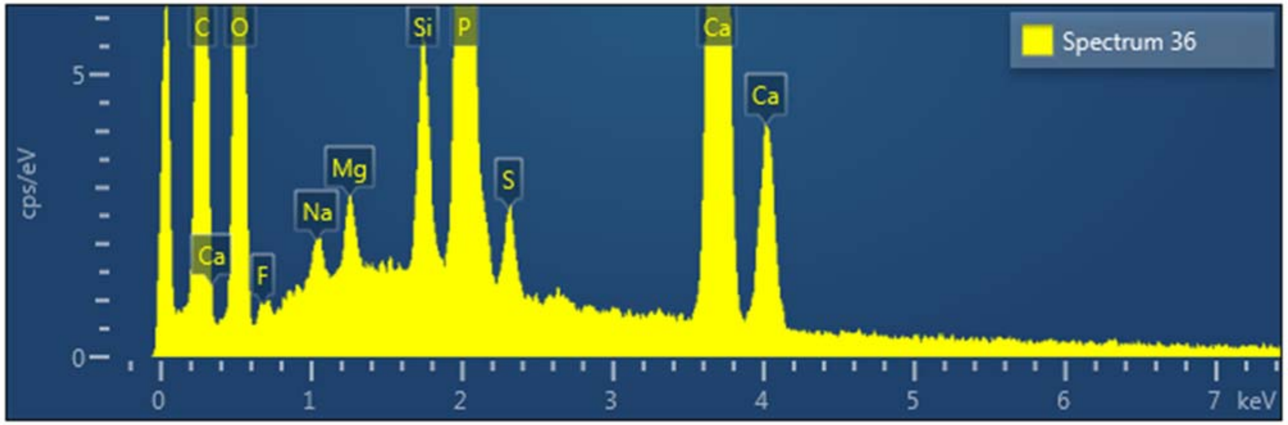

Element	Line Type	Apparent Concentration	k Ratio	Wt%	Wt% Sigma	Standard Label	Factory Standard	Standard Calibration Date
C	K series	3.42	0.03418	39.95	0.27	C Vit	Yes	
O	K series	4.03	0.01355	27.81	0.25	SiO2	Yes	
F	K series	0.12	0.00025	0.48	0.09	CaF2	Yes	
Na	K series	0.09	0.00038	0.29	0.04	Albite	Yes	
Mg	K series	0.10	0.00066	0.37	0.03	MgO	Yes	
Si	K series	0.32	0.00257	1.08	0.03	SiO2	Yes	
P	K series	4.49	0.02510	10.29	0.09	GaP	Yes	
S	K series	0.16	0.00136	0.56	0.03	FeS2	Yes	
Ca	K series	5.60	0.05005	19.17	0.14	Wollastonite	Yes	
Total:				100.00				

OXFORD INSTRUMENTS
The Business of Science®

Finding F

12/19/2017

Electron Image 6

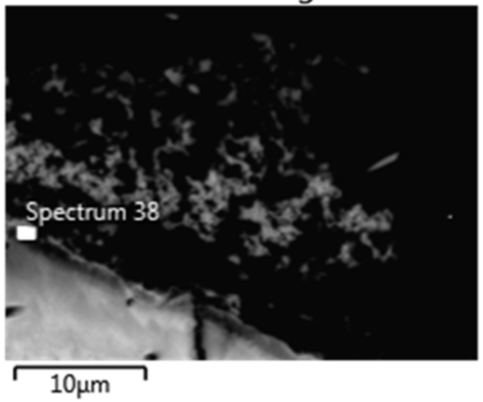

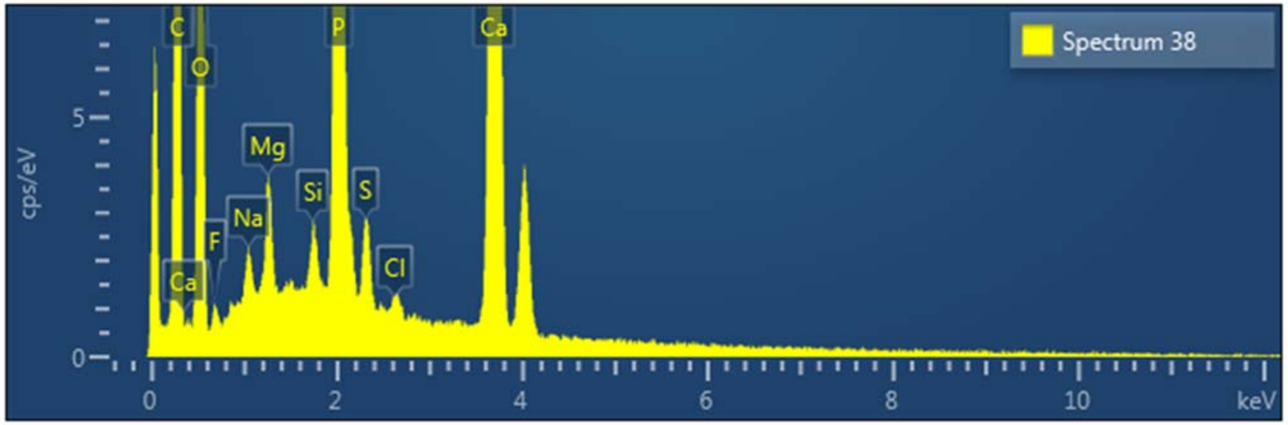

Element	Line Type	Apparent Concentration	k Ratio	Wt%	Wt% Sigma	Standard Label	Factory Standard	Standard Calibration Date
C	K series	2.54	0.02538	37.32	0.31	C Vit	Yes	
O	K series	3.28	0.01105	26.87	0.26	SiO2	Yes	
F	K series	0.18	0.00035	0.79	0.10	CaF2	Yes	
Na	K series	0.11	0.00047	0.42	0.04	Albite	Yes	
Mg	K series	0.18	0.00120	0.79	0.04	MgO	Yes	
Si	K series	0.10	0.00077	0.38	0.03	SiO2	Yes	
P	K series	4.31	0.02413	11.46	0.10	GaP	Yes	
S	K series	0.20	0.00176	0.85	0.04	FeS2	Yes	
Cl	K series	0.05	0.00047	0.22	0.03	NaCl	Yes	
Ca	K series	5.25	0.04689	20.91	0.15	Wollastonite	Yes	
Total:				100.00				

OXFORD INSTRUMENTS
The Business of Science®

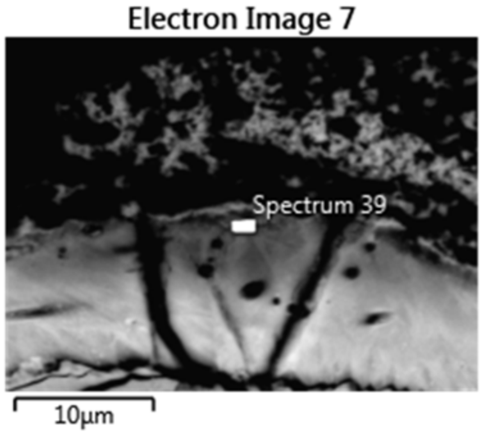

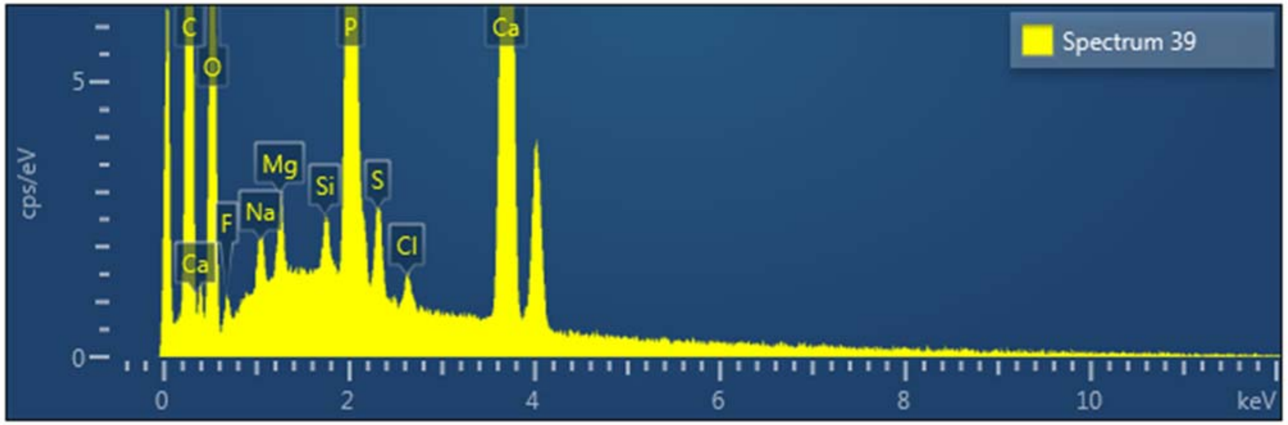

Element	Line Type	Apparent Concentration	k Ratio	Wt%	Wt% Sigma	Standard Label	Factory Standard	Standard Calibration Date
C	K series	3.49	0.03488	19.20	0.17	C Vit	Yes	
O				63.47				
F	K series	0.18	0.00036	0.48	0.06	CaF2	Yes	
Na	K series	0.11	0.00048	0.23	0.02	Albite	Yes	
Mg	K series	0.11	0.00070	0.24	0.02	MgO	Yes	
Si	K series	0.07	0.00055	0.13	0.01	SiO2	Yes	
P	K series	4.42	0.02474	5.73	0.05	GaP	Yes	
S	K series	0.18	0.00155	0.35	0.02	FeS2	Yes	
Cl	K series	0.07	0.00060	0.14	0.01	NaCl	Yes	
Ca	K series	5.37	0.04800	10.03	0.07	Wollastonite	Yes	
Total:				100.00				

Finding F

12/19/2017

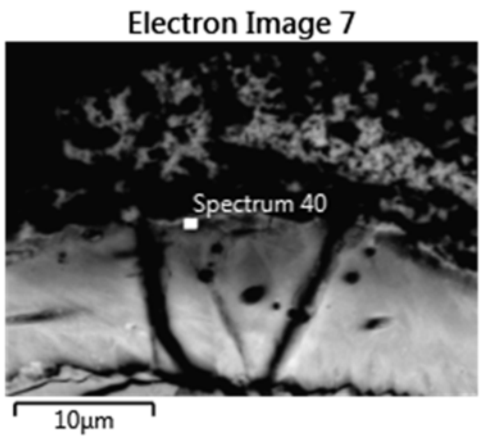

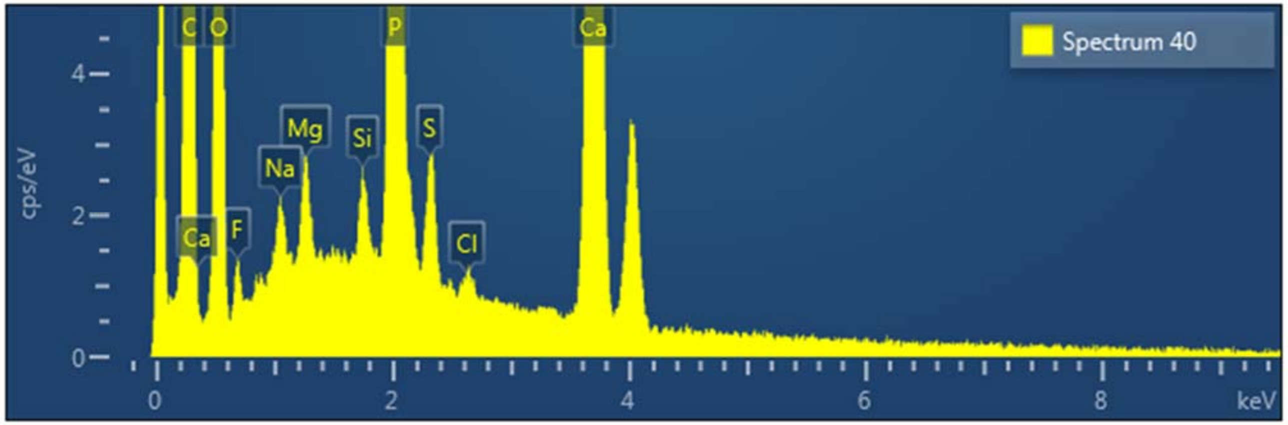

Element	Line Type	Apparent Concentration	k Ratio	Wt%	Wt% Sigma	Standard Label	Factory Standard	Standard Calibration Date
C	K series	4.63	0.04631	48.57	0.26	C Vit	Yes	
O	K series	3.35	0.01127	24.04	0.23	SiO2	Yes	
F	K series	0.24	0.00048	0.91	0.09	CaF2	Yes	
Na	K series	0.09	0.00040	0.30	0.03	Albite	Yes	
Mg	K series	0.11	0.00075	0.42	0.03	MgO	Yes	
Si	K series	0.09	0.00073	0.31	0.02	SiO2	Yes	
P	K series	3.74	0.02092	8.64	0.08	GaP	Yes	
S	K series	0.20	0.00172	0.71	0.03	FeS2	Yes	
Cl	K series	0.04	0.00038	0.16	0.03	NaCl	Yes	
Ca	K series	4.58	0.04090	15.95	0.12	Wollastonite	Yes	
Total:				100.00				

OXFORD INSTRUMENTS
The Business of Science®

Finding F

12/19/2017

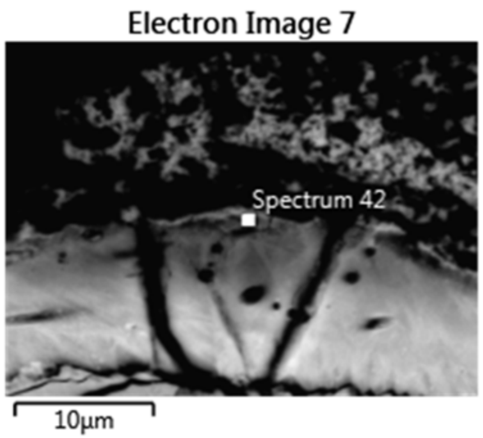

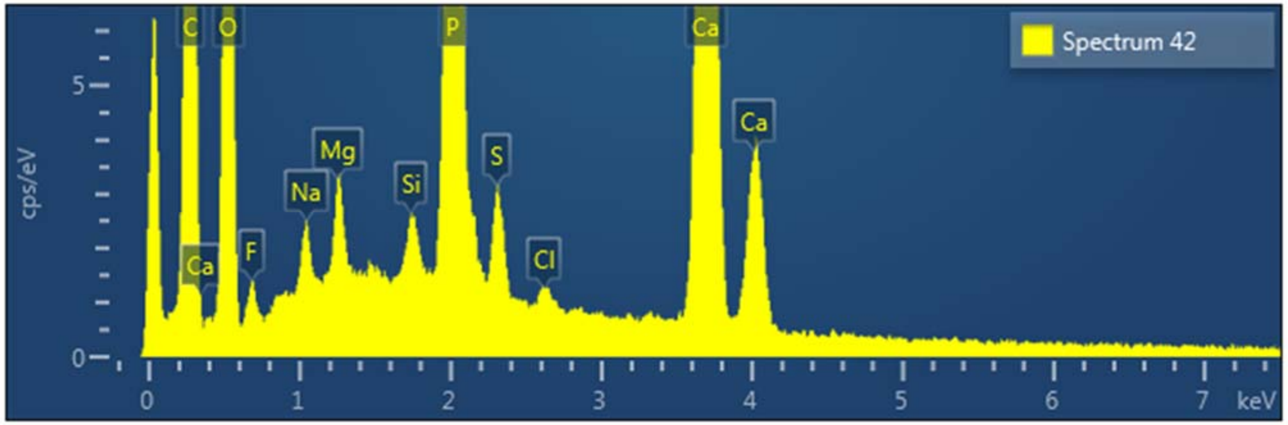

Element	Line Type	Apparent Concentration	k Ratio	Wt%	Wt% Sigma	Standard Label	Factory Standard	Standard Calibration Date
C	K series	3.76	0.03765	43.07	0.28	C Vit	Yes	
O	K series	3.49	0.01173	24.94	0.24	SiO2	Yes	
F	K series	0.27	0.00053	1.01	0.10	CaF2	Yes	
Na	K series	0.13	0.00055	0.42	0.03	Albite	Yes	
Mg	K series	0.13	0.00089	0.50	0.03	MgO	Yes	
Si	K series	0.08	0.00067	0.28	0.03	SiO2	Yes	
P	K series	4.39	0.02453	10.05	0.09	GaP	Yes	
S	K series	0.20	0.00174	0.72	0.03	FeS2	Yes	
Cl	K series	0.05	0.00043	0.18	0.03	NaCl	Yes	
Ca	K series	5.47	0.04883	18.84	0.14	Wollastonite	Yes	
Total:				100.00				

The Business of Science®

Finding F

12/19/2017

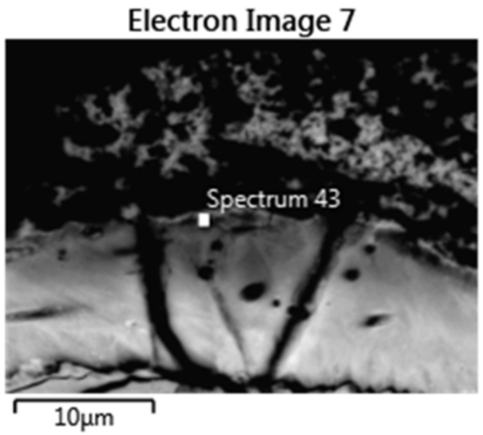

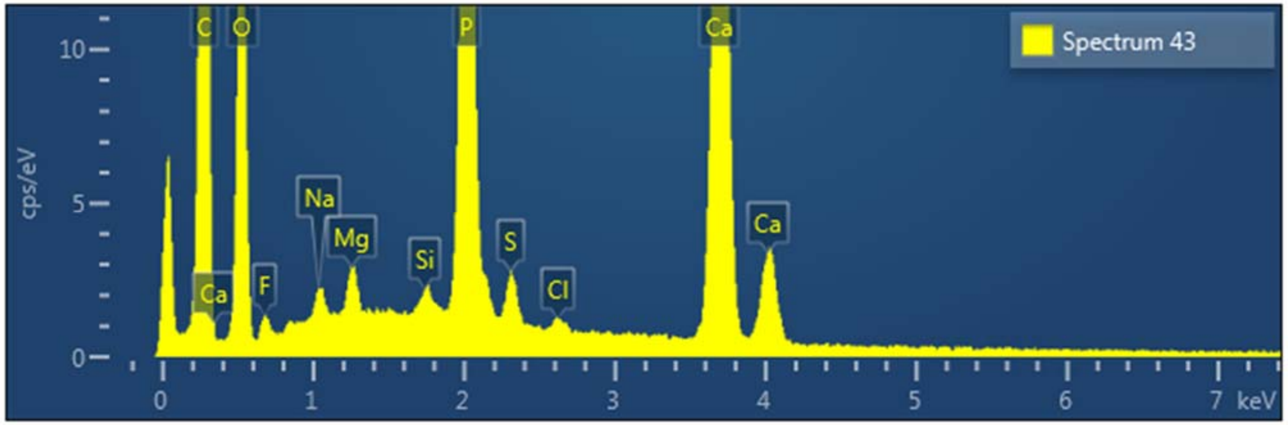

Element	Line Type	Apparent Concentration	k Ratio	Wt%	Wt% Sigma	Standard Label	Factory Standard	Standard Calibration Date
C	K series	4.29	0.04287	47.01	0.27	C Vit	Yes	
O	K series	3.23	0.01088	23.90	0.24	SiO2	Yes	
F	K series	0.26	0.00051	0.99	0.10	CaF2	Yes	
Na	K series	0.11	0.00048	0.37	0.03	Albite	Yes	
Mg	K series	0.12	0.00080	0.46	0.03	MgO	Yes	
Si	K series	0.06	0.00044	0.19	0.02	SiO2	Yes	
P	K series	3.87	0.02166	9.12	0.08	GaP	Yes	
S	K series	0.17	0.00149	0.63	0.03	FeS2	Yes	
Cl	K series	0.05	0.00044	0.19	0.03	NaCl	Yes	
Ca	K series	4.83	0.04315	17.15	0.13	Wollastonite	Yes	
Total:				100.00				

OXFORD INSTRUMENTS
The Business of Science®

Finding F

12/19/2017

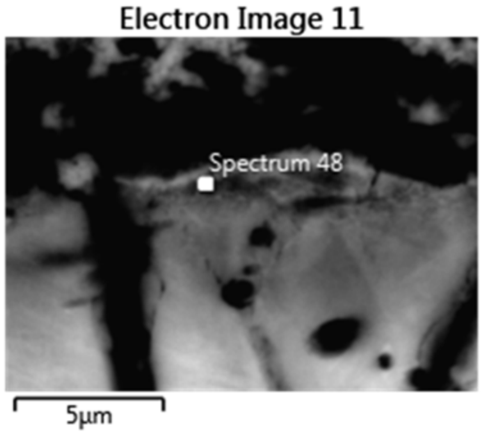

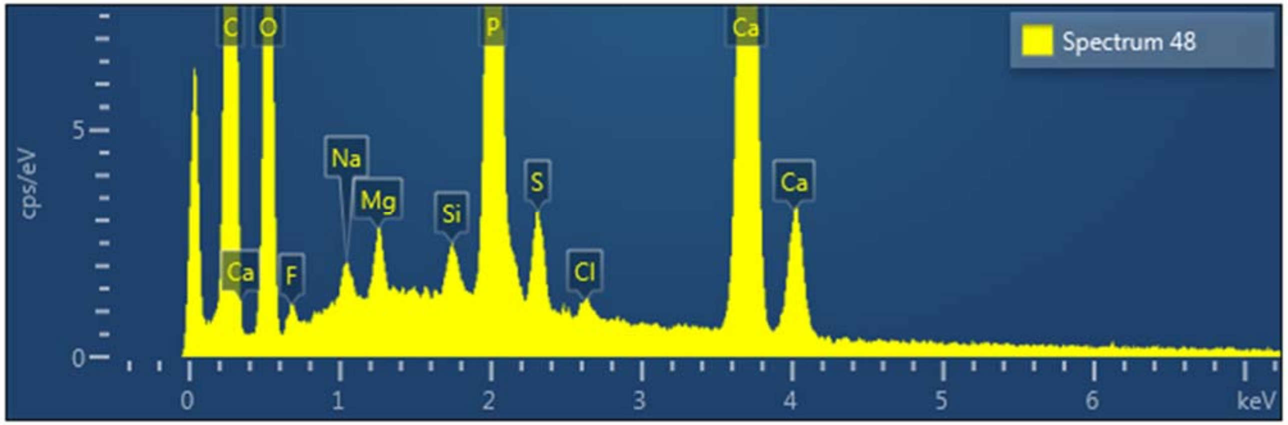

Element	Line Type	Apparent Concentration	k Ratio	Wt%	Wt% Sigma	Standard Label	Factory Standard	Standard Calibration Date
C	K series	4.56	0.04555	50.66	0.26	C Vit	Yes	
O	K series	2.75	0.00924	21.72	0.23	SiO2	Yes	
F	K series	0.18	0.00036	0.72	0.09	CaF2	Yes	
Na	K series	0.09	0.00039	0.31	0.03	Albite	Yes	
Mg	K series	0.11	0.00072	0.43	0.03	MgO	Yes	
Si	K series	0.08	0.00066	0.30	0.03	SiO2	Yes	
P	K series	3.50	0.01957	8.58	0.08	GaP	Yes	
S	K series	0.23	0.00194	0.85	0.03	FeS2	Yes	
Cl	K series	0.05	0.00042	0.18	0.03	NaCl	Yes	
Ca	K series	4.38	0.03914	16.25	0.12	Wollastonite	Yes	
Total:				100.00				

OXFORD INSTRUMENTS
The Business of Science®

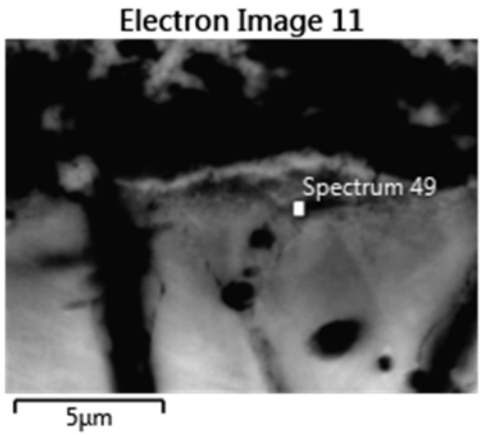

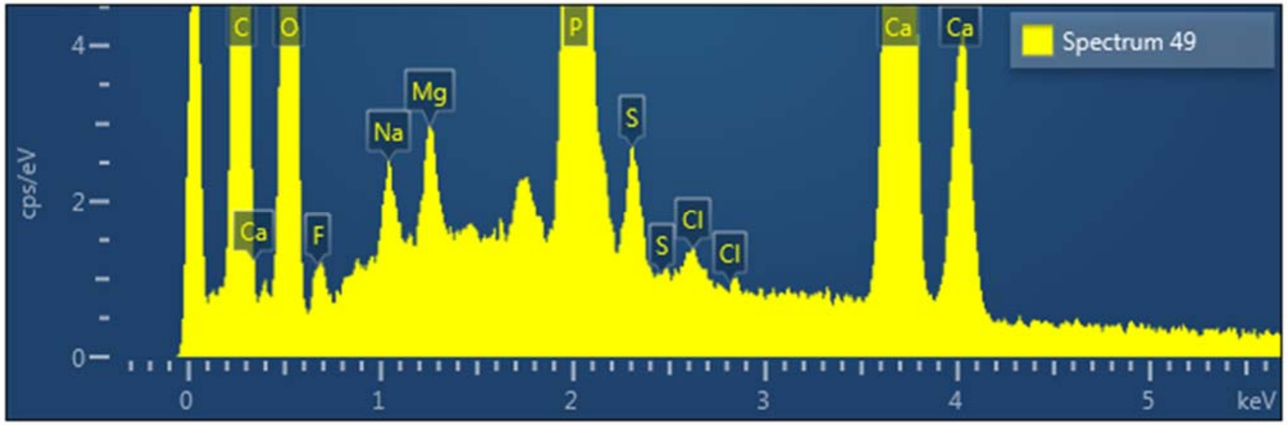

Element	Line Type	Apparent Concentration	k Ratio	Wt%	Wt% Sigma	Standard Label	Factory Standard	Standard Calibration Date
C	K series	3.30	0.03304	40.30	0.28	C Vit	Yes	
O	K series	3.57	0.01201	26.23	0.25	SiO2	Yes	
F	K series	0.21	0.00042	0.84	0.10	CaF2	Yes	
Na	K series	0.12	0.00051	0.41	0.04	Albite	Yes	
Mg	K series	0.11	0.00072	0.42	0.03	MgO	Yes	
P	K series	4.67	0.02610	11.00	0.09	GaP	Yes	
S	K series	0.16	0.00138	0.59	0.03	FeS2	Yes	
Cl	K series	0.04	0.00038	0.16	0.03	NaCl	Yes	
Ca	K series	5.65	0.05045	20.05	0.14	Wollastonite	Yes	
Total:				100.00				

OXFORD INSTRUMENTS
The Business of Science®

Finding F

12/19/2017

Electron Image 11

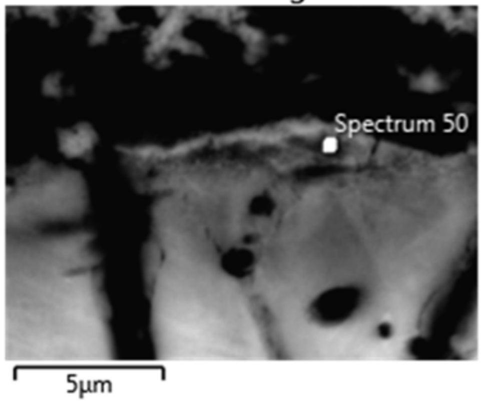

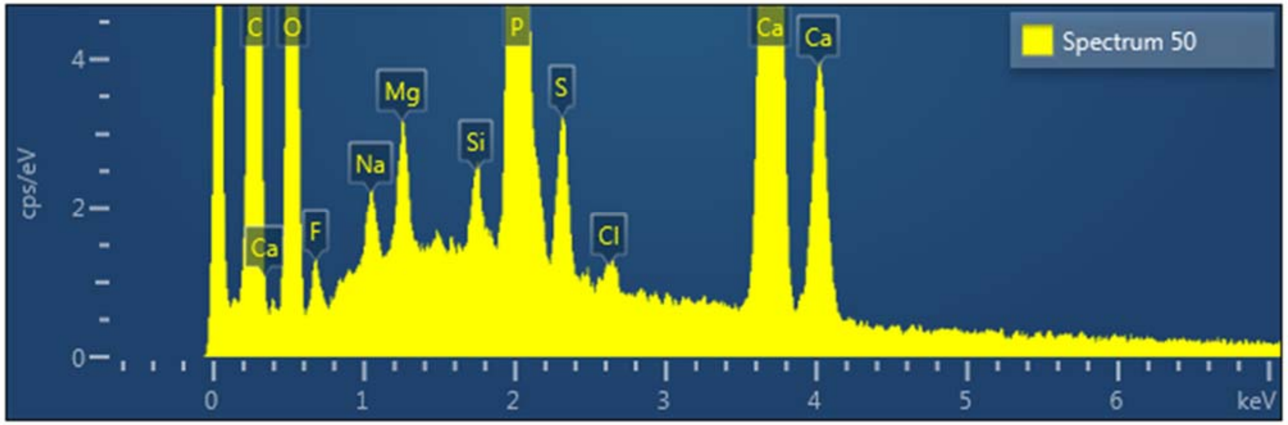

Element	Line Type	Apparent Concentration	k Ratio	Wt%	Wt% Sigma	Standard Label	Factory Standard	Standard Calibration Date
C	K series	3.59	0.03593	44.07	0.28	C Vit	Yes	
O	K series	2.97	0.01001	23.10	0.24	SiO2	Yes	
F	K series	0.23	0.00045	0.90	0.10	CaF2	Yes	
Na	K series	0.11	0.00046	0.37	0.03	Albite	Yes	
Mg	K series	0.13	0.00085	0.50	0.03	MgO	Yes	
Si	K series	0.08	0.00063	0.28	0.03	SiO2	Yes	
P	K series	4.29	0.02398	10.34	0.09	GaP	Yes	
S	K series	0.22	0.00193	0.84	0.03	FeS2	Yes	
Cl	K series	0.05	0.00045	0.20	0.03	NaCl	Yes	
Ca	K series	5.33	0.04765	19.41	0.14	Wollastonite	Yes	
Total:				100.00				
